# TCTP protein degradation by targeting mTORC1 and signaling through S6K, Akt, and Plk1 sensitizes lung cancer cells to DNA-damaging drugs

**DOI:** 10.1038/s41598-021-00247-0

**Published:** 2021-10-21

**Authors:** Mini Jeong, Mi Hyeon Jeong, Jung Eun Kim, Serin Cho, Kyoung Jin Lee, Serkin Park, Jeongwon Sohn, Yun Gyu Park

**Affiliations:** 1 Department of Biochemistry and Molecular Biology, Korea University College of Medicine, 73 Koryodae-ro, Sungbuk-gu, Seoul, 02841 Republic of Korea; 2 Korean Institute of Molecular Medicine and Nutrition, Korea University College of Medicine, Seoul, Republic of Korea; 3 Present Address: Division of Allergy and Clinical Immunology, Department of Internal Medicine, ASAN Medical Center, University of Ulsan College of Medicine, Seoul, Republic of Korea

**Keywords:** Cancer, Molecular medicine

## Abstract

Translationally controlled tumor protein (TCTP) is expressed in many tissues, particularly in human tumors. It plays a role in malignant transformation, apoptosis prevention, and DNA damage repair. The signaling mechanisms underlying TCTP regulation in cancer are only partially understood. Here, we investigated the role of mTORC1 in regulating TCTP protein levels, thereby modulating chemosensitivity, in human lung cancer cells and an A549 lung cancer xenograft model. The inhibition of mTORC1, but not mTORC2, induced ubiquitin/proteasome-dependent TCTP degradation without a decrease in the mRNA level. PLK1 activity was required for TCTP ubiquitination and degradation and for its phosphorylation at Ser^46^ upon mTORC1 inhibition. Akt phosphorylation and activation was indispensable for rapamycin-induced TCTP degradation and PLK1 activation, and depended on S6K inhibition, but not mTORC2 activation. Furthermore, the minimal dose of rapamycin required to induce TCTP proteolysis enhanced the efficacy of DNA-damaging drugs, such as cisplatin and doxorubicin, through the induction of apoptotic cell death in vitro and in vivo. This synergistic cytotoxicity of these drugs was induced irrespective of the functional status of p53. These results demonstrate a new mechanism of TCTP regulation in which the mTORC1/S6K pathway inhibits a novel Akt/PLK1 signaling axis and thereby induces TCTP protein stabilization and confers resistance to DNA-damaging agents. The results of this study suggest a new therapeutic strategy for enhancing chemosensitivity in lung cancers regardless of the functional status of p53.

## Introduction

The translationally controlled tumor protein (TCTP), also known as an IgE-dependent histamine releasing factor, is highly conserved in diverse eukaryotic organisms including yeast, fungi, nematodes, fruit flies, mice, humans, and plants^1–4^. However, the expression level of TCTP varies widely depending on cell or tissue type and the developmental stage. TCTP plays a role in both physiologic and pathologic cellular processes, such as cell proliferation, cell growth, cell death, response to various cellular stresses, and malignant transformation^1–9^. In fact, TCTP is highly expressed in human cancer cell lines of various tissue origins as well as human tumor tissues and sera from cancer patients^1,3,4,10,11^. TCTP is down-regulated during reversion from the malignant to normal phenotype suggesting that TCTP could be a target for tumor reversion^2,10,12,13^. Recently, it has been demonstrated that the TCTP protein promotes the degradation of p53 protein, stabilization of myeloid leukemia cell differentiation protein 1 (Mcl-1) protein, and inhibition of Bax dimerization, all of which promote cell survival^7,13–17^ and explain how increased expression of TCTP in cancer promotes tumor growth and maintenance. TCTP expression is regulated by diverse extracellular signals and intracellular conditions, such as growth signals, cytokines, cellular stresses, and cell death signals^3,4^. The mechanisms involved in TCTP regulation were elucidated in studies of physiologic processes or responses in a variety of normal cells and tissues of various organisms as well as those of increased TCTP expression in tumor cells^3,4^. Translational regulation of TCTP expression is well documented^3,4^. mTORC1 activates the preferential translation of mRNAs containing a 5′ terminal oligopyrimidine tract of which TCTP mRNA is a well-known member^18–20^ and positively regulates TCTP mRNA translation in human cancer cells^21,22^. However, the precise signaling mechanisms underlying the increased expression of TCTP in tumor cells are only partially understood. Little is known about the regulation of TCTP protein degradation, especially its regulation by mTORC1.

TCTP interacts with various cellular proteins with different functions^2,13^ including p53^14,15^, mouse double minute 2 homolog (Mdm2)^15^, Mcl-1^1,16^, and polo-like kinase 1 (PLK1)^23–25^. Of these, PLK1, a key regulator of mitotic phase progression, is known to phosphorylate TCTP^23–27^. The C-terminal polo-box domain (PBD) of PLK1 is required for substrate recognition and subcellular localization of the enzyme^24,28^. TCTP contains not only PLK1-dependent phosphorylation sites (Ser^46^ and Ser^64^), but also a potential PBD-binding site (Thr^65^), which is a priming phosphorylation site^23–27^. TCTP phosphorylation is significantly induced at the G_2_/M phase of the cell cycle during which the protein level and kinase activity of PLK1 reach a maximum^23,25,27^. However, whether PLK1 activity or TCTP phosphorylation is involved in regulating TCTP protein levels remains unclear. Mammalian target of rapamycin (mTOR) inhibition by rapamycin results in feedback activation of serine/threonine protein kinase B (Akt) via the ribosomal protein S6 kinase (S6K)^29–32^. Although it has been suggested that Akt may contribute to the phosphorylation and activation of PLK1^33,34^, it has not been experimentally demonstrated.

In a preliminary screening study, we identified rapamycin, a potent mTOR complex 1 (mTORC1) inhibitor, as a negative regulator of TCTP levels. In the present study, we elucidated the signaling mechanism by which mTORC1 regulates TCTP levels in lung cancer cells. We demonstrated that Ser^46^ phosphorylation of TCTP by PLK1 (and possibly phosphorylation at Ser^64^ and Thr^65^) are required for TCTP protein degradation by mTORC1 inhibition. We also found that PLK1 is negatively and positively regulated by mTORC1/S6K and Akt, respectively. In conclusion, mTORC1/S6K/Akt/PLK1 signaling plays a critical role in maintaining the stability of TCTP protein in lung cancer cells, thereby conferring resistance to DNA-damaging anticancer drugs.

## Results

### Inhibition of mTORC1 activity reduces TCTP protein but not mRNA levels

**Figure 1:**
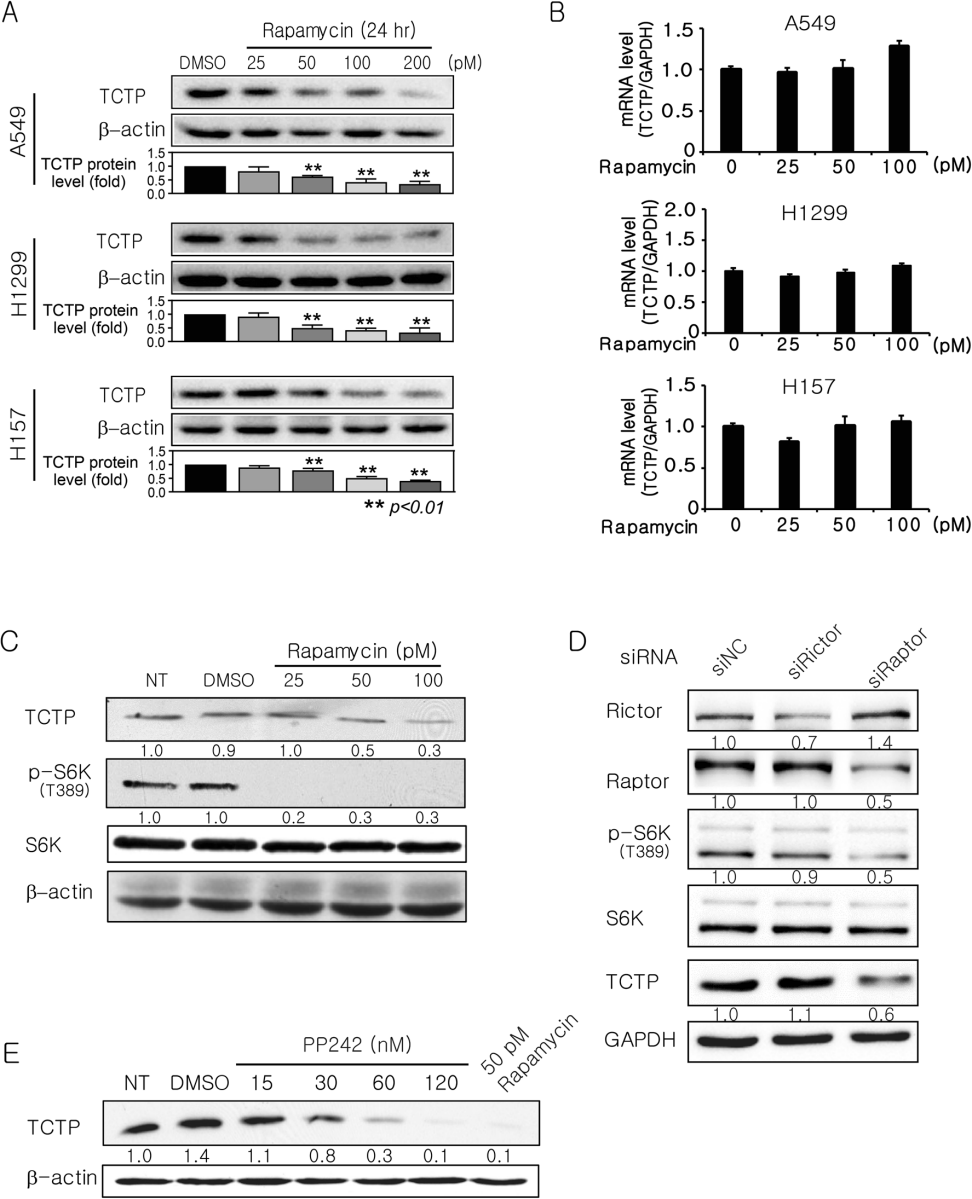
mTORC1 inhibition decreases the levels of TCTP protein, but not mRNA. (**A**) Rapamycin reduced the levels of TCTP protein (n = 4). (**B**) Rapamycin did not decrease the levels of TCTP mRNA (n = 3). Cells were harvested 24 h after rapamycin treatment (**A**,**B**). (**C**) Inhibition of mTORC1 activity was confirmed by determining S6K phosphorylation at Thr389. A549 cells were harvested 24 h after treatment with rapamycin (n = 3). (**D**) Targeting Raptor, but not Rictor, decreased TCTP expression and S6K phosphorylation. A549 cells were harvested 48 h after transfection with 100 pM siRNA (n = 3). (**E**) 24-h treatment with PP242, an inhibitor of both mTORC1 and mTORC2 reduced TCTP protein levels in A549 cells (n = 4). Total protein was analyzed by immunoblotting (**A**,**C**–**E**) and total mRNAs were analyzed by qRT-PCR (**B**). The bar graphs in (**A**) and (**B**) indicate the average of four and three independent experiments, respectively. The band intensities were quantified and normalized to those of internal controls or the total forms, and fold changes compared with controls are presented as numbers below the bands. The proteins and mRNA of β-actin and GAPDH were used as internal controls. DMSO (≤ 0.1%) was used as a vehicle control. NT, DMSO, and NC indicate no treatment, dimethyl sulfoxide, and negative control, respectively.

In preliminary experiments, we measured TCTP protein levels in various lung cancer cells including human lung carcinogenesis model cell lines (BEAS-2B, 1799, 1198, and 1170-I cells) (Supplementary Fig. S1). For all subsequent experiments, we selected cells (A549, H1299, and H157 cells) with higher levels of TCTP protein compared with BEAS-2B (immortalized) normal human bronchial epithelium cells. To explore the effect of mTORC1 inhibition on TCTP expression in human lung cancer cells (A549, H1299, and H157), the cells were treated with rapamycin at various concentrations. In all cell lines, rapamycin treatment dramatically reduced TCTP protein levels in a dose-dependent manner (Fig. 1A). 50 or 100 pM rapamycin was selected for subsequent experiments. TCTP mRNA levels were not decreased as determined by quantitative real time (qRT)-polymerase chain reaction (PCR) (Fig. 1B). To confirm that mTORC1 activity was inhibited, we examined the phosphorylation status of S6K (an mTORC1 substrate) and/or S6 (a S6K substrate). As previously reported^31,32^, rapamycin treatment and Raptor siRNA transfection markedly decreased S6K phosphorylation at Thr^389^ (Fig. 1C,D, lanes 1 and 3). To ensure that the high level of TCTP protein in lung cancer cells was mTORC1-dependent, mTOR activity was inhibited using siRNAs targeting Raptor and Rictor, components of the mTORC1 and mTOR complex 2 (mTORC2), respectively, or PP242, an mTOR kinase inhibitor targeting both mTORC1 and mTORC2. In A549 cells, Raptor siRNA transfection (Fig. 1D, lanes 1 and 3) and PP242 treatment (Fig. 1E) reduced the level of TCTP protein, whereas Rictor knockdown did not (Fig. 1D, lanes 1 and 2). These results indicate that mTORC1, but not mTORC2, plays an important role in maintaining a high protein level of TCTP, which is commonly observed in lung cancer cells.

### mTORC1 inhibition induces degradation of TCTP protein through the ubiquitin–proteasome system

**Figure 2:**
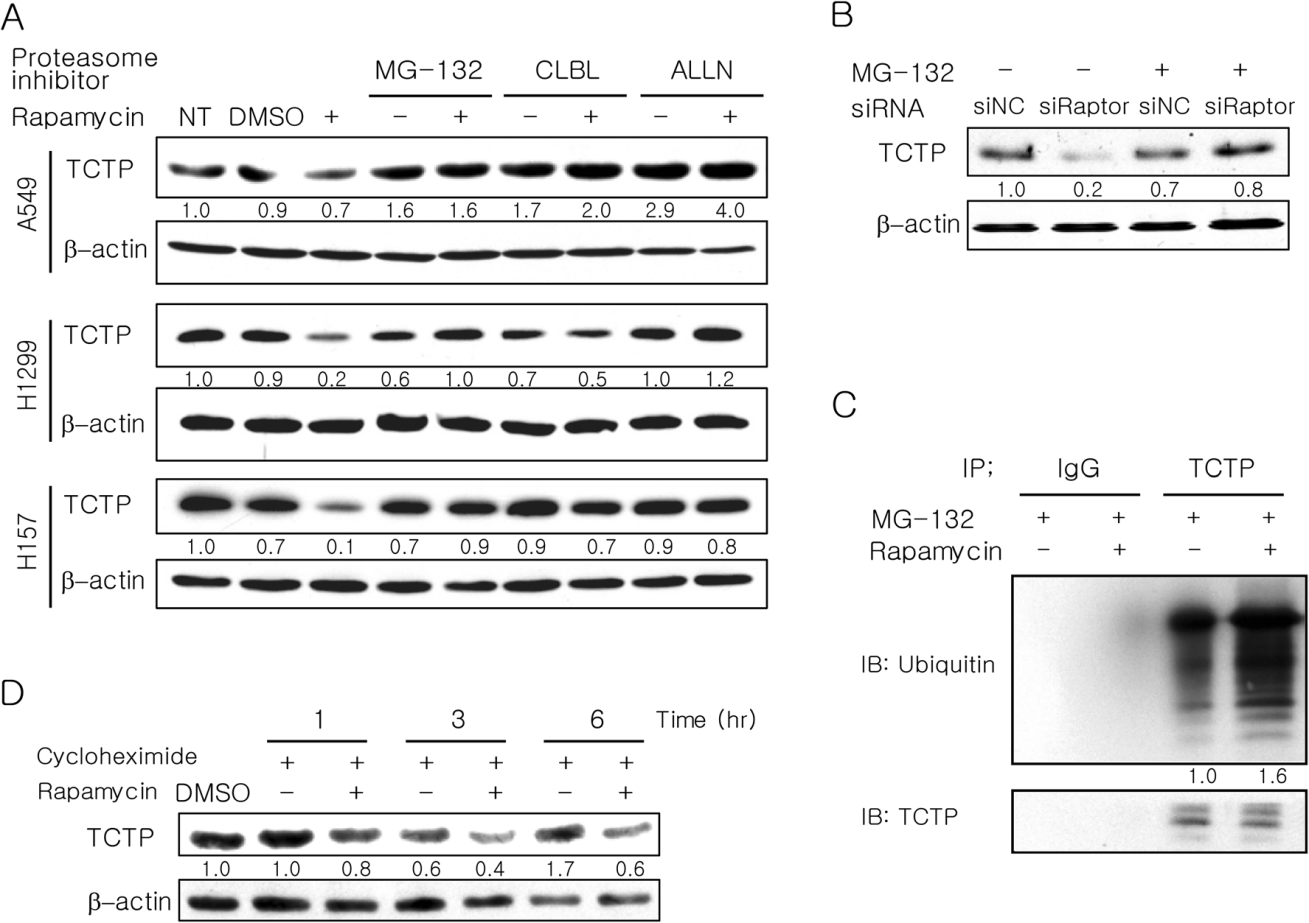
Reduction in TCTP protein levels upon mTORC1 inhibition is completely blocked by proteasome inhibition and is accompanied by increased TCTP ubiquitination. (**A**,**B**) Reduction in TCTP protein levels upon mTORC1 inhibition by rapamycin or Raptor siRNA was blocked by various proteasome inhibitors. (**A**) Cells were preincubated for 2 h with 20 μM MG-132, 50 μM CLBL, and 100 μM ALLN and then treated for 24 h with 50 pM rapamycin (n = 4). (**B**) A549 cells were transfected for 4 h with 100 pM siRNA and treated with 20 μM MG-132 for 24 h. (**C**) TCTP ubiquitination was increased by rapamycin treatment. A549 cells were treated with 50 pM rapamycin in the presence of 20 µM MG-132 for 24 h. The cell lysates were immunoprecipitated (IP) with anti-TCTP antibody or normal anti-rabbit IgG antibody (IgG), and the IP proteins were analyzed by immunoblotting (IB) with antibodies against ubiquitin or TCTP. (**D**) The rapamycin-induced decrease in TCTP protein was not blocked by cycloheximide, an inhibitor of protein synthesis. A549 cells were co-treated with 50 pM rapamycin and 20 µg/ml of cycloheximide. Total protein was analyzed by IB (**A**,**B**,**D**). The band intensities were quantified and normalized to those of internal controls or TCTP (**D**), and fold changes compared to controls are presented as numbers below the bands. β-Actin protein was used as an internal control. DMSO (≤ 0.1%) was used as a vehicle control. Triplicate experiments were performed except when the number of experiments was denoted. NT, DMSO, and NC indicate no treatment, dimethyl sulfoxide, and negative control, respectively.

To investigate whether mTORC1 regulates TCTP expression at the post-translational level, we evaluated proteasome-dependent degradation of TCTP using various proteasome inhibitors [MG-132, clasto-lactacystin β-lactone (CLBL), and acetyl-l-leucyl-l-leucyl-norleucinal (ALLN)]. When lung cancer cells were pretreated with proteasome inhibitors, the reduction of TCTP protein level caused by rapamycin was completely prevented (Fig. 2A). Similarly, MG-132 treatment of A549 cells transfected with Raptor siRNA also blocked the decrease in TCTP levels (Fig. 2B). Furthermore, TCTP ubiquitination was increased by rapamycin treatment (Fig. 2C). To rule out the possibility that mTORC1 inhibition decreases the TCTP protein level by inhibiting TCTP translation, we determined whether cycloheximide, an inhibitor of protein synthesis, blocked the rapamycin-induced decrease in TCTP protein level. Cells were co-treated with rapamycin and cycloheximide for 1, 3, or 6 h (Fig. 2D). Despite cycloheximide co-treatment, the level of TCTP protein was still reduced by rapamycin treatment, seemingly in a time-dependent manner. Thus, the effect of rapamycin on cellular TCTP levels was not affected by cycloheximide. This demonstrates that mTORC1 regulates TCTP expression at the post-translational level. Together, these data reveal that mTORC1 inhibition reduces TCTP protein levels through ubiquitin-proteasomal degradation.

### PLK1 activity is required for TCTP protein degradation by mTORC1 inhibition

**Figure 3:**
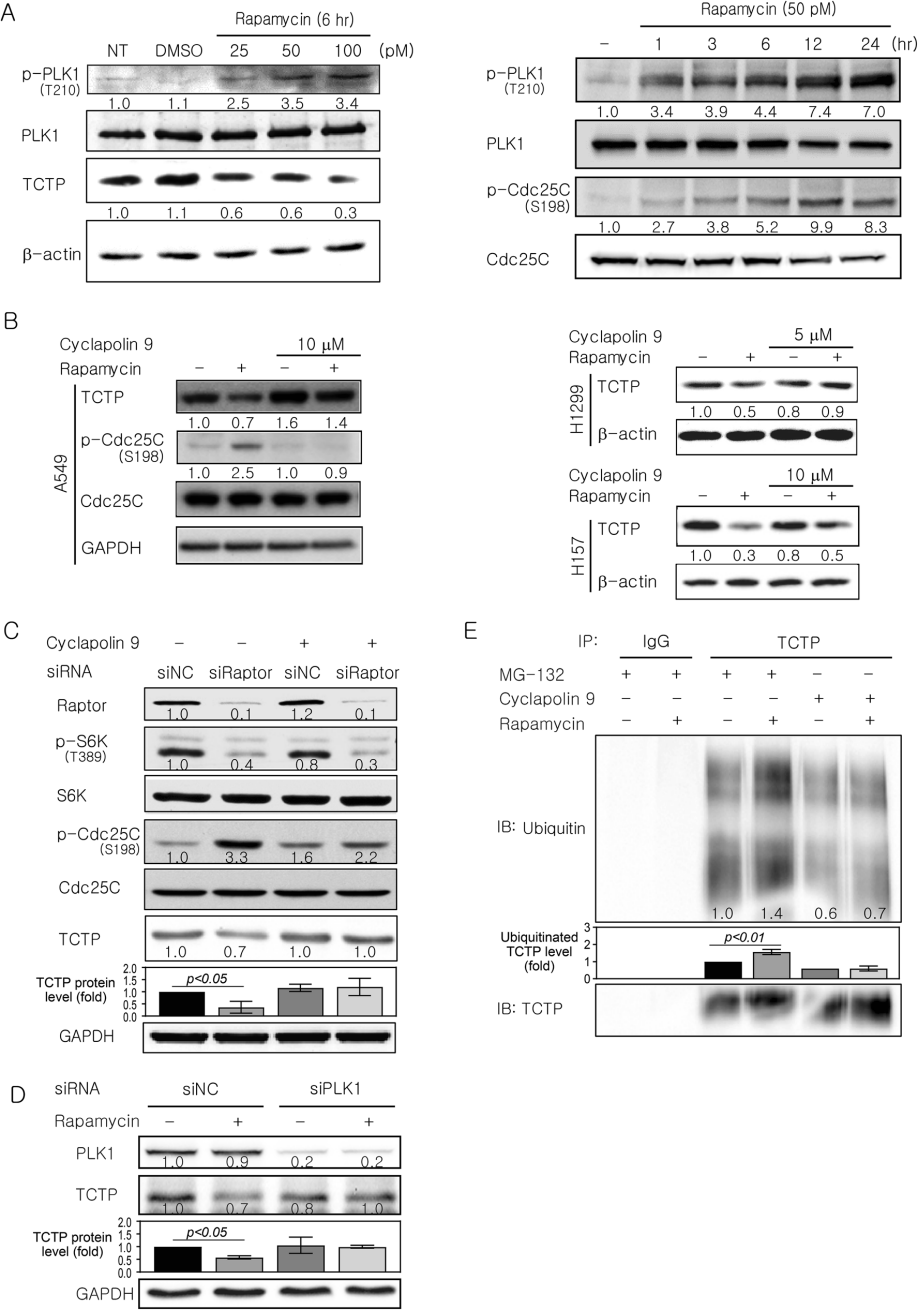
TCTP protein degradation upon mTORC1 inhibition is accompanied by an increase in PLK1 phosphorylation at Thr^210^ and abolished by inhibiting PLK1 activation. (**A**) PLK1 phosphorylation and activation was induced in A549 cells after rapamycin treatment. (**B**,**C**) The decrease in TCTP protein levels caused by rapamycin treatment or raptor knockdown was abrogated by PLK1 inhibition. (**B**) Cells were pretreated with cyclapolin 9, a PLK1 inhibitor, for 2 h and then treated for 12 h with 50 pM rapamycin (n = 4). (**C**) A549 cells were treated with 5 μM cyclapolin 9 beginning 4 h after transfection with 100 pM siRNA and then harvested 48 h after transfection. (**D**) The decrease in TCTP protein levels by rapamycin treatment was abolished by PLK1 knockdown. A549 cells were transfected for 4 h with 100 pM siRNA targeting PLK1 or GFP (as a negative control) and then treated for 12 h with 50 pM rapamycin beginning 48 h after transfection (n = 4). (**E**) The increased ubiquitination of TCTP by rapamycin disappeared upon PLK1 inhibition. A549 cells were preincubated for 2 h with 20 μM MG-132 or 5 μM cyclapolin 9 and then treated for 24 h with 50 pM rapamycin and TCTP ubiquitination was detected as described in Fig. 2C. The band intensities were quantified and normalized to those of internal controls, the total forms, or TCTP (**E**), and fold changes compared with controls are presented as numbers below the bands. The bar graphs in (**C**)–(**E**) indicate the average of three, four, and three independent experiments, respectively. β-actin and GAPDH proteins were used as an internal control. DMSO (≤ 0.1%) was used as a vehicle control. Triplicate experiments were performed except when the number of experiments was denoted. NT, DMSO, and NC indicate no treatment, dimethyl sulfoxide, and negative control, respectively.

TCTP can interact with and be phosphorylated by PLK1^23–25,27^. Therefore, we hypothesized that PLK1 mediates TCTP degradation upon mTORC1 inhibition. PLK1 is activated by phosphorylation at Thr^210^^24,28^ and active PLK1 phosphorylates Cdc25C at Ser^198^^24,35^. To determine whether the inhibition of mTORC1 induces Thr^210^ phosphorylation of PLK1, we examined PLK1 phosphorylation in A549 cells treated with rapamycin. PLK1 phosphorylation at Thr^210^ increased markedly in a dose- and time-dependent manner after rapamycin treatment (Fig. 3A). To present additional evidence that PLK1 was activated, we examined Cdc25C phosphorylation at Ser^198^. When mTORC1 was inhibited by rapamycin treatment, Cdc25C phosphorylation increased in a time-dependent manner (Fig. 3A, right panel).

PLK1 activity was then inhibited by cyclapolin 9, a potent and highly specific PLK1 inhibitor, or PLK1 siRNA. The effect of PLK1 inhibition on cellular TCTP level was assessed after mTORC1 inhibition by rapamycin or Raptor siRNA (Fig. 3B,C). The decrease in TCTP protein levels caused by mTORC1 inhibition was abrogated by cyclapolin 9 pretreatment in A549, H1299, and H157 cells (Fig. 3B) and inhibition of PLK1 activity by the inhibitor was confirmed by a significant change in the phosphorylation of Cdc25C at Ser^198^ (Fig. 3B, left panel and Fig. 3C). A similar result was obtained following siRNA-mediated PLK1 knockdown prior to rapamycin exposure (Fig. 3D). Moreover, the increased ubiquitination of TCTP by rapamycin treatment disappeared with cyclapolin 9 pretreatment (Fig. 3E). These experiments indicate that PLK1 activation is crucial for TCTP degradation induced by mTORC1 inhibition.

### PLK1-induced phosphorylation of TCTP at Ser^46^ and intact Ser^64^ and Thr^65^ of TCTP are necessary for TCTP degradation upon mTORC1 inhibition

**Figure 4:**
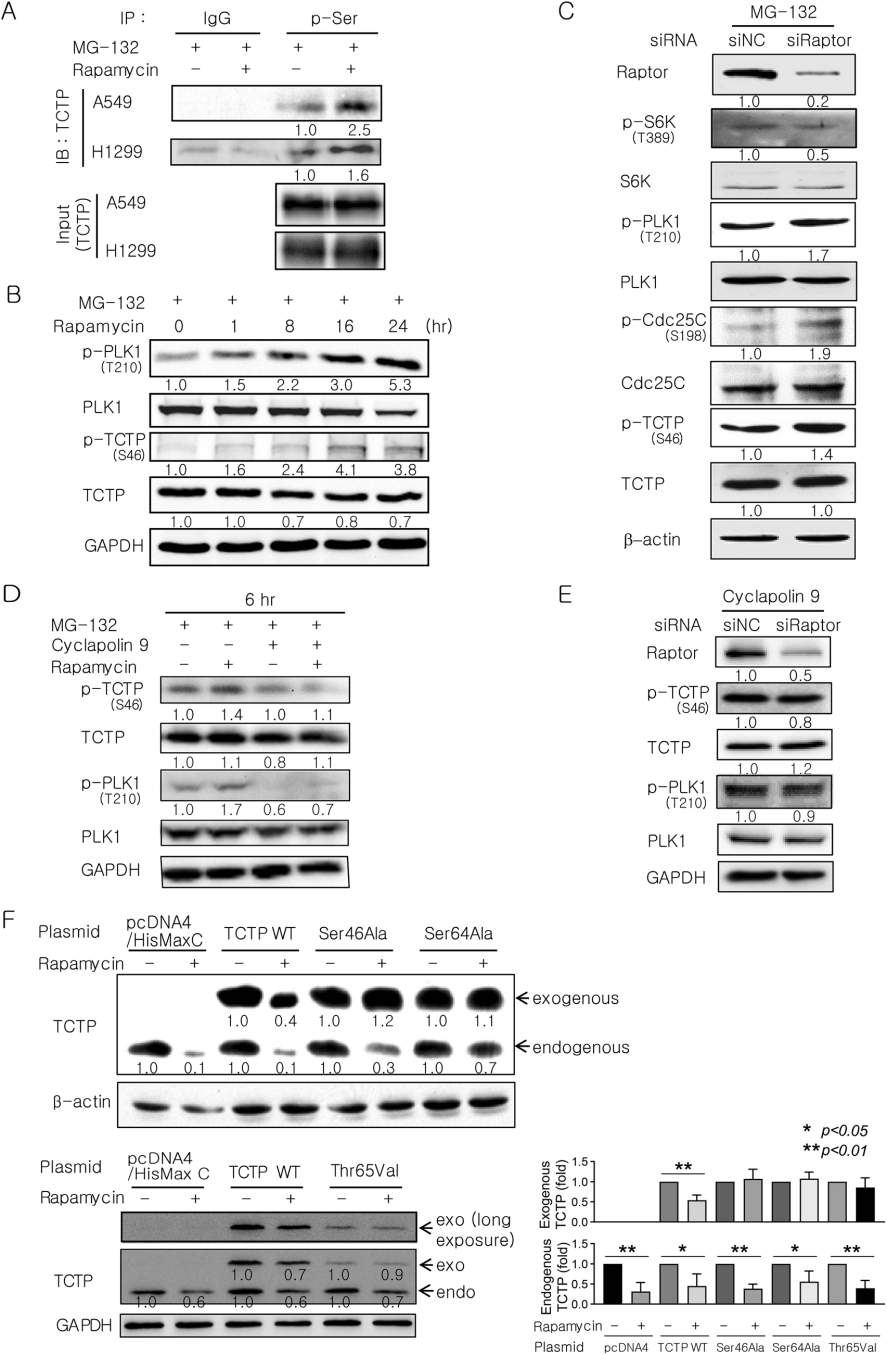
Phosphorylation of TCTP at Ser^46^, Ser^64^, and Thr^65^ is required for TCTP degradation following mTORC1 inhibition. (**A**) Serine phosphorylation of TCTP was increased by rapamycin (n = 2). Cells were treated with 20 μM MG-132 for 2 h followed by 50 pM rapamycin for 12 h. Cell extracts were subjected to immunoprecipitation (IP) with anti-phospho-serine antibody or normal anti-rabbit IgG antibody (IgG) and the IP proteins were analyzed by immunoblotting (IB) with anti-TCTP antibody. The input of TCTP protein was similar. (**B**) The phosphorylation of TCTP at Ser^46^ increased in a time-dependent manner following rapamycin treatment. A549 cells were treated for 2 h with 20 μM MG-132 and then with 50 pM rapamycin. (**C**) mTORC1 inhibition by Raptor siRNA stimulated the phosphorylation of TCTP at Ser^46^. A549 cells were treated with 20 μM MG-132 beginning 4 h after transfection with 100 pM siRNA and harvested 48 h after transfection. (**D**,**E**) The increase in TCTP phosphorylation at Ser^46^ upon mTORC1 inhibition with rapamycin or Raptor siRNA was blocked by inhibiting PLK1 activity. (**D**) A549 cells were treated for 2 h with 5 μM cyclapolin 9 in the presence of 20 μM MG-132 and then with 50 pM rapamycin (n = 4). (**E**) A549 cells were treated with 5 μM cyclapolin 9 beginning 4 h after transfection with 100 pM siRNA and harvested 48 h after transfection. (**F**) TCTP phosphorylation at Ser^46^, Ser^64^, and Thr^65^ was a prerequisite for TCTP protein degradation upon rapamycin treatment (n = 4). A549 cells were transfected for 36–48 h with 4–5.5 μg of plasmids expressing His-tagged wild-type (WT) or phospho-mutants of TCTP (Ser46Ala, Ser64Ala, and Thr65Val) or empty vector (pcDNA4/HisMax C) and then treated with 100 pM rapamycin for 12 h. The bar graphs indicate the average of four independent experiments. The cells were pretreated with MG-132 to prevent the degradation of phosphorylated TCTP (**A**–**D**). Cell lysates were analyzed by IB using the indicated antibodies (**B**–**F**). The band intensities were quantified and normalized to those of internal controls, the total forms, or TCTP (**A**), and fold changes compared with controls are presented as numbers below the bands. β-Actin and GAPDH proteins were used as internal controls. Triplicate experiments were performed except when the number of experiments was denoted.

It has been reported that PLK1 phosphorylates TCTP at Ser^46^ and Ser^64^ and TCTP also contains a potential PBD-binding site at Thr^65^^23–25,27^. Thus, we evaluated changes in TCTP phosphorylation at serine residues in two lung cancer cell lines (A549 and H1299) that were treated with rapamycin. The cells were pretreated with MG-132 to prevent the degradation of phosphorylated TCTP. Serine phosphorylation of TCTP was analyzed by immunoprecipitation and subsequent immunoblotting using antibodies against phosphoserine and TCTP, respectively. It was observed that serine phosphorylation of TCTP was significantly increased by rapamycin in both cell lines (Fig. 4A).

To explore the possibility that mTORC1 inhibition activates PLK1, which then phosphorylates TCTP at Ser^46^, we first investigated whether mTORC1 inhibition with rapamycin or Raptor siRNA stimulated the phosphorylation of TCTP at Ser^46^. Phosphorylation of TCTP was significantly increased in a time-dependent manner following rapamycin treatment, which was similar to the pattern of PLK1 activation in the right panel of Fig. 3A (Fig. 4B). Gene silencing of Raptor by siRNA also stimulated the phosphorylation of TCTP at Ser^46^, PLK1 at Thr^210^, and Cdc25C at Ser^198^ (Fig. 4C). Furthermore, the increase in TCTP phosphorylation at Ser^46^ upon mTORC1 inhibition with rapamycin (Fig. 4D) or Raptor siRNA (Fig. 4E) was completely blocked by cyclapolin 9. This strongly indicates that PLK1 phosphorylates TCTP at Ser^46^ upon mTORC1 inhibition.

We also investigated whether TCTP phosphorylation at PLK1-dependent phosphorylation sites (Ser^46^ and Ser^64^) and a priming phosphorylation site (Thr^65^) affected rapamycin-induced degradation of the TCTP protein. A549 cells were transfected with His-tagged TCTP mutants containing Ser46Ala, Ser64Ala, or Thr65Val mutations and then treated with rapamycin. The protein levels of all these non-phosphorylatable mutants were unchanged by rapamycin treatment, whereas those of the endogenous TCTP and exogenous His-tagged wild-type TCTP decreased significantly (Fig. 4F). Taken together, these findings demonstrate that PLK1 activation and TCTP phosphorylation at Ser^46^, Ser^64^, and Thr^65^ are prerequisites for proteasome-mediated TCTP protein degradation upon mTORC1 inhibition.

### Akt activation following S6K inhibition plays a key role in rapamycin-induced TCTP protein degradation through PLK1 activation

**Figure 5:**
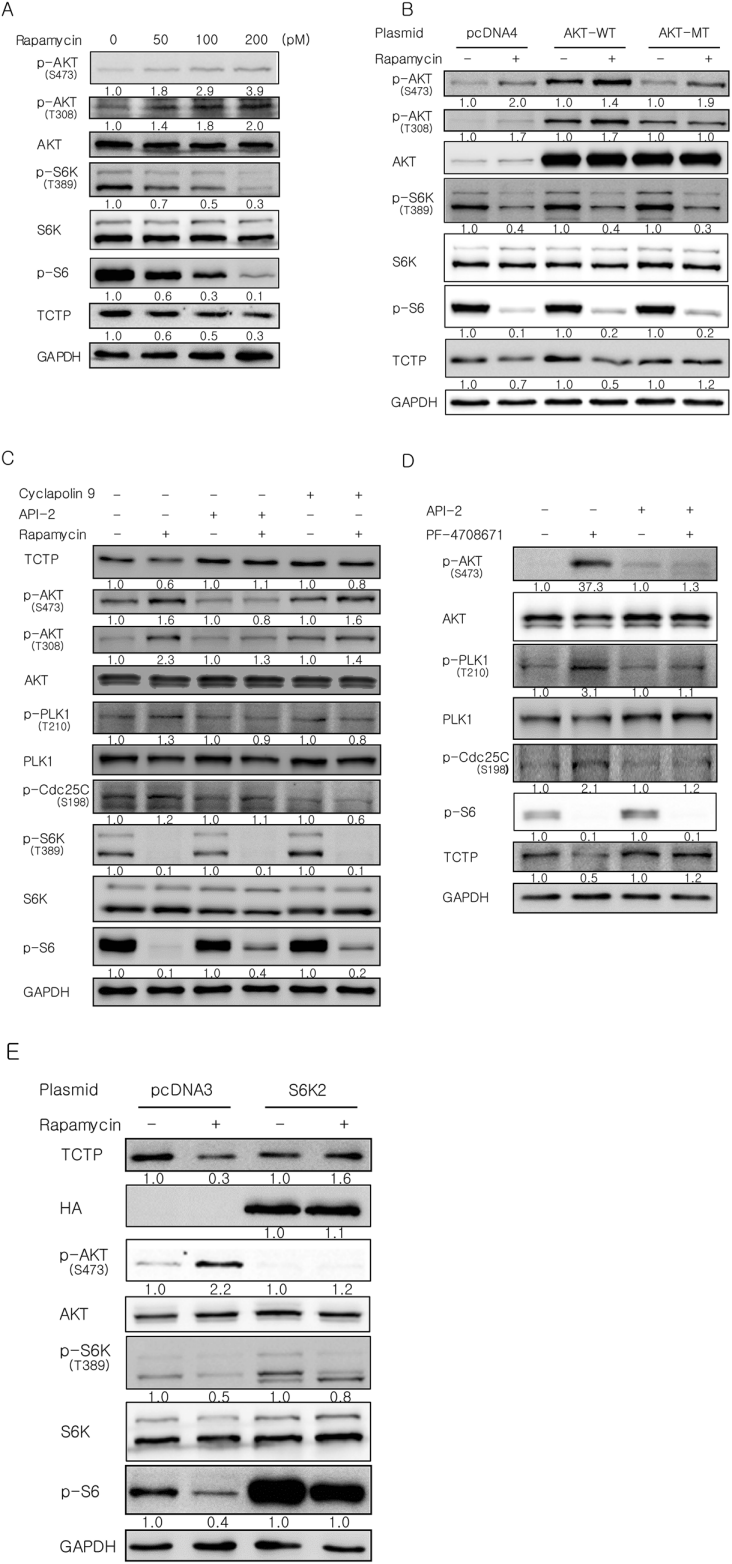
Akt activation following S6K inhibition is required for rapamycin-induced TCTP degradation through PLK1 activation. (**A**) Akt phosphorylation increased in A549 cells treated with rapamycin for 24 h. (**B**) Inhibition of Akt activation by overexpressing a non-phosphorylatable mutant of Akt1 containing both Thr308Ala and Ser473Ala mutations prevented the decrease of TCTP protein level by rapamycin. A549 cells were transfected with 5.5 μg of plasmids expressing wild-type (WT) or phospho-mutants (MT) of Akt1 or empty vector (pcDNA4) and then incubated with 100 pM rapamycin for 24 h. (**C**) Akt inhibition abolished rapamycin-induced TCTP degradation and the phosphorylation of both Akt and PLK1. A549 cells were treated with 100 pM rapamycin for 24 h after 2-h pretreatment with 5 µM cyclapolin 9 or 5 µM API-2, an Akt inhibitor. (**D**) Inhibition of S6K1 activity decreased the level of TCTP protein and induced the phosphorylation of Akt and PLK1. A549 cells were treated with 10 µM PF-4708671, a S6K1 inhibitor, for 24 h after a 2-h pretreatment with 5 µM API-2. (**E**) Overexpression of constitutively active S6K2 inhibited TCTP degradation and Akt activation by rapamycin. A549 cells transfected with 5.5 μg of plasmid were treated with 100 pM rapamycin for 24 h. Total protein was analyzed by immunoblotting. The band intensities were quantified and normalized to those of internal controls or the total forms, and fold changes compared with controls are presented as numbers below the bands. GAPDH and α-tubulin proteins were used as internal controls. Triplicate experiments were performed.

mTOR inhibition by rapamycin induces feedback activation of Akt^29–32^ and it has been suggested that Akt may induce PLK1 activation^33,34^. Akt phosphorylation at Thr^308^ and Ser^473^ is necessary for activation of the enzyme and a non-phosphorylatable mutant of Akt1 containing both Thr308Ala and Ser473Ala mutations inhibits endogenous Akt activation^36,37^. Therefore, we evaluated changes in Akt phosphorylation at Thr^308^ and Ser^473^ in A549 cells treated with rapamycin. Akt phosphorylation at these residues increased significantly with rapamycin treatment in a dose-dependent manner (Fig. 5A). Next, we investigated whether Akt phosphorylation and activation are necessary for rapamycin-induced TCTP degradation. A549 cells were transfected with wild-type (AKT-WT) or a non-phosphorylatable mutant of Akt1 containing both Thr308Ala and Ser473Ala mutations (AKT-MT) followed by treatment with rapamycin. Overexpression of the non-phosphorylatable mutant of Akt1 completely prevented the decrease of TCTP protein caused by rapamycin treatment, whereas overexpression of WT Akt1 did not (Fig. 5B). Although Akt phosphorylation at Ser^473^ increased in the cells overexpressing the Akt mutant, phosphorylation at both Thr^308^ and Ser^473^ is necessary for Akt activation and therefore we believe that overexpression of the Akt mutant inhibited endogenous Akt activation. API-2 suppresses the kinase activity and phosphorylation of Akt without inhibiting known upstream activators of Akt, including PI3K and PDK1^38^. Therefore, we used API-2 as a selective and potent Akt inhibitor. Pretreatment with API-2 abolished rapamycin-induced TCTP degradation and the phosphorylation and activation of Akt and PLK1 (Fig. 5C, lanes 1–4), whereas PLK1 inhibition with cyclapolin 9 did not change the phosphorylation status of Akt (Fig. 5C, lanes 5 and 6). Thus, Akt is an upstream kinase of PLK1 but not vice versa. Phosphorylation of PLK1 and Cdc25C was slightly changed but the phosphorylation pattern of PLK1 was similar to that of Cdc25C. Therefore, we believe the inhibitory effect of cyclapolin 9 on PLK1 is significant.

Meanwhile, S6K activation following mTORC1 activation negatively regulates Akt activity through insulin receptor substrate (IRS) or mTORC2^29–32^. First, we explored whether rapamycin treatment induced TCTP degradation through S6K. In A549 cells, a single treatment of PF-4708671, a specific inhibitor of S6K1, also decreased the level of TCTP protein and induced the phosphorylation and activation of Akt and PLK1 (Fig. 5D, lanes 1 and 2). Furthermore, inhibition of Akt with API-2 abrogated the decrease in TCTP protein levels and PLK1 activation by inhibiting S6K (Fig. 5D, lanes 3 and 4). Next, we examined whether rapamycin treatment depends on mTORC2 to induce Akt activation following S6K inhibition. Overexpression of constitutively active S6K2 inhibited TCTP degradation and the activation of Akt by rapamycin (Fig. 5E), whereas the inhibition of mTORC2 activity by Rictor siRNA transfection had no effect (Supplementary Fig. S2A). In contrast, we observed, similar to previous reports^39,40^, that inhibition of mTORC2 activity by Rictor siRNA transfection completely blocked mTORC2-dependent Akt phosphorylation at Ser^473^ following insulin treatment (Supplementary Fig. S2B). This indicates that the siRNA effectively suppressed mTORC2 activity. Based on these results, Akt activation by rapamycin depends on S6K inhibition, but not mTORC2 activation, suggesting that Akt is activated by rapamycin, probably through S6K-IRS signaling. Collectively, these results demonstrate that Akt activation following S6K inhibition, in which mTORC2 is not involved, plays a key role in PLK1 activation and TCTP protein degradation upon mTORC1 inhibition.

### Rapamycin sensitizes lung cancer cells to DNA-damaging agents in vitro and in vivo through TCTP down-regulation and enhanced apoptosis

**Figure 6:**
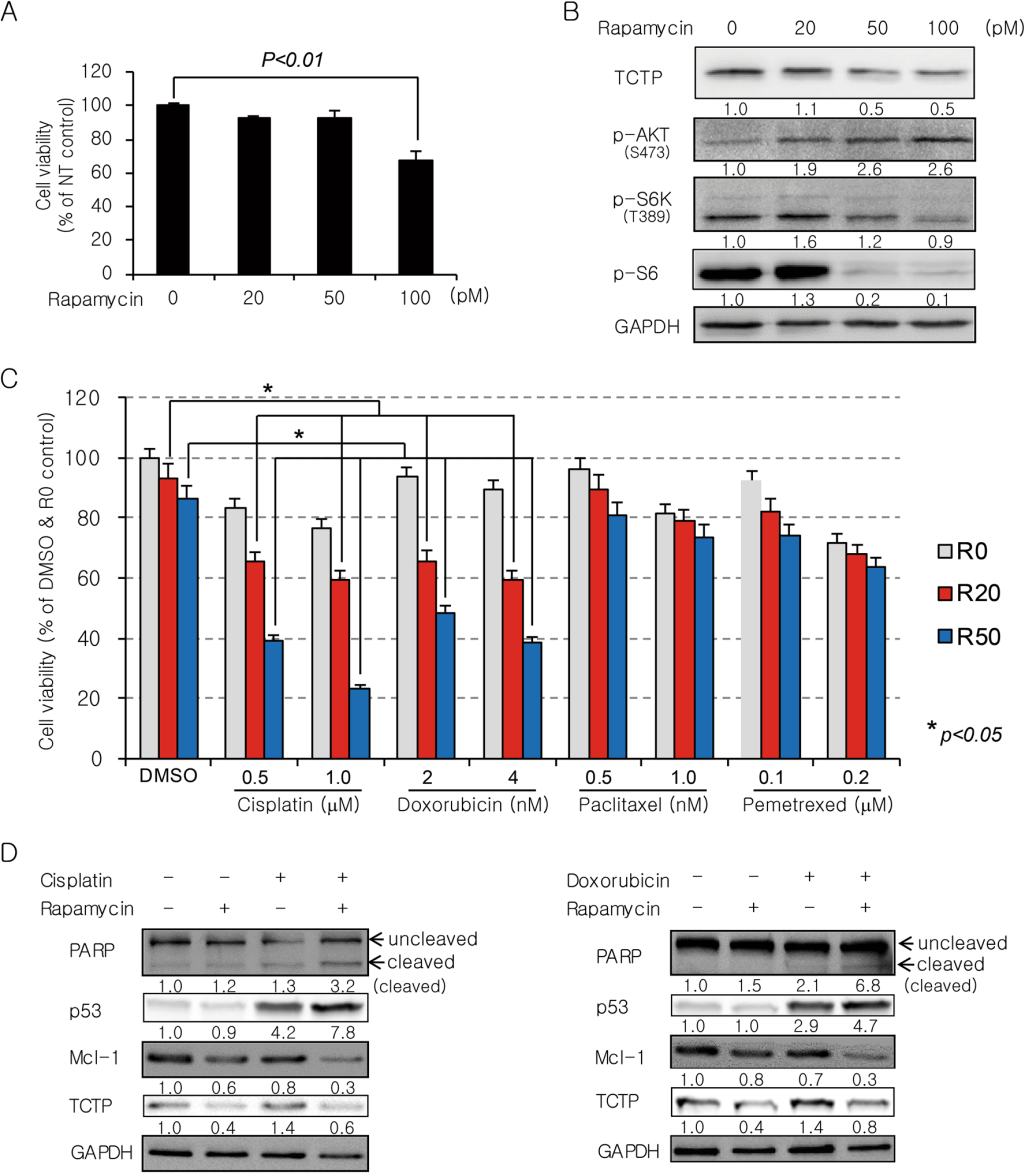

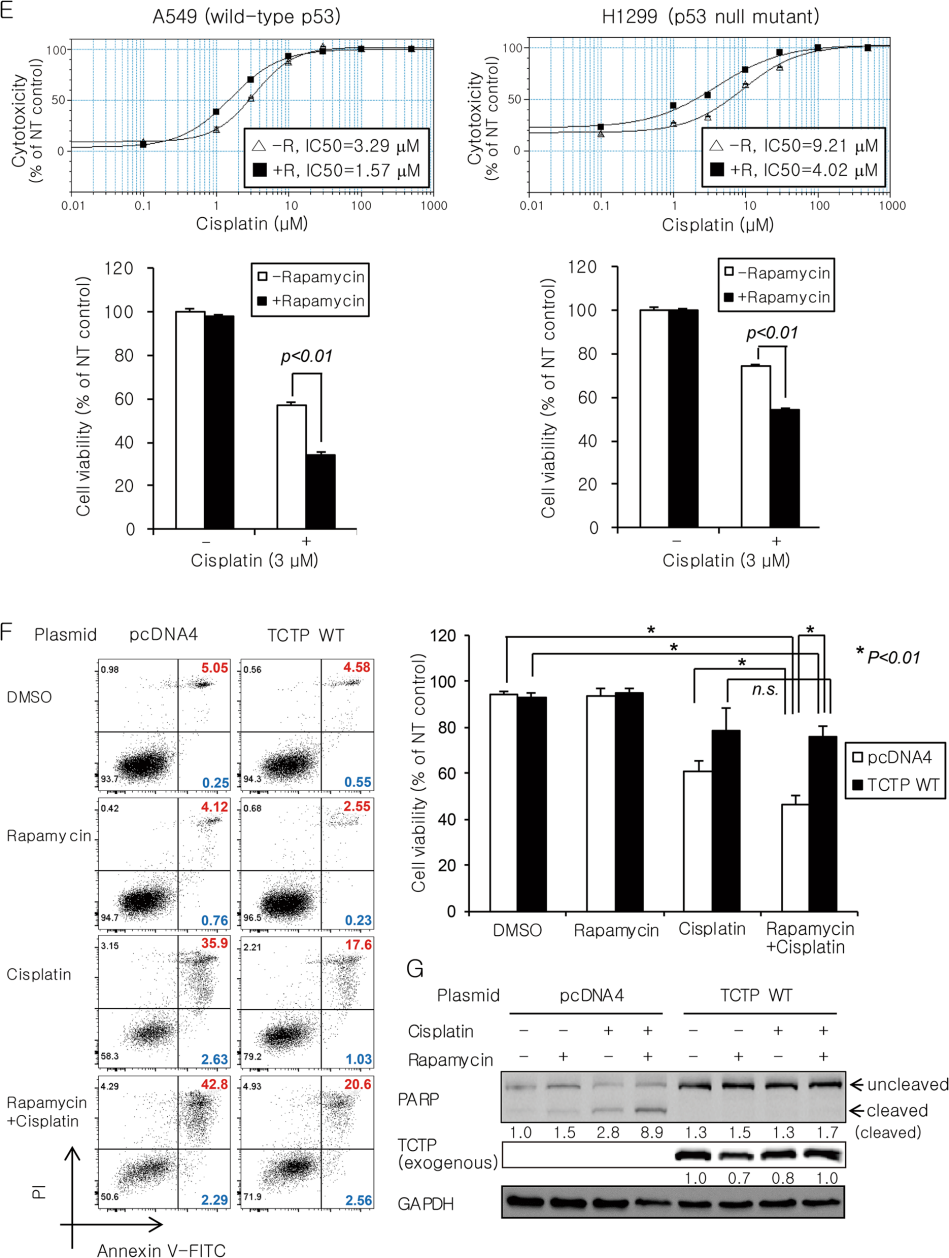
Rapamycin sensitizes lung cancer cells to DNA-damaging agents by reducing TCTP protein levels and promoting apoptosis. (**A**,**B**) A 4-day treatment with rapamycin had a minimal effect on the viability of A549 cells despite a significant decrease in mTORC1 activity (evidenced by a marked decrease of p-S6). (**C**) Rapamycin enhanced the cytotoxicity of the DNA-damaging drugs, cisplatin and doxorubicin. A549 cells were pretreated with 0, 20, or 50 pM rapamycin (R0, R20, or R50) for 2 h and then incubated with various chemotherapeutic drugs for 4 days. (**D**) Rapamycin enhanced the effect of cisplatin and doxorubicin on cleavage of PARP protein and levels of p53 and Mcl-1. A549 cells were treated with 5 µM cisplatin or 10 nM doxorubicin in the presence or absence of 200 pM rapamycin for 2 days. (**E**) Rapamycin enhanced the sensitivity to cisplatin to a similar extent irrespective of the functional status of p53. Cells were incubated for 3 days with various concentrations of cisplatin in the presence (+ R) or absence (− R) of 200 pM rapamycin (upper panel) or co-treated with 200 pM rapamycin and 3 μM cisplatin (lower panel). (**F**,**G**) Overexpression of wild-type TCTP diminished the synergistic effect of rapamycin and cisplatin on apoptosis (**F**) and cleavage of PARP protein (**G**). A549 cells were transfected with 5.5 μg of plasmid expressing wild-type TCTP or empty vector (pcDNA4) for 24 h and then treated with 100 pM rapamycin and/or 5 µM cisplatin for 3 days. Cell viability was determined using the cell counting kit-8 (CCK-8) assay and IC50 values were calculated using the software accompanying the SpectraMax microplate reader (**A**,**C**,**E**). Cell lysates were analyzed by immunoblotting (**B**,**D**,**G**). Cells were stained with FITC-conjugated annexin V antibody and PI for 30 min and the percentage of early and late apoptotic cells was determined by flow cytometry and indicated by blue and red colors, respectively (**F**). The band intensities were quantified and normalized to those of internal controls or the total forms, and fold changes compared with controls are presented as numbers below the bands. GAPDH protein was used as an internal control. DMSO (≤ 0.1%) was used as a vehicle control and NT indicates no treatment. Triplicate experiments were performed.

Recently, it has been reported that TCTP plays an important role in double-strand break (DSB) repair through both non-homologous end joining (NHEJ) and homologous recombination (HR)^41–43^. To determine the manner in which TCTP degradation by mTORC1 inhibition influences the efficacy of anticancer drugs, especially DNA-damaging agents, we examined whether rapamycin sensitizes A549 cells to different classes of anticancer drugs. A 4-day treatment with 100 pM rapamycin alone had a minimal effect on the viability of A549 cells, even though it was sufficient to remarkably reduce mTORC1 activity (evidenced by a decrease of p-S6K and p-S6) and TCTP protein levels and significantly activate Akt (Fig. 6A,B). Next, A549 cells were treated with sublethal concentrations of cisplatin (DNA cross-linking agent), doxorubicin (DNA-intercalating agent), paclitaxel (mitotic inhibitor), or pemetrexed (antifolate) in the presence or absence of 20 or 50 pM rapamycin (Fig. 6C). Interestingly, the minimal dose of rapamycin significantly (*P* < 0.05) and synergistically increased the cytotoxicity of the DNA-damaging drugs, cisplatin and doxorubicin, but not paclitaxel and pemetrexed. At the protein level, we observed that co-treatment with rapamycin not only increased p53 and cleaved PARP, but also decreased Mcl-1 (Fig. 6D, lanes 4 vs. lanes 3 in both panels). Moreover, TCTP knockdown by siRNA transfection also enhanced p53 induction by cisplatin treatment (Supplementary Fig. S3A). TCTP promotes cell survival by regulating the stability of p53 and Mcl-1 and Bax dimerization^7,13–17^. Therefore, we hypothesized that the efficacy of DNA-damaging agents can be enhanced by rapamycin irrespective of p53 mutation status because of the multimodality of TCTP’s anti-apoptotic activity. To determine how the functional status of p53 affects the synergistic cytotoxicity of rapamycin and cisplatin, we examined whether rapamycin sensitizes both WT and null p53 lung cancer cells (A549 and H1299) to cisplatin. Half-maximal inhibitory concentration (IC50) values of cisplatin decreased 2.1- and 2.3-fold in A549 and H1299 cells in the presence of rapamycin, respectively (Fig. 6E, upper panel). In both cell lines, rapamycin significantly (*P* < 0.01) and synergistically increased the cytotoxicity of 3 μM cisplatin (Fig. 6E, lower panel). Despite the lack of p53 protein expression and induction in H1299 cells, co-treatment with rapamycin not only increased the cleaved form of PARP, but also decreased Mcl-1 (Supplementary Fig. S3B). Co-treatment with rapamycin also synergistically increased the pro-apoptotic activity of cisplatin (Fig. 6F, left column in the left panel and white bar in the right panel). In contrast, overexpression of WT TCTP markedly diminished the synergistic effect of rapamycin and cisplatin on apoptosis (Fig. 6F, right column in the left panel and black bar in the right panel) and abolished the change in level of cleaved PARP (Fig. 6G). A similar result was observed in A549 cells co-treated with rapamycin and doxorubicin (Supplementary Fig. S3C). The overexpression and knockdown of TCTP were confirmed using qRT-PCR (Supplementary Fig. S3D).

**Figure 7:**
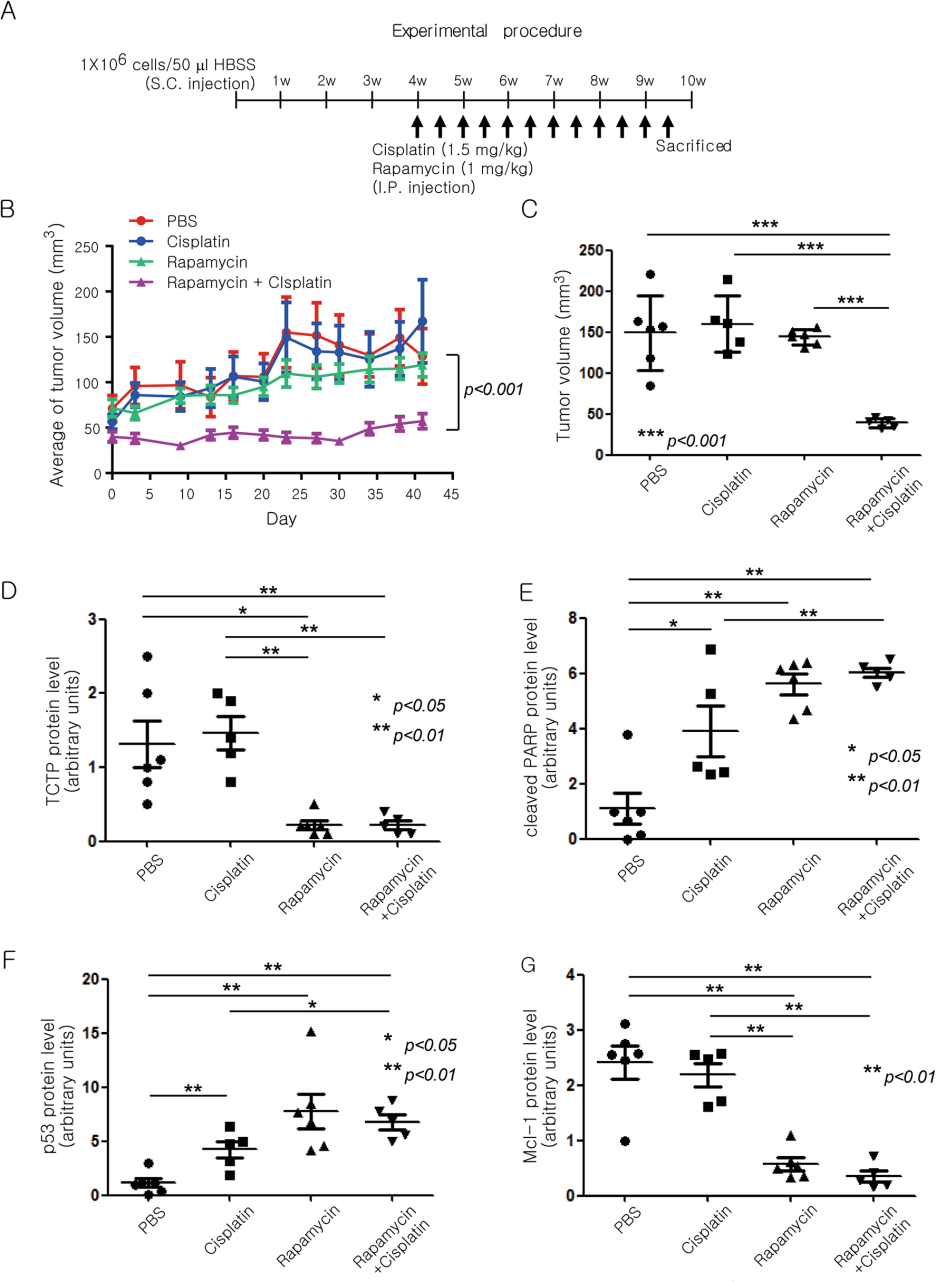

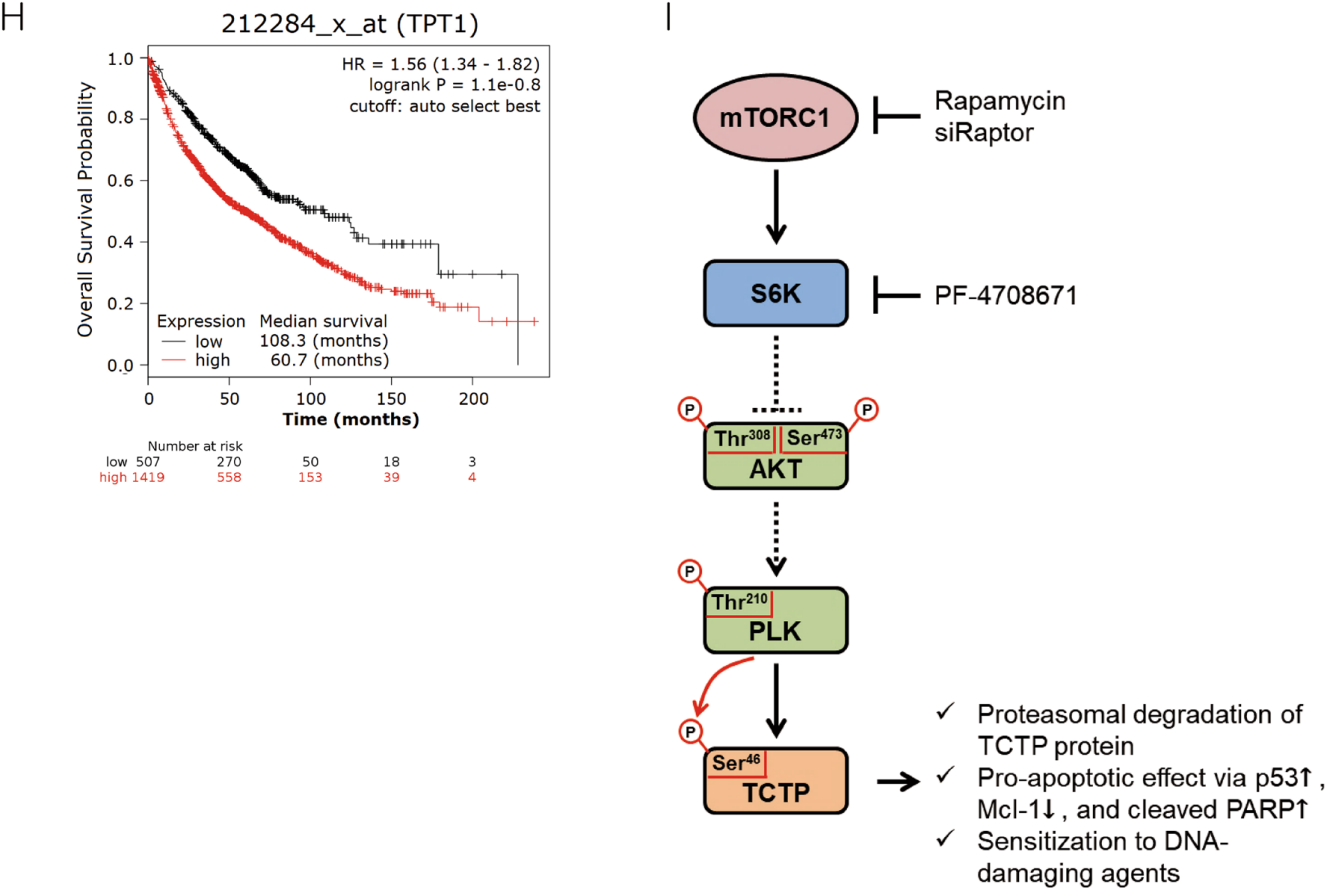
Rapamycin enhances the efficacy of cisplatin in an A549 lung cancer xenograft model through TCTP down-regulation and apoptosis promotion. (**A**–**G**) A549 cells were inoculated into BALB/c nude mice. After 4 weeks, mice were injected with 1.5 mg/kg cisplatin and/or 1 mg/kg rapamycin twice a week for 6 weeks. The long and short diameters of each tumor and the body weight of the mice were measured twice a week. (**A**) Schematic of the experimental procedure is depicted. (**B**,**C**) Changes in the within-group average of tumor volume (mm^3^) at each measurement point and the tumor volumes at the end point are presented as means ± SD. (**D**–**G**) Using the immunodetection data in Supplementary Fig. S4C, the protein levels of TCTP (**D**), cleaved PARP (**E**), p53 (**F**), and Mcl-1 (**G**) were quantified, normalized to that of GAPDH or total PARP, and are presented as means ± SD. (**H**) Overall survival of lung cancer patients with high TCTP expression was significantly lower compared with that of patients with low TCTP expression (*P* = 1.1 × 10^−8^). Kaplan–Meier curves for overall survival in lung cancer patients according to the expression of TPT1 probe 212284_x_at (n = 1926) were obtained from the databases in Kaplan–Meier plotter (http://kmplot.com/analysis/). The log-rank test was performed using auto select best value as a cutoff. (**I**) The schematic illustrates a novel signaling pathway through which TCTP protein is maintained at a high level in lung cancer cells along with the biochemical, molecular, and biological effects of mTORC1 inhibition through TCTP down-regulation.

To confirm whether combined treatment with rapamycin improved the efficacy of cisplatin in an in vivo tumor model, we performed a xenograft experiment. A549 cells (1 × 10^6^) were injected subcutaneously into the lower flanks of BALB/c nude mice. After 4 weeks, when the average size of the tumors reached 100 mm^3^, 1 mg/kg rapamycin and/or 1.5 mg/kg cisplatin were injected intraperitoneally twice a week for 6 weeks (Fig. 7A). To monitor the toxicity and the side effects of the drugs, we measured body weight of the mice as an indicator of general health conditions. The change in body weight was similar among the four groups (PBS control, rapamycin, cisplatin, rapamycin and cisplatin) (Supplementary Fig. S4A). Co-treatment with rapamycin and cisplatin delayed tumor growth significantly longer (*P* < 0.001) compared with treatment with either rapamycin or cisplatin alone (Fig. 7B,C, and Supplementary Fig. S4B). Protein levels of TCTP, PARP, p53, Mcl-1, phosphor-Akt, Akt, phosphor-S6K, S6K, and phospho-S6 were analyzed in all tumor tissues (Supplementary Fig. S4C). At the protein level, the rapamycin dosage alone was sufficient to cause a decrease in TCTP and Mcl-1 and an increase in p53 and cleaved PARP (Fig. 7D–G, and Supplementary Fig. S4C). As expected, TCTP protein levels in the tumor tissues decreased significantly (*P* < 0.01 or *P* < 0.05) in both rapamycin-treated groups. Mcl-1 protein levels also decreased and the protein levels of p53 and cleaved PARP increased in both rapamycin-treated groups. Moreover, S6K inhibition and Akt activation were observed in the rapamycin-treated groups (Supplementary Fig. S4D–F). Thus, rapamycin enhanced the anticancer effect of cisplatin by reducing cellular TCTP levels and inducing apoptotic cell death in xenograft tumors.

To investigate the relationship between patient survival and TCTP expression in human lung cancer, we performed a survival analysis using databases in the Kaplan–Meier plotter (http://kmplot.com/analysis/). The group of patients with high TCTP expression showed worse overall survival compared with those with low TCTP expression (Fig. 7H).

## Discussion

TCTP levels change rapidly under various cellular conditions which suggests that the expression of TCTP is highly regulated^2–4^. In this study, we discovered a novel signaling pathway, which is schematically illustrated in Fig. 7I. We provided several lines of evidence to indicate that mTORC1/S6K/Akt/PLK1 signaling is indispensable for maintaining a high level of TCTP protein in lung cancer cells. First, mTORC1 was involved in the regulation of the level of TCTP protein. In various lung cancer cells, the level of TCTP protein was markedly reduced by the inhibition of mTORC1 activity, but not mTORC2 activity (Fig. 1A–D). Second, the reduction of cellular TCTP levels upon mTORC1 inhibition resulted, in part, from increased protein degradation through the ubiquitin–proteasome system (Figs. 2A–D and 3E). Third, and most importantly, PLK1 phosphorylation and activation were required for TCTP degradation by mTORC1 inhibition. When PLK1 activity or expression was suppressed, the degradation and ubiquitination of TCTP protein was almost completely inhibited (Fig. 3B–E). These findings demonstrate that mTORC1 negatively regulates PLK1. To our knowledge, this is the first report describing the negative regulation of PLK1 by mTORC1. The functional link between mTORC1 and PLK1 is currently controversial. PLK1 phosphorylates TSC1 and functions as a positive upstream regulator of mTORC1^44,45^, whereas it phosphorylates Raptor, inhibits mTORC1, and thereby induces autophagy in interphase cancer cells^46^. Fourth, upon mTORC1 inhibition, TCTP phosphorylation at Ser^46^ was entirely prevented by the inhibition of PLK1 kinase activity (Fig. 4D,E), indicating that PLK1 phosphorylated TCTP at Ser^46^ when mTORC1 was inhibited. In support of this notion, mTORC1 inhibition resulted in increased phosphorylation of PLK1 at Thr^210^ and of Cdc25C at Ser^198^, which indicates PLK1 activation (Figs. 4C and 5C, lanes 1 and 2). Fifth, phosphorylation mutants of TCTP, including a Ser46Ala mutation, were refractory to rapamycin-induced degradation (Fig. 4F). Sixth, the link between mTORC1 inhibition and PLK1 activation was mediated by the S6K-Akt signaling pathway. Rapamycin treatment induced Akt phosphorylation (Fig. 5A,B,C,E, lanes 1 and 2) and overexpression of a phosphorylation mutant of Akt prevented rapamycin-induced TCTP protein degradation (Fig. 5B). Rapamycin-induced phosphorylation and activation of both Akt and PLK1 was completely blocked by selective inhibition of Akt, but not PLK1 (Fig. 5C). Moreover, the specific inhibition of S6K1 alone resulted in a decrease in TCTP protein and phosphorylation and activation of Akt and PLK1, both of which were abrogated by selective inhibition of Akt (Fig. 5D). The overexpression of constitutively active S6K2 abolished Akt activation and TCTP protein degradation upon rapamycin treatment (Fig. 5E). To our knowledge, this is also the first experimental report describing the positive regulation of PLK1 by Akt and the ubiquitin-proteasomal degradation of protein phosphorylated by PLK1.

Translational regulation of TCTP expression is well documented^3,4^. It has been demonstrated in human cancer cells as well as mouse skeletal muscle and Xenopus laevis embryos that PI3K/Akt/mTORC1 signaling plays a role in regulating TCTP translation^20–22,47,48^. Although the rapamycin-induced decrease in TCTP protein was still remarkable following pretreatment with cycloheximide, an inhibitor of protein synthesis (Fig. 2D), this finding does not rule out the possibility that TCTP mRNA translation is also inhibited by rapamycin. Therefore, it is possible that mTORC1 inhibition decreases TCTP protein levels through the combined effect of accelerated TCTP protein degradation and inhibition of TCTP mRNA translation. Little is known regarding the regulation of TCTP protein degradation. Mcl-1, heat shock protein 27, and dihydroartemisinin, an anti-malaria drug, bind directly to TCTP and regulate its stability^49–51^. TCTP degradation is dependent upon ubiquitination and/or proteasome^51,52^ and chaperone-mediated autophagy (CMA)^53^. In addition, mTORC1 inhibition increased the degradation of long-lived cellular proteins by the ubiquitin–proteasome system as well as by autophagy, which does not depend on S6Ks and Akt in contrast to our findings^54^. Macroautophagy, which is inhibited by mTORC1, is a well-known protein degradation pathway. Our findings that a reduction of TCTP protein caused by mTORC1 inhibition was completely prevented following pretreatment with various proteasome inhibitors (Fig. 2A,B) indicates that the decrease in TCTP levels by mTORC1 inhibition depends upon proteasomal degradation. However, further investigation is required to rule out the possibility that TCTP protein degradation by mTORC1 inhibition depends, at least in part, on CMA or macroautophagy. Meanwhile, both Mcl-1 and TCTP were down-regulated following rapamycin treatment (Figs. 6D, 7G). There is a reciprocal stabilization between TCTP and Mcl-1^16,49^. In a murine lymphoma model, blockade of mTORC1 by rapamycin downregulated Mcl-1 translation and resulted in rapid apoptosis^55^. Therefore, it is possible that rapamycin down-regulates Mcl-1 expression and thereby decreases TCTP protein stability or co-downregulates both TCTP and Mcl-1. Down-regulation of Mcl-1 expression by rapamycin was abolished following pretreatment with a PLK1 inhibitor, although mTORC1 activity was still inhibited (Supplementary Fig. S4G). Our finding suggests that rapamycin treatment results in TCTP protein degradation and thereby Mcl-1 destabilization. However, a single treatment of PLK inhibitor already reduced Mcl-1 as much as rapamycin did and therefore it is possible that pretreatment with a PLK1 inhibitor abolished down-regulation of Mcl-1 expression by rapamycin. Thus, more in-depth studies are necessary to resolve this issue.

TCTP leads to cell survival by regulating the stability of p53 and Mcl-1 and Bax dimerization^7,13–17^ and it contributes to DSB repair through the NHEJ and HR pathways^41,42^. Therefore, we investigated the biological significance of mTORC1-induced TCTP expression in terms of resistance to anticancer drugs, particularly DNA-damaging agents. As expected, a reduction of TCTP protein levels by rapamycin enhanced the cytotoxicity of DNA-damaging drugs (cisplatin and doxorubicin) (Figs. 6C, 7B–D, and Supplementary Fig. S4B) and was accompanied by changes in the levels of cleaved PARP, p53, and Mcl-1 protein, which induced apoptosis (Fig. 6D,F, 7E–G, and Supplementary Fig. S4C). Importantly, we also demonstrated that the efficacy of DNA-damaging agents can be enhanced by an mTORC1 inhibitor (e.g., rapamycin) regardless of the functional status of p53, the gene most frequently mutated among all the genes examined to date in human cancer (Fig. 6E and Supplementary Fig. S3B). In the present study, we found that the efficacy of cisplatin was enhanced by a low dose of rapamycin (20–200 pM), unlike most previous reports that used high concentrations of rapamycin (1–100 nM) in various cancer cells^56–61^. Further studies on the biological role of mTORC1/S6K/Akt/PLK1 signaling in the control of cellular TCTP levels will improve our understanding of TCTP function in tumor maintenance and carcinogenesis considering its established roles in malignant transformation^8,9^, tumor reversion^2,10,12,13^, anti-apoptotic function^13–17^, and DNA damage sensing and repair^41–43^. Furthermore, the biological significance of mTORC1-induced TCTP expression may be considered as follows: the beneficial effects of mTORC1 activation on tumor growth and progression may result, at least in part, from TCTP upregulation and thereby suppression of p53 function. mTORC1 signaling is activated in a large proportion of human cancers^62^ and plays an important role in tumorigenesis and tumor maintenance^63^. Even though no mutation in mTOR itself has yet been reported, gain-of-function mutations in various components of the PI3K signaling pathway upstream of mTOR occur frequently in human cancers^63–65^. The present study demonstrated that mTORC1 stabilized TCTP protein. Thus, mTORC1 activation commonly observed in cancers may induce the overexpression of TCTP which stimulates cell growth and proliferation and inhibits apoptosis. Meanwhile, TCTP binds directly to mdm2 and inhibits its ubiquitination and degradation, and eventually leads to increased ubiquitination and degradation of the p53 protein^13–15^. Therefore, mTORC1 may negatively regulate the level and activity of p53 by stabilizing TCTP protein. In fact, a few reports have considered the negative^66,67^ or positive^68^ regulation of p53 by mTORC1. However, whether mTORC1 suppresses p53 function via upregulation of TCTP remains unknown. In the present study, the induction of p53 protein after treatment with cisplatin or doxorubicin was enhanced upon mTORC1 inhibition by rapamycin (Fig. 6D, lanes 4 vs. lanes 3 in both panels) and TCTP knockdown by siRNA transfection (Supplementary Fig. S3A). This suggests that mTORC1 upregulates TCTP protein and thereby negatively regulates p53 function.

PLK1 is maximally activated at the G_2_/M phase of the cell cycle^24,28^. It is well-known that rapamycin induces cell cycle arrest at the G1 phase, although the sensitivity to rapamycin-induced cell cycle arrest depends on the cancer cell line studied^69–71^. Thus, it is necessary to rule out the possibility that rapamycin induces G_2_/M phase arrest and that rapamycin-induced PLK1 activation is a simple consequence of that G_2_/M arrest. In this regard, we confirmed that treatment with 100 pM rapamycin, a concentration adequate to reduce the level of TCTP protein, had little effect on cell cycle progression, whereas treatment with 100 nM and 20 μM rapamycin induced cell cycle arrest at the G_1_ phase, but not at G_2_/M phase as described in other reports (Supplementary Fig. S5). These results indicate that PLK1 activation induced by rapamycin did not result from G_2_/M phase arrest.

In summary, we have provided insight into a novel signaling mechanism of TCTP regulation at the post-translational level in which the mTORC1/S6K signaling pathway negatively regulates a novel Akt/PLK1 axis and maintains TCTP at high levels in lung cancer cells. This mechanism provides a new therapeutic strategy for cancer patients with high expression of TCTP: the administration of mTORC1 inhibitors in combination with DNA-damaging agents to enhance efficacy and enabling a reduction in dosage and side effects.

## Methods

### Chemicals

Rapamycin, PP242, MG-132, ALLN, CLBL, cycloheximide, insulin, and pemetrexed were purchased from Calbiochem. Cyclapolin 9 and API-2 were obtained from Tocris. PF-4708671, cisplatin, doxorubicin, and paclitaxel were obtained from Selleckchem. The cell counting kit-8 (CCK-8) was purchased from Dojindo Molecular Tech. All other chemicals were purchased from Sigma-Aldrich.

### Plasmid construction

Expression plasmids encoding WT and phospho-mutants of TCTP were constructed using PCR. A 519-bp PCR-amplified fragment of the WT *TCTP* gene (NM_003295.2) was generated using a cDNA library from A549 cells and the following primers: forward primer containing a *Bam*HI site, 5′-GGATCCATGATTATCTACCGGGAC-3′ and reverse primer containing an *Xho*I site, 5′-CTCCAGTTAACATTTTTCCATTTCT-3′. The *Bam*HI and *Xho*I restriction fragments were subcloned into the pGEM-T vector (Promega). To produce phospho-mutants of TCTP, serine residues 46 and 64 were substituted with alanine and threonine residue 65 was substituted with valine. For PCR-mediated site directed mutagenesis, the forward and reverse primers were the same as in the construction of the WT TCTP plasmid. The primers used to introduce point mutations are listed in Supplementary Table S1. All primers were purchased from Bioneer (Korea). All constructs were verified by DNA sequencing. To construct mammalian expression plasmids, DNA fragments encoding WT and phospho-mutants of TCTP were subcloned from the pGEM-T vector into a pcDNA4/HisMax C vector containing a His-tag. Plasmids expressing WT (#9003) and phospho-mutant (T308A and S473A, #9030) Akt1 and constitutively active S6K2 with hemagglutinin (HA) tag (E388 and D3E, #17731) were obtained from Addgene.

### Cell culture and transfection

Human lung cancer cells (A549, H1299, and H157) were cultured in RPMI 1640 medium (Hyclone) supplemented with l-glutamine (Gibco), sodium pyruvate (Gibco), 10% fetal bovine serum (FBS) (Hyclone), and antibiotics (Gibco). The human lung carcinogenesis model cell lines (BEAS-2B, 1799, 1198, and 1170-I cells^72^) were cultivated as previously described^73^. Briefly, the BEAS-2B and 1799 cells were grown in Keratinocyte serum-free medium (Gibco) supplemented with epidermal growth factor (Gibco) and pituitary extract (Gibco), and the 1198 and 1170-I cells were cultured in the same medium except that 3% FBS (Gibco) was added. The cells were incubated at 37 °C in a humidified atmosphere containing 5% CO_2_. A549 cells were purchased from the ATCC and H1299, H157, BEAS-2B, 1799, 1198, and 1170-I cells were a generous gift from Dr. Y. H. Kim (Korea Univ., Seoul). The cells were cultured mycoplasma-free for a period of less than 4 months after thawing. The cell lines were authenticated regularly by morphologic observation, and frozen stocks of A549, H1299, H157, BEAS-2B, 1799, 1198, and 1170-I were authenticated at University of Arizona Genetics Core and Macrogen (Korea) using short tandem repeat DNA fingerprinting on Jan 8, 2018 and Aug 20, 2019.

For gene silencing of Raptor (NM_020761.2), Rictor (NM_001285439.2), PLK1 (NM_005030.3), and TCTP (NM_003295.2) or the overexpression of WT and mutant Akt1, S6K2, and TCTP, cells were transiently transfected with siRNAs (Bioneer, Korea) or expression plasmids, respectively, using Lipofectamine 2000 (Invitrogen). Cells were collected at 36–48 h after transfection for immunoblotting. Green fluorescent protein (GFP) siRNA (SP-2003) and *AccuTarget* Negative Control siRNA (SN-1003) were used as negative controls. The siRNA sequences are listed in Supplementary Table S1.

### Immunoblotting

Cells were lysed for 30 min in cold lysis buffer (20 mM Tris pH 7.5, 100 mM NaCl, 5 mM MgCl_2_, 1% NP-40, and 0.5% sodium deoxycholate) supplemented with protease (Roche) and phosphatase (Calbiochem) inhibitors. Tumor tissues collected from mice were homogenized in 300–500 μl (five volumes) of cold lysis buffer. Equal amounts of protein (20–30 μg) were resolved by 10% SDS-PAGE, transferred to nitrocellulose membranes (Whatman), and detected with antibodies against TCTP (Abcam), phospho-TCTP-Ser46, Raptor, Rictor, S6K, phospho-S6K-T389, PLK1, phospho-PLK1-Thr210, cell division cycle 25C (Cdc25C), phospho-Cdc25C-Ser198, Akt, phospho-Akt-T308, phospho-Akt-S473 (Cell Signaling Technology), phospho-S6 (eBioscience), ubiquitin, HA, glyceraldehyde 3-phosphate dehydrogenase (GAPDH), Mcl-1, poly(ADP-Ribose) polymerase (PARP), p53 (Santa Cruz Biotech), and β-actin and α-tubulin (AB Frontier, Korea). The antibody information is listed in Supplementary Table S1. To analyze the levels of multiple proteins separated on a single gel, a gel or a membrane was cut into pieces and then the membrane strips were hybridized with antibodies. Immunodetection was carried out with an enhanced chemiluminescent (ECL) kit (Perkin Elmer). Immunoreactive bands were visualized by X-ray film exposure or using Microchemi (DNR, Israel) and quantified using image analysis software (Multi-gauge v3.0, Fujifilm).

### Immunoprecipitation

Cells were lysed as described above for immunoblotting. Equal amounts of protein (500 μg) were pre-cleared using protein A magnetic beads (Millipore) for 2 h. The pre-cleared protein extracts were incubated for 20 min with antibodies against TCTP (Abcam), phosphoserine (Calbiochem), or normal rabbit IgG (as a negative control, Santa Cruz Biotech) and subsequently with protein A magnetic beads overnight in a rotating mixer. The immunoprecipitated proteins were analyzed by SDS-PAGE and immunoblotting.

### Reverse transcription and quantitative real time (qRT)-PCR

Total cellular RNA was prepared using Trizol reagent (Molecular Research Center) according to the manufacturer’s protocol. cDNA was synthesized at 37 °C for 1 h using Moloney murine leukemia virus reverse transcriptase, dNTPs, and oligo (dT) primer. qRT-PCR was performed using a QuantiNova SYBR Green PCR Kit (Qiagen) according to the manufacturer’s protocol. Amplification was carried out by two-step cycling. Primer sequences for the qRT-PCR methods are listed in Supplementary Table S1. All primers were purchased from Bioneer (Korea).

### Annexin V/propidium iodide (PI) double-staining assay

Apoptosis was measured by flow cytometry using annexin V/PI double staining. A549 cells were transfected with TCTP WT or pcDNA4 for 24 h and then treated with 100 pM rapamycin and/or 5 µM cisplatin for 3 days. Floating and attached cells were collected, washed twice with ice-cold PBS, and resuspended in 100 µl 1× binding buffer containing fluorescein (FITC)-conjugated annexin V antibody (1:50 dilution, according to the manufacturer’s instructions) and PI (40 ng/sample) for 30 min at 37 °C in the dark. The number of viable, apoptotic, and necrotic cells was analyzed by flow cytometry (Becton, Dickinson & Company) using FlowJo v.10 software (Becton, Dickinson & Company). At least 10,000 cells in each sample were analyzed.

### In vivo drug treatment

BALB/c nude mice were used for xenograft experiments. Five-week-old male BALB/c nude mice (Orient Bio Inc., Korea) were housed in an environmentally controlled room (22 ± 2 °C, 40–60% humidity, and a 12-h light cycle). The present study was approved by the Institutional Review Board of the Korea University College of Medicine (KOREA-2018-0016-C1). All animal procedures were performed under the Institutional Guidelines for Ethics in Animal Experiments (The Institutional Animal Care and Use Committee, Korea University College of Medicine). All animal studies were conducted in compliance with the ARRIVE guidelines^74^. A549 cells (1 × 10^6^) were inoculated subcutaneously into the flanks of the mice. When the tumor volumes reached 100 mm^3^, the mice were injected intraperitoneally with 1 mg/kg rapamycin and/or 1.5 mg/kg cisplatin twice a week for 6 weeks (n = 7 per group). The tumor size was measured twice a week. After sacrifice, tumor tissues were collected, weighed, and preserved at − 70 °C or used for immunoblotting.

### Statistical analysis

All data were statistically analyzed using Prism 5 (GraphPad Software). The data are presented with ± standard deviation (SD) values derived from at least three independent experiments. The statistical comparisons between groups were performed by an unpaired two-tailed Student’s *t* test or one-way ANOVA to calculate the *P* value for statistical significance.

## Supplementary Information

Supplementary Information.
