# Supplementary material for: TCTP protein degradation by targeting mTORC1 and signaling through S6K, Akt, and Plk1 sensitizes lung cancer cells to DNA-damaging drugs
Source: Sci Rep. 2021 Oct 21;11:20812. doi: 10.1038/s41598-021-00247-0 (PMC8531033; doi:10.1038/s41598-021-00247-0)

Supplementary Material

TCTP protein degradation by targeting mTORC1 and signaling through S6K, Akt and Plk1 sensitizes lung cancer cells to DNA-damaging drugs

Mini Jeong, Mi Hyeon Jeong, Jung Eun Kim, Serin Cho, Kyoung Jin Lee, Serkin Park, Jeongwon Sohn, Yun Gyu Park

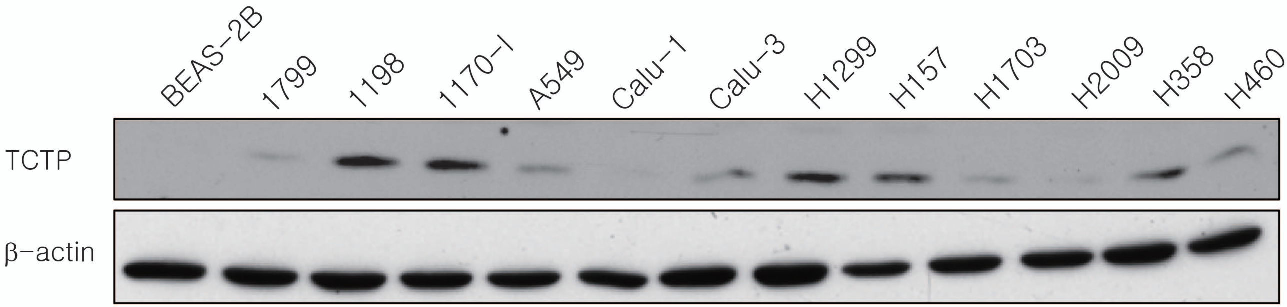

Supplementary Fig. S1. TCTP protein levels were determined in various lung cancer cells including human lung carcinogenesis model cell lines (BEAS-2B, 1799, 1198, and 1170-I cells). Total proteins were analyzed by immunoblotting. β-actin was used as an internal control. Duplicate experiments were performed.

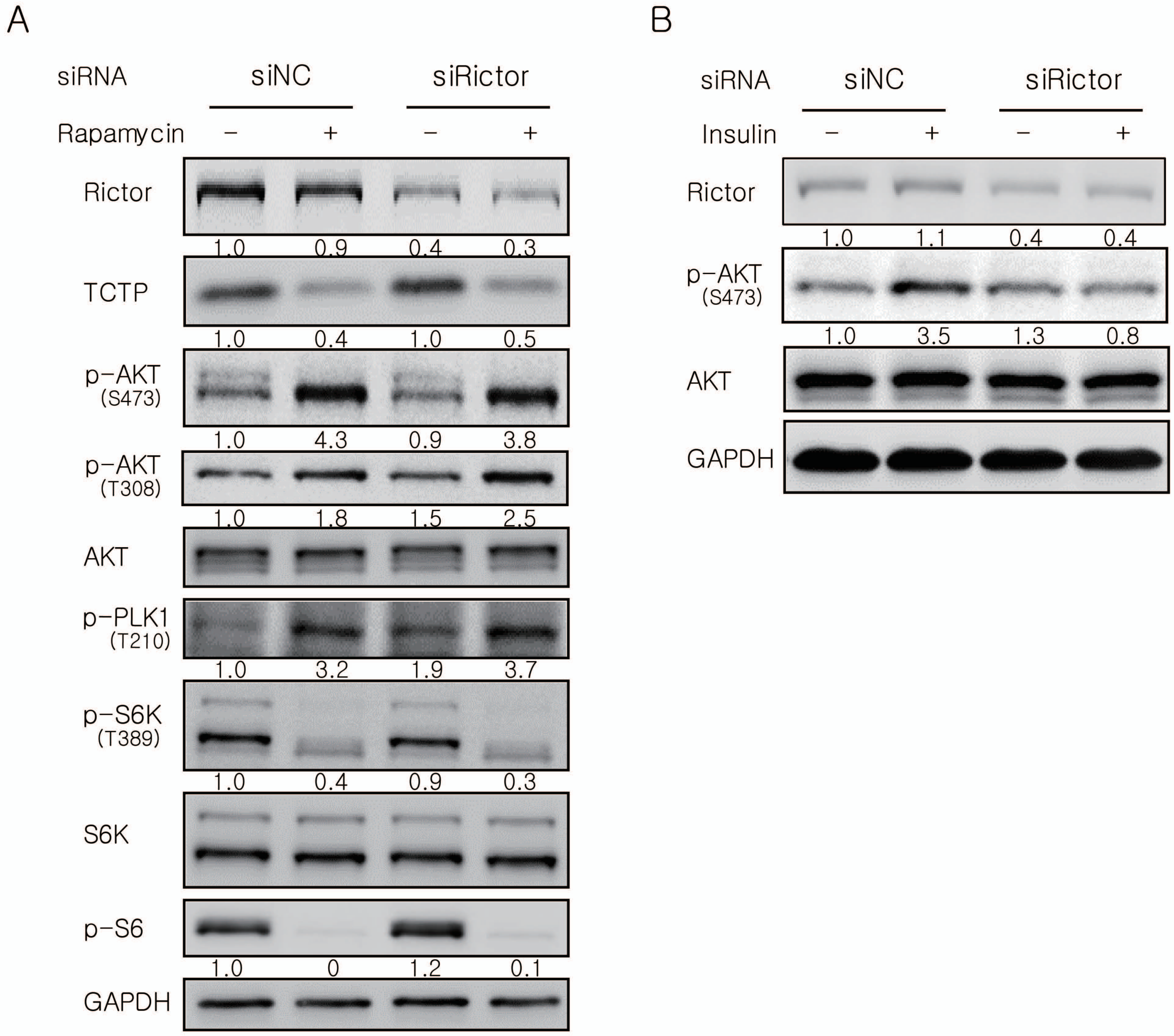

Supplementary Figures S2. mTORC2 inhibition had no effect on TCTP degradation and the activation of Akt and PLK1 by rapamycin. (A) Transfection with Rictor siRNA had no effect on TCTP degradation and the activation of Akt and PLK1 by rapamycin. (B) Rictor siRNA used in Supplementary Fig. S2A effectively suppresses mTORC2 activity. A549 cells were transfected with 100 pM siRNA for 24 hours and then treated with 100 pM rapamycin (A) or 4 μg/ml of insulin (B) for 24 hours. Cell lysates were analyzed by immunoblotting. The band intensities were quantified and normalized to those of internal controls or the total forms, and fold changes compared with controls are presented as numbers below the bands. GAPDH protein was used as an internal control. Triplicate experiments were performed.

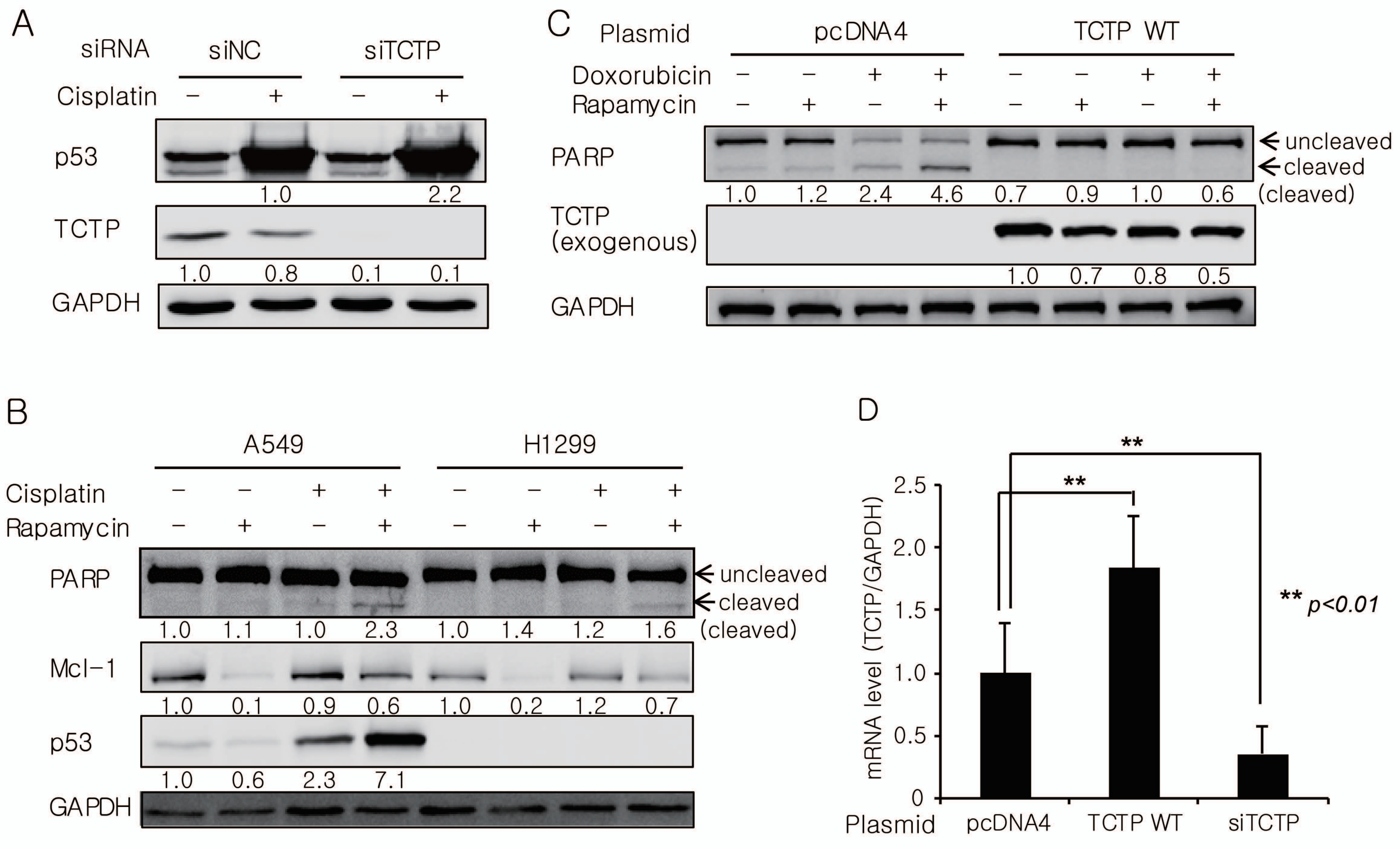

Supplementary Figure S3. TCTP is involved in cell survival against DNA-damaging agents. (A) TCTP knockdown by siRNA transfection enhanced p53 induction by cisplatin. A549 cells were transfected with TCTP siRNA or negative control siRNA at a final concentration of 100 pM for 24 hours and treated with 5 μM cisplatin for 3 days. (B) Irrespective of the functional status of p53, co-treatment with cisplatin and rapamycin increased the cleaved form of PARP and decreased Mcl-1. A549 cells were treated with 200 pM rapamycin and/or 10 μM cisplatin. H1299 cells were treated with 200 pM rapamycin and/or 30 μM cisplatin. After 24 hours, the cells were harvested and analyzed. (C) Overexpression of wild-type TCTP abolished the synergistic effect of rapamycin and doxorubicin on the change in protein level of cleaved PARP. A549 cells were transfected with 5.5 μg of a plasmid expressing wild-type TCTP or an empty vector (pcDNA4, as the negative control) for 24 hours and treated with 100 pM rapamycin and/or 4 μM doxorubicin for 3 days. (D) The overexpression and knockdown of TCTP were confirmed using qRT-PCR. A549 cells were transfected with 5.5 μg of plasmid expressing wild-type TCTP or empty vector (pcDNA4) or 100 pM TCTP siRNA for 24 hours. Total protein was analyzed by immunoblotting and GAPDH protein was used as an internal control (A, B, and C). The band intensities were quantified and normalized to those of internal controls and fold changes compared with controls are presented as numbers below the bands. Total cellular RNA was extracted and analyzed by qRT-PCR and the TCTP mRNA levels were normalized to GAPDH mRNA levels (D). Triplicate experiments were performed.

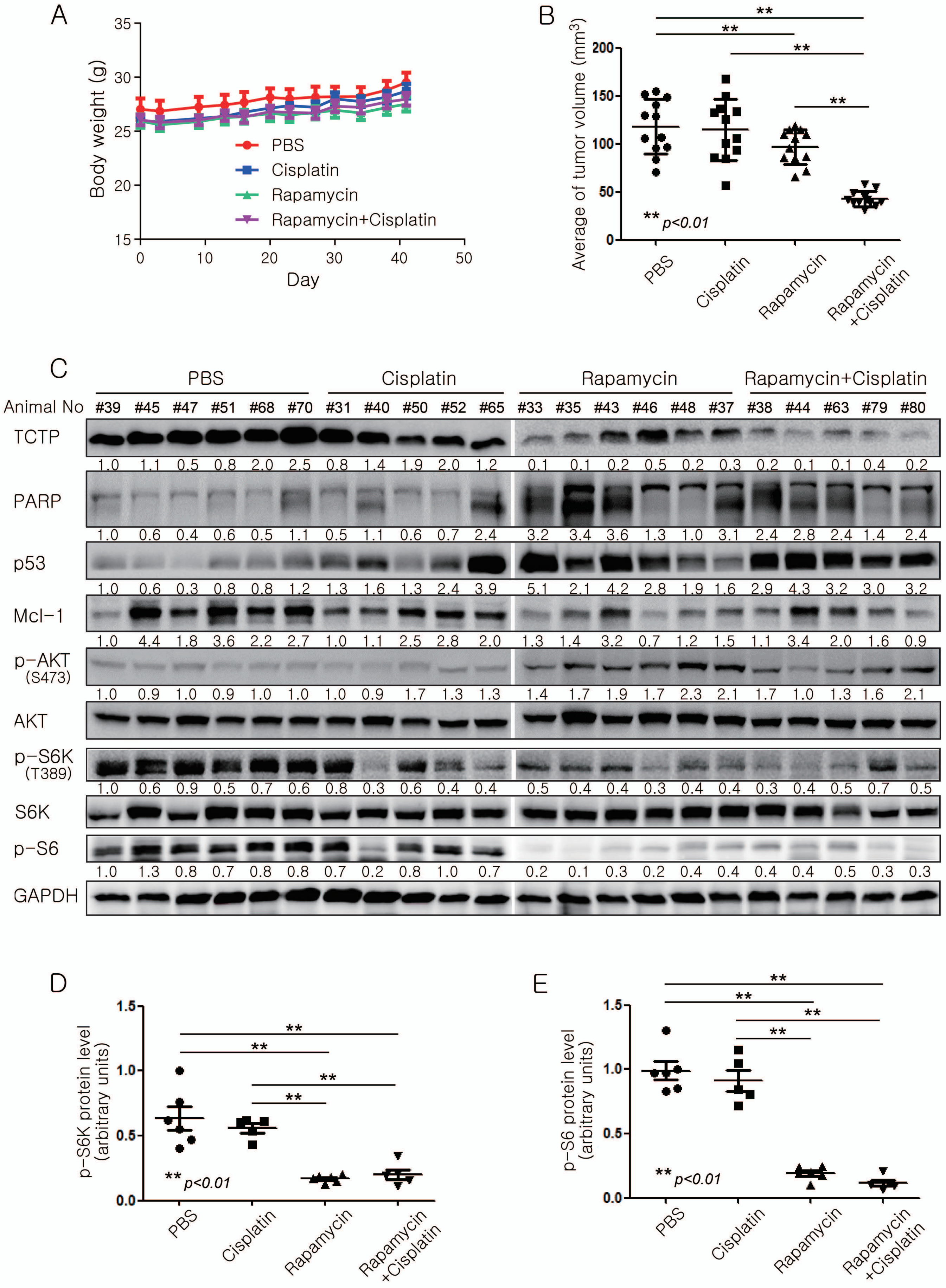
A
Body weight (g)
35
30
25
20
15
0
10
20
30
40
50
Day
PBS
Cisplatin
Rapamycin
Rapamycin+Cisplatin
B
Average of tumor volume (mm3)
200
150
100
50
0
** p<0.01
**
PBS
Cisplatin
Rapamycin
Rapamycin +Cisplatin
C
PBS
Cisplatin
Rapamycin
Rapamycin+Cisplatin
Animal No #39 #45 #47 #51 #68 #70 #31 #40 #50 #52 #65 #33 #35 #43 #46 #48 #37 #38 #44 #63 #79 #80
TCTP
1.0 1.1 0.5 0.8 2.0 2.5 0.8 1.4 1.9 2.0 1.2 0.1 0.1 0.2 0.5 0.2 0.3 0.2 0.1 0.1 0.4 0.2
PARP
1.0 0.6 0.4 0.6 0.5 1.1 0.5 1.1 0.6 0.7 2.4 3.2 3.4 3.6 1.3 1.0 3.1 2.4 2.8 2.4 1.4 2.4
p53
1.0 0.6 0.3 0.8 0.8 1.2 1.3 1.6 1.3 2.4 3.9 5.1 2.1 4.2 2.8 1.9 1.6 2.9 4.3 3.2 3.0 3.2
Mcl-1
1.0 4.4 1.8 3.6 2.2 2.7 1.0 1.1 2.5 2.8 2.0 1.3 1.4 3.2 0.7 1.2 1.5 1.1 3.4 2.0 1.6 0.9
p-AKT (S473)
1.0 0.9 1.0 0.9 1.0 1.0 1.0 0.9 1.7 1.3 1.3 1.4 1.7 1.9 1.7 2.3 2.1 1.7 1.0 1.3 1.6 2.1
AKT
p-S6K (T389)
1.0 0.6 0.9 0.5 0.7 0.6 0.8 0.3 0.6 0.4 0.4 0.5 0.4 0.4 0.3 0.4 0.4 0.3 0.4 0.5 0.7 0.5
S6K
p-S6
1.0 1.3 0.8 0.7 0.8 0.8 0.7 0.2 0.8 1.0 0.7 0.2 0.1 0.3 0.2 0.4 0.4 0.4 0.4 0.5 0.3 0.3
GAPDH
D
p-S6K protein level (arbitrary units)
1.5
1.0
0.5
0.0
** p<0.01
**
PBS
Cisplatin
Rapamycin
Rapamycin +Cisplatin
E
p-S6 protein level (arbitrary units)
1.5
1.0
0.5
0.0
** p<0.01
**
PBS
Cisplatin
Rapamycin
Rapamycin +Cisplatin

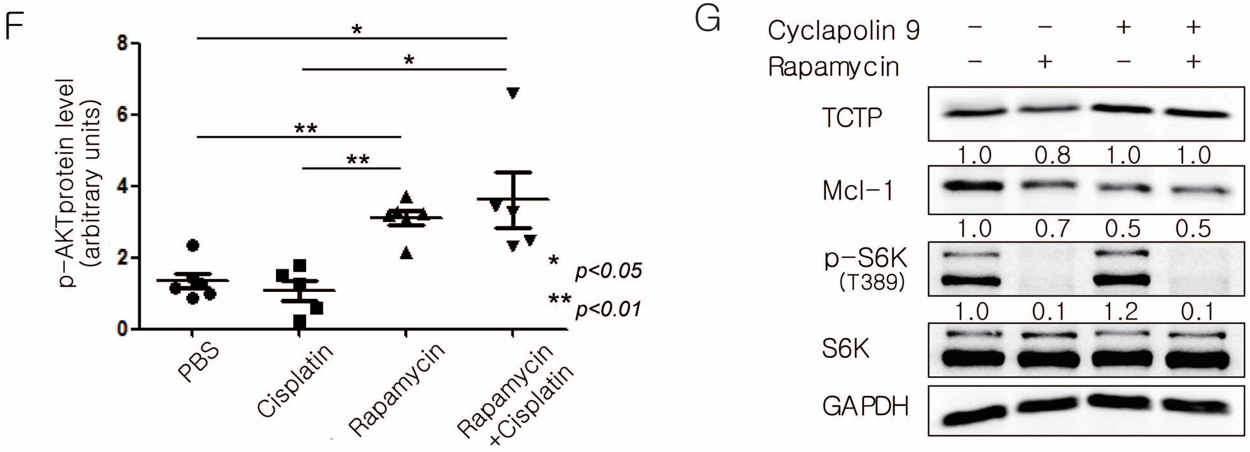

Supplementary Figure S4. Rapamycin augments the efficacy of cisplatin and induces S6K inhibition and Akt activation in an A549 lung cancer xenograft model. (A) The body weight of the mice was measured as an indicator of general health conditions. (B) The within-group of tumor volumes (mm3) at each measurement point (shown in Fig. 7B) are cumulatively presented as means ± SD. (C) Tumor tissue homogenates were analyzed by immunoblotting and triplicate experiments were performed. (D-F) The expression levels of p-S6K1, p-S6, and p-Akt obtained in Supplementary Fig. S4C were quantified, normalized to the total forms or GAPDH, and are presented as the mean ± SD. (G) Down-regulation of Mcl-1 expression by rapamycin was abolished by pretreatment with PLK1 inhibitor. A549 cells were preincubated for 2 hours with 5 μM cyclapolin 9 and then treated for 24 hours with 100 pM rapamycin.

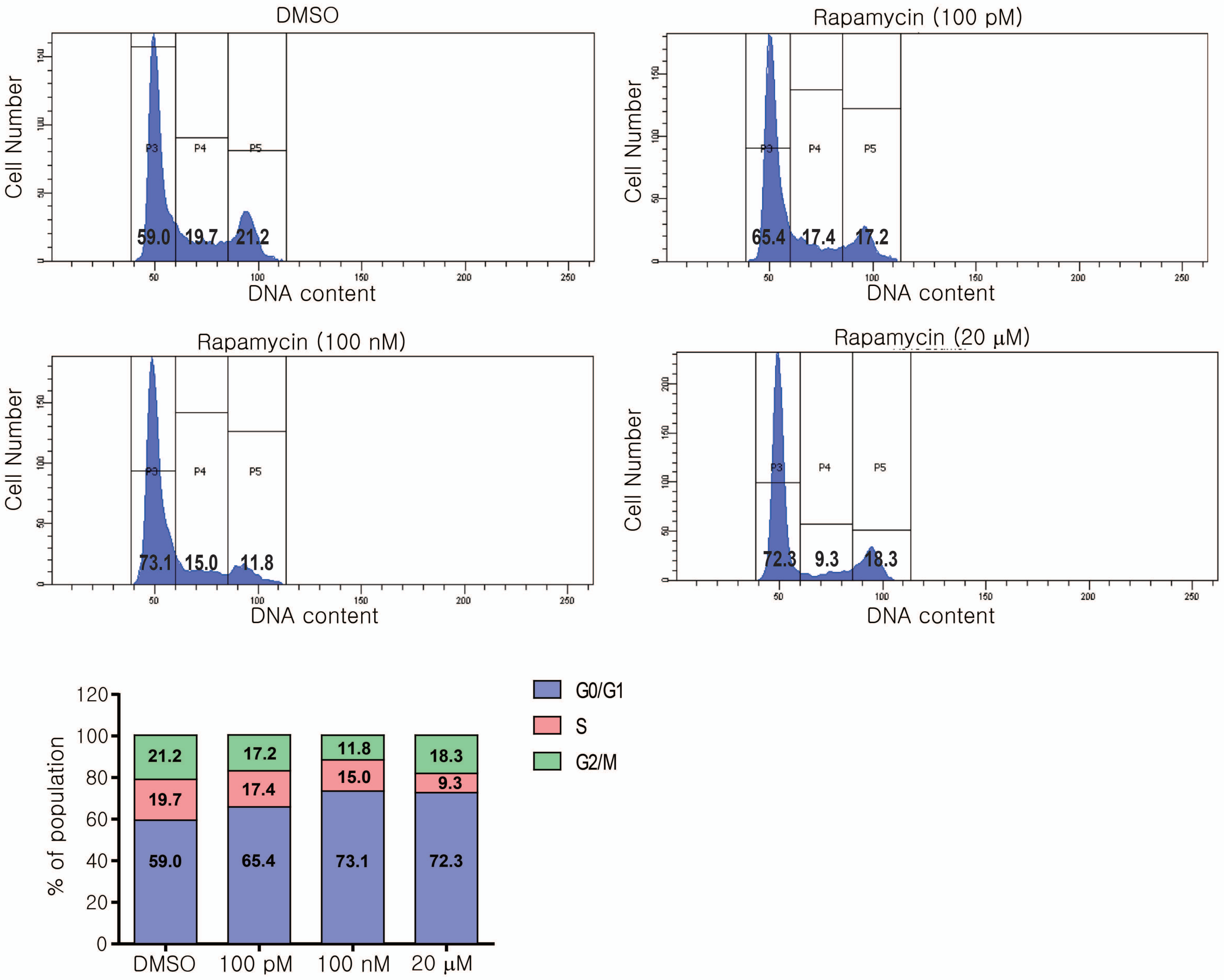

Supplementary Figure S5. Treatment with 100 pM rapamycin, a concentration adequate to reduce the TCTP protein level, has little effect on cell cycle progression. Non-synchronized A549 cells were treated for 24 hours with various concentrations of rapamycin. For analysis of DNA content, cells were stained for 30 minutes at 37°C using Dulbecco's phosphate buffered saline containing 100 μg/ml of propidium iodide and 20 μg/ml of RNase, and the percentages of cells in the G1, S, and G2/M phases of the cell cycle were determined by flow cytometry. DNA content histograms and cell cycle distributions are shown in the upper and lower panels, respectively. Duplicate experiments were performed.

A

Primers for plasmid construction		
TCTP(Ser46Ala)	Forward	5′-ATTGATGAC***G****CG*CTCATTGG-3′
	Reverse	5′-CCACCAATGAG*CG****C***GTCA-3′
TCTP (Ser64Ala)	Forward	5′-GCGAAGGTACCGAA***GCA***ACAG-3′
	Reverse	5′- ACCAGTGATTACTGT***TGC***TTCGGT-3′
TCTP (Thr65Val)	Forward	5′-GCGAAGGTACCGAAAGC***GT****A*GT-3′
	Reverse	5′-GACACCAGTGATTAC*T****AC***GCTTTCG-3′

B

The sequences of siRNAs	
Raptor (NM_020761.2)	5′-CCUCACUUUAUUUCCAUGU(dTdT)-3′
Rictor (NM_001285439.2)	5′- GUCCUUCACGAGAGACAGU(dTdT)-3′
PLK1 (NM_005030.3)	5′-CCGGGAAAAAGAAGAACCAG(dTdT)-3′
TCTP (NM_003295.2)	5′- GCAUGGUUGCUCUAUUGGA(dTdT)-3′
GFP	5′-GCAUCAAGGUGAACUUCAA(dTdT)-3′
AccuTarget<br>Negative control siRNA	5′-CCUACGCCACCAAUUUCGU(dTdT)-3′

C

Primers for qRT-PCR		
TCTP (NM_003295.2)	Forward	5′-ATCCAGATGGCATGGTTGCT-3′
	Reverse	5′-CGCAGGGATTTCTTTCTTTTGC-3′
GAPDH (NM_001256799.3)	Forward	5′-CAATGACCCCTTCATTGACC-3′
	Reverse	5′-GATCTCGCTCCTGGAAGATG-3′

D

Name	Supplier	Cat no.
TCTP	Abcam	ab37506
phospho-TCTP-Ser46	Cell Signaling Technology	5251
Raptor	Cell Signaling Technology	2280
Rictor	Cell Signaling Technology	2114
S6K	Cell Signaling Technology	2708
phospho-S6K-T389	Cell Signaling Technology	9234
Cdc25C	Cell Signaling Technology	4688
phospho-Cdc25C-Ser198	Cell Signaling Technology	9529
Akt	Cell Signaling Technology	9272
phospho-Akt-T308	Cell Signaling Technology	9275
phospho-Akt-S473	Cell Signaling Technology	9271
PLK1	Cell Signaling Technology	4513
phospho-PLK1-Thr210	Cell Signaling Technology	9062
phospho-S6	eBioscience	14-9007
Ubiquitin	Santa Cruz Biotech	sc-8017
HA	Santa Cruz Biotech	sc-7392
GAPDH	Santa Cruz Biotech	sc-32233
Mcl-1	Santa Cruz Biotech	sc-12756
PARP	Santa Cruz Biotech	sc-8007
p53	Santa Cruz Biotech	sc-126
β-actin	AB Frontier, Korea	LF-PA0209
α-tubulin	AB Frontier, Korea	LF-PA0146

Supplementary Table S1. (A) Primers for plasmid construction (B) The sequences of siRNAs (C) Primers for qRT-PCR (D) Antibody information

Supplementary Information

(Original blot/gel images)

TCTP protein degradation by targeting mTORC1 and signaling through S6K, Akt and Plk1 sensitizes lung cancer cells to DNA-damaging drugs.

Mini Jeong, Mi Hyeon Jeong, Jung Eun Kim, Serin Cho, Kyoung Jin Lee, Serkin Park, Jeongwon Sohn, Yun Gyu Park

Figure 1A

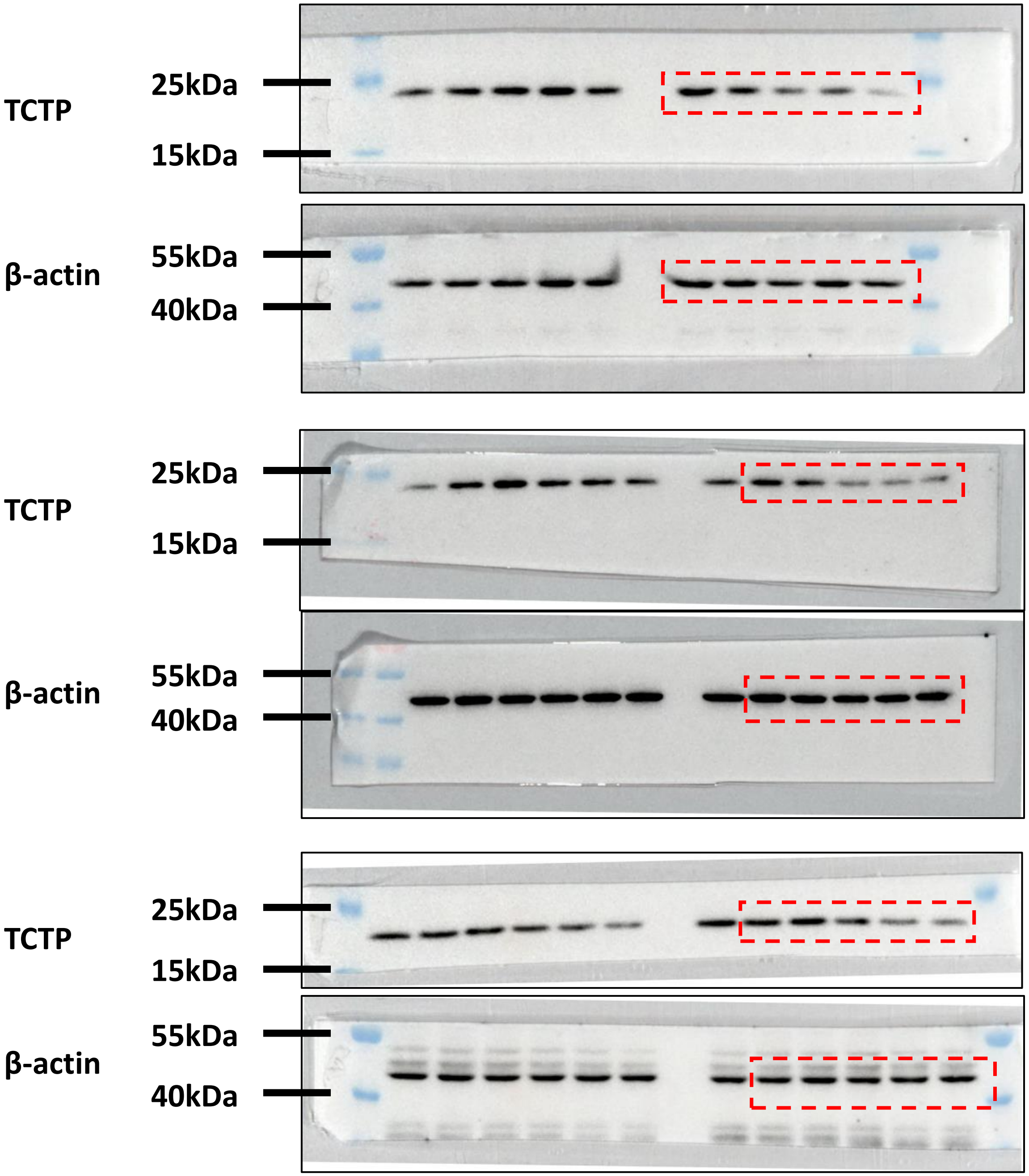

TCTP
25kDa
15kDa
β-actin
55kDa
40kDa
TCTP
25kDa
15kDa
β-actin
55kDa
40kDa
TCTP
25kDa
15kDa
β-actin
55kDa
40kDa

Figure 1C

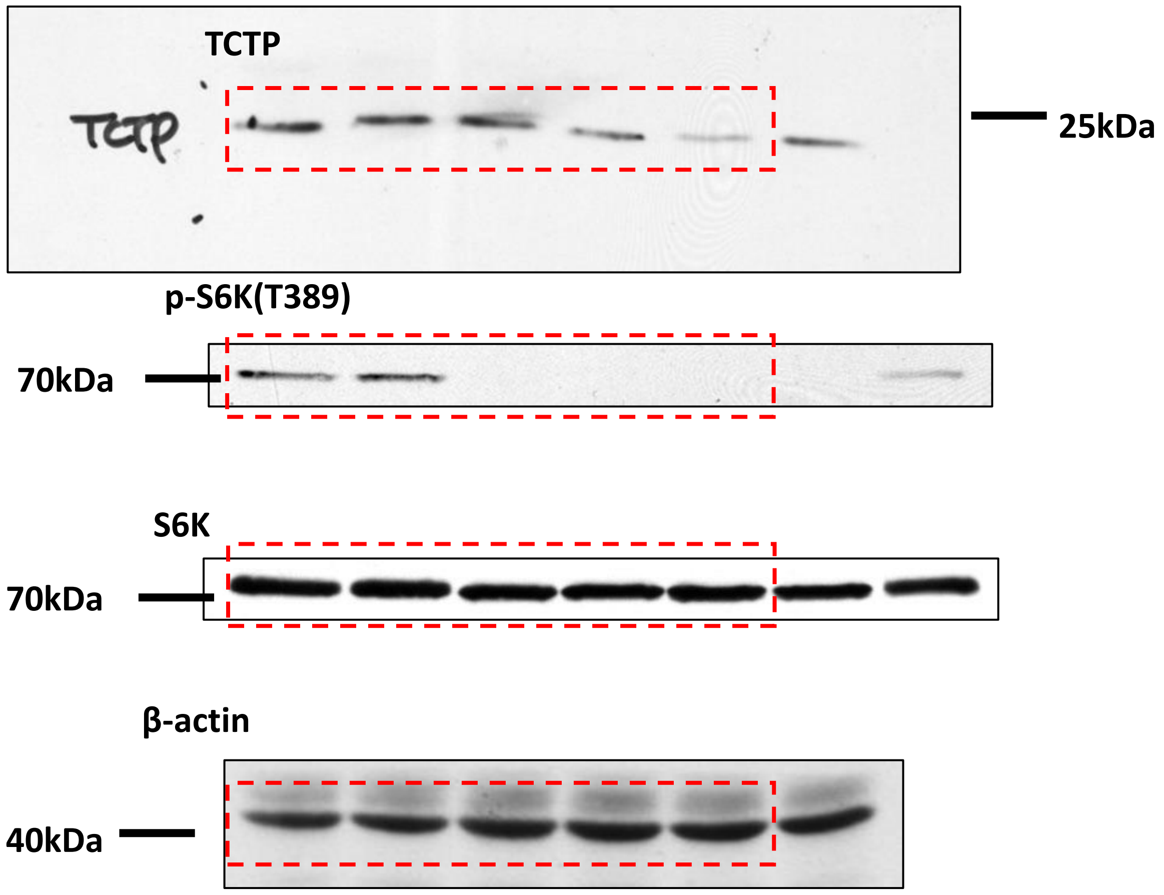

Figure 1D

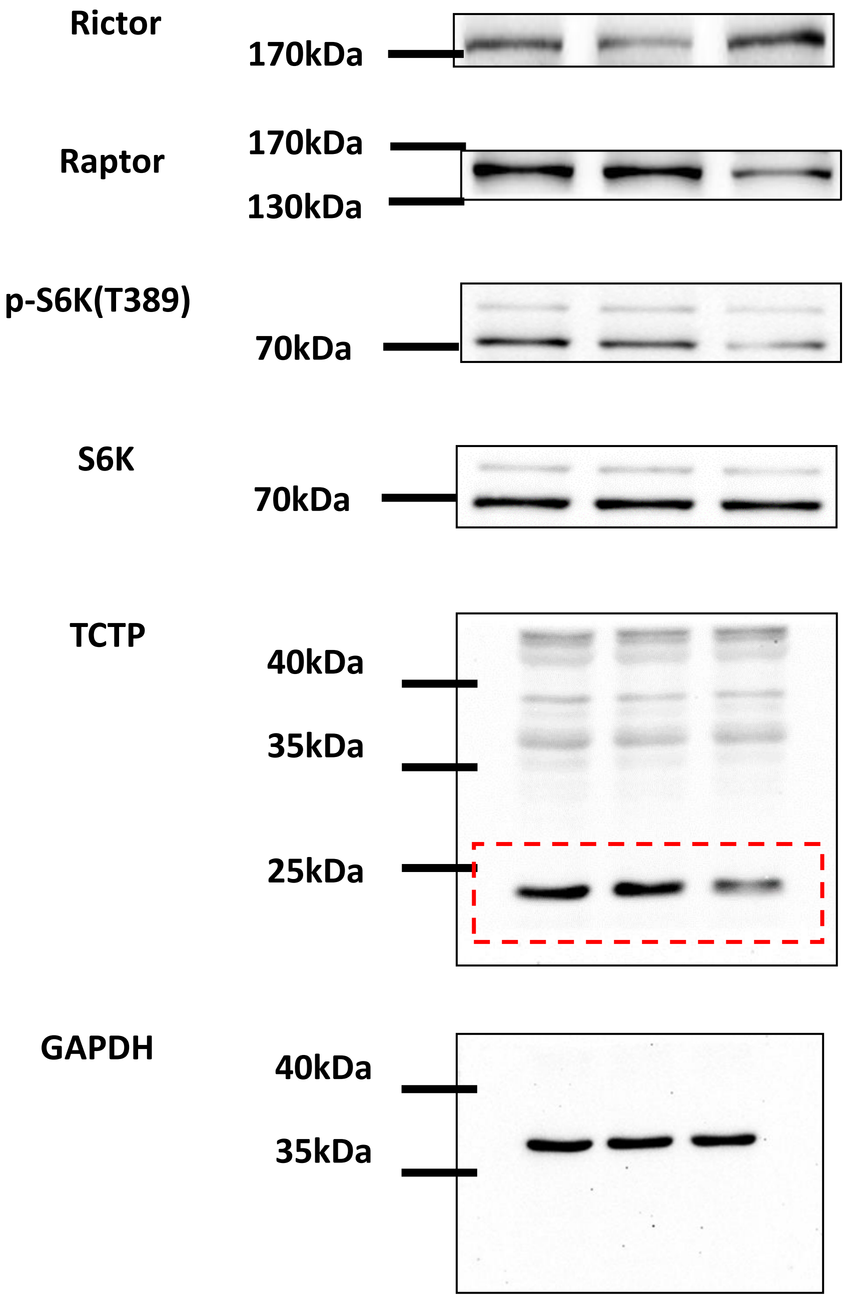
Rictor
170kDa
Raptor
170kDa
130kDa
p-S6K(T389)
70kDa
S6K
70kDa
TCTP
40kDa
35kDa
25kDa
GAPDH
40kDa
35kDa

Figure 1E

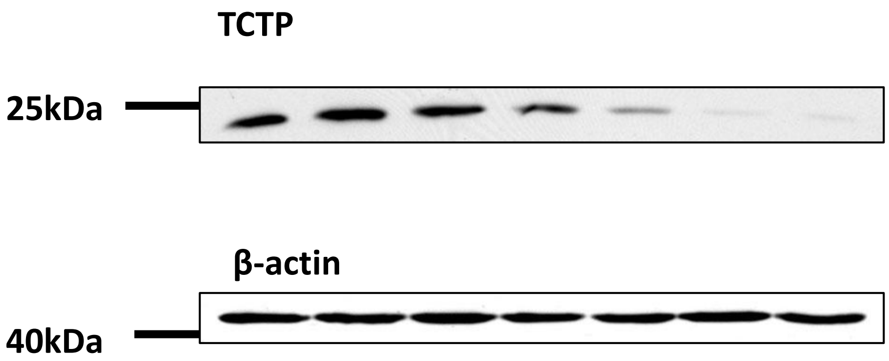

Figure 2A

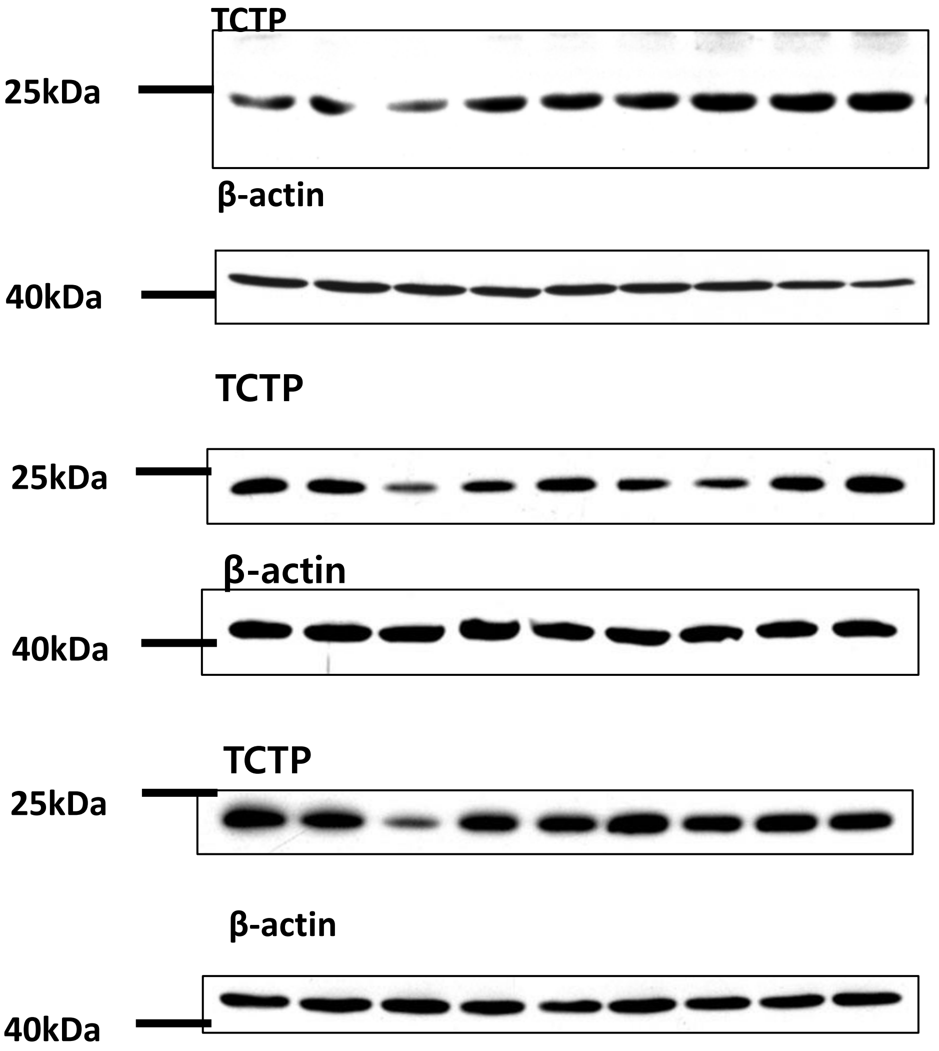
TCTP
25kDa
β-actin
40kDa
TCTP
25kDa
β-actin
40kDa
TCTP
25kDa
β-actin
40kDa

Figure 2B

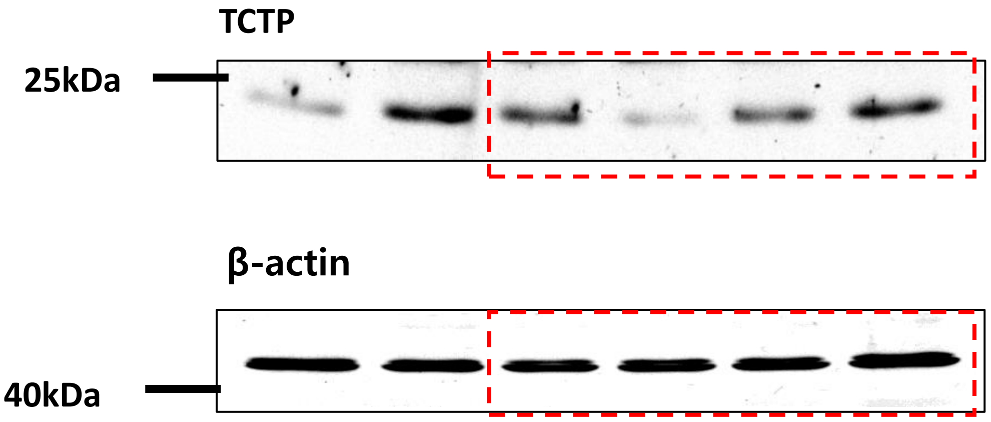

Figure 2C

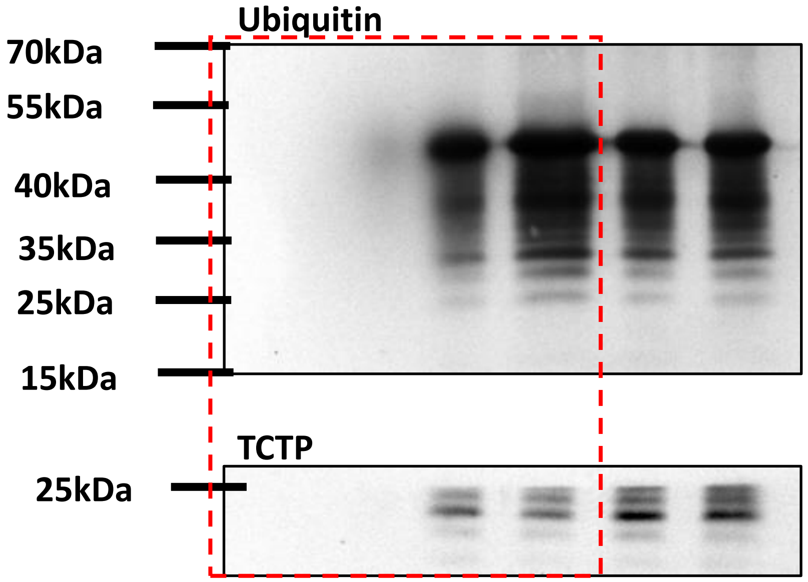

Figure 2D

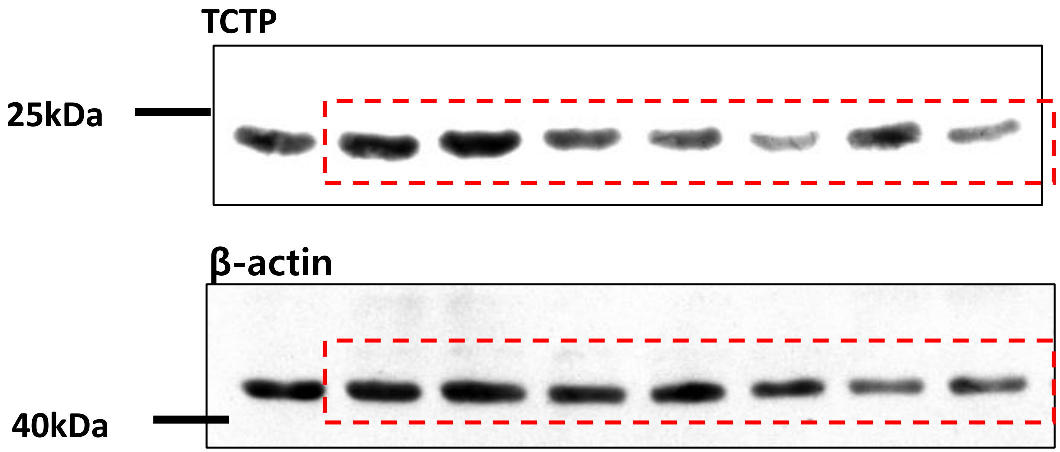

Figure 3A

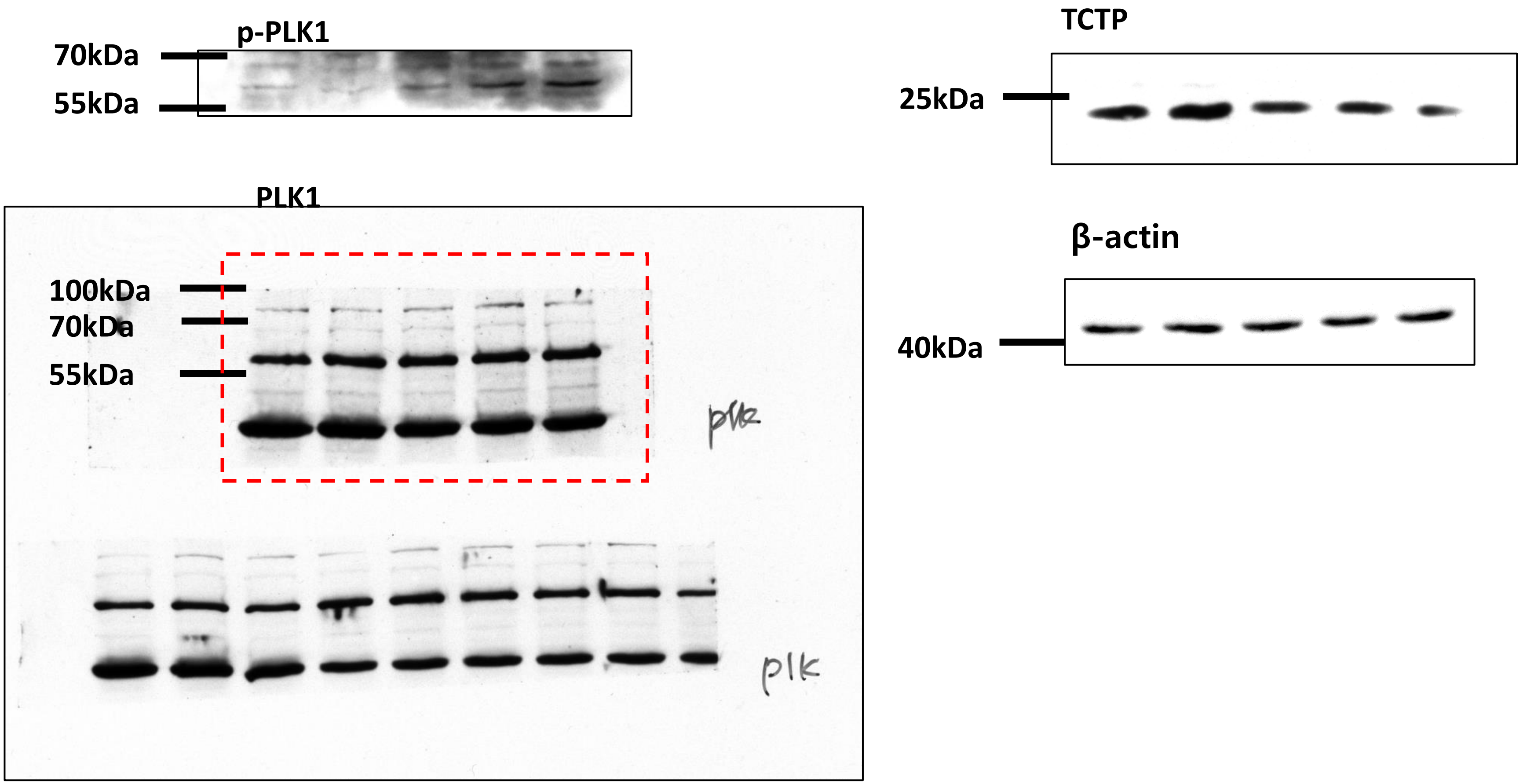
p-PLK1
70kDa
55kDa
TCTP
25kDa
PLK1
100kDa
70kDa
55kDa
plk
plk
β-actin
40kDa

Figure 3A

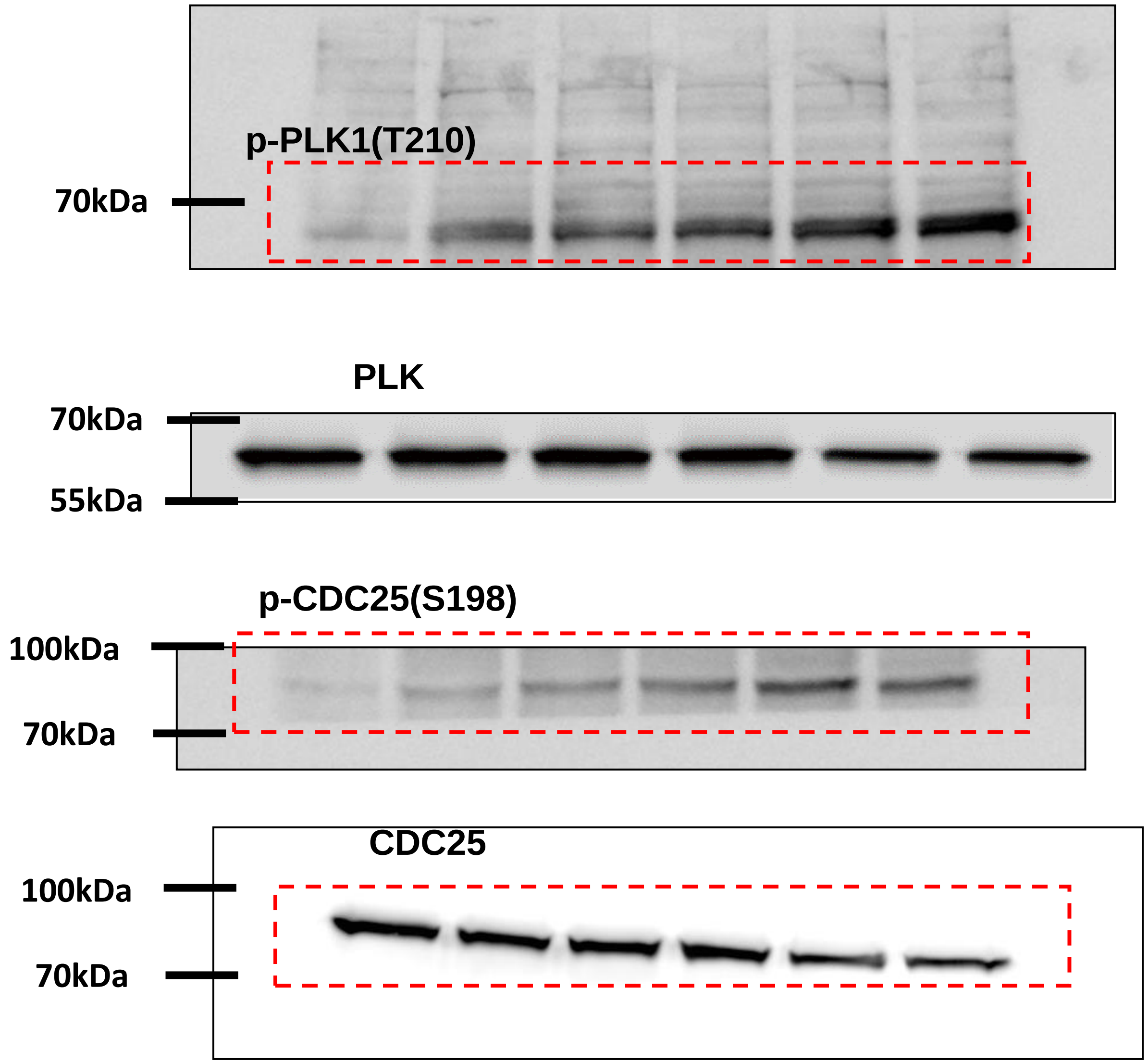
p-PLK1(T210)
70kDa
PLK
70kDa
55kDa
p-CDC25(S198)
100kDa
70kDa
CDC25
100kDa
70kDa

Figure 3B

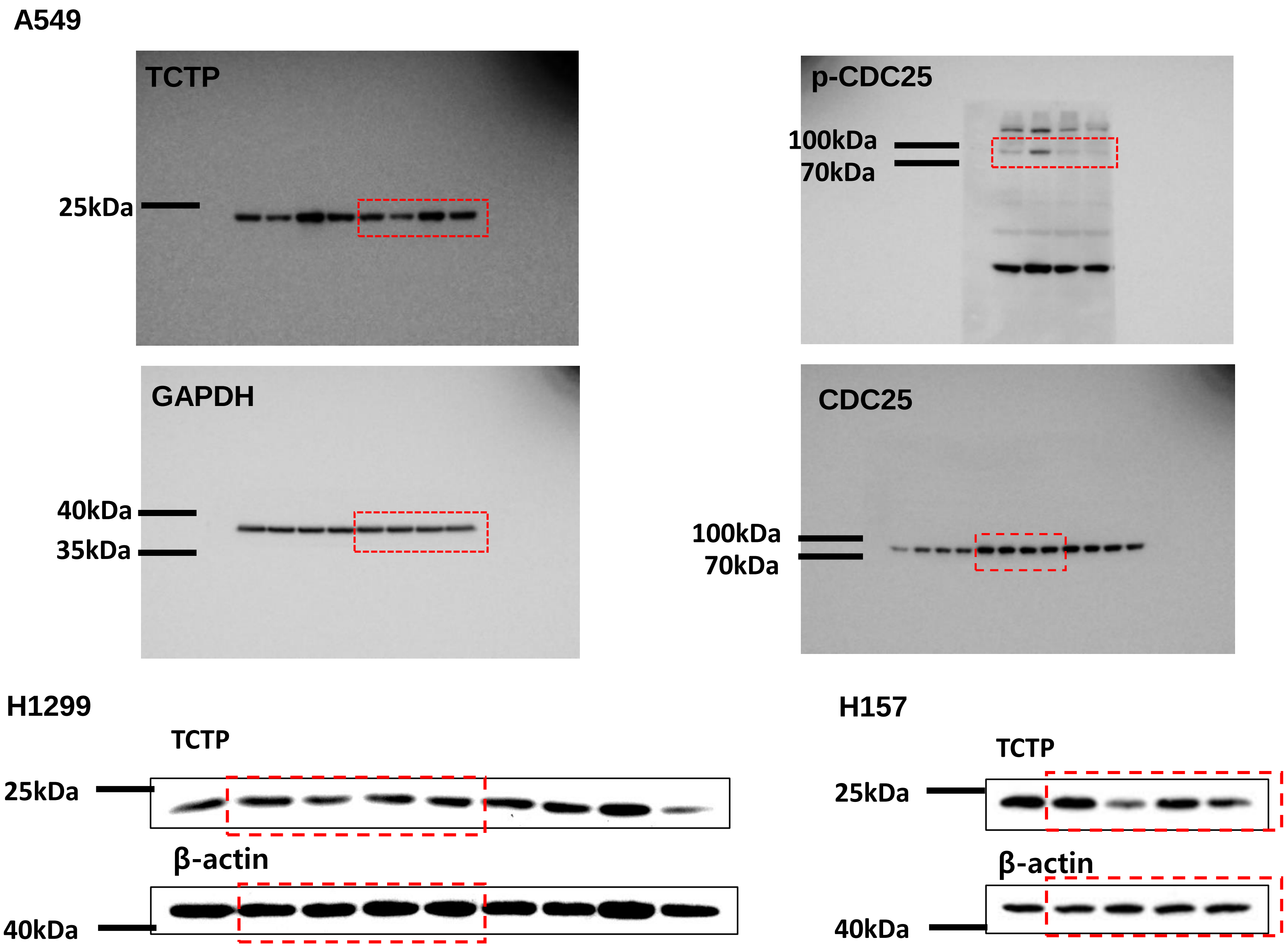
A549
TCTP
25kDa
p-CDC25
100kDa
70kDa
GAPDH
40kDa
35kDa
CDC25
100kDa
70kDa
H1299
TCTP
25kDa
β-actin
40kDa
H157
TCTP
25kDa
β-actin
40kDa

Figure 3C

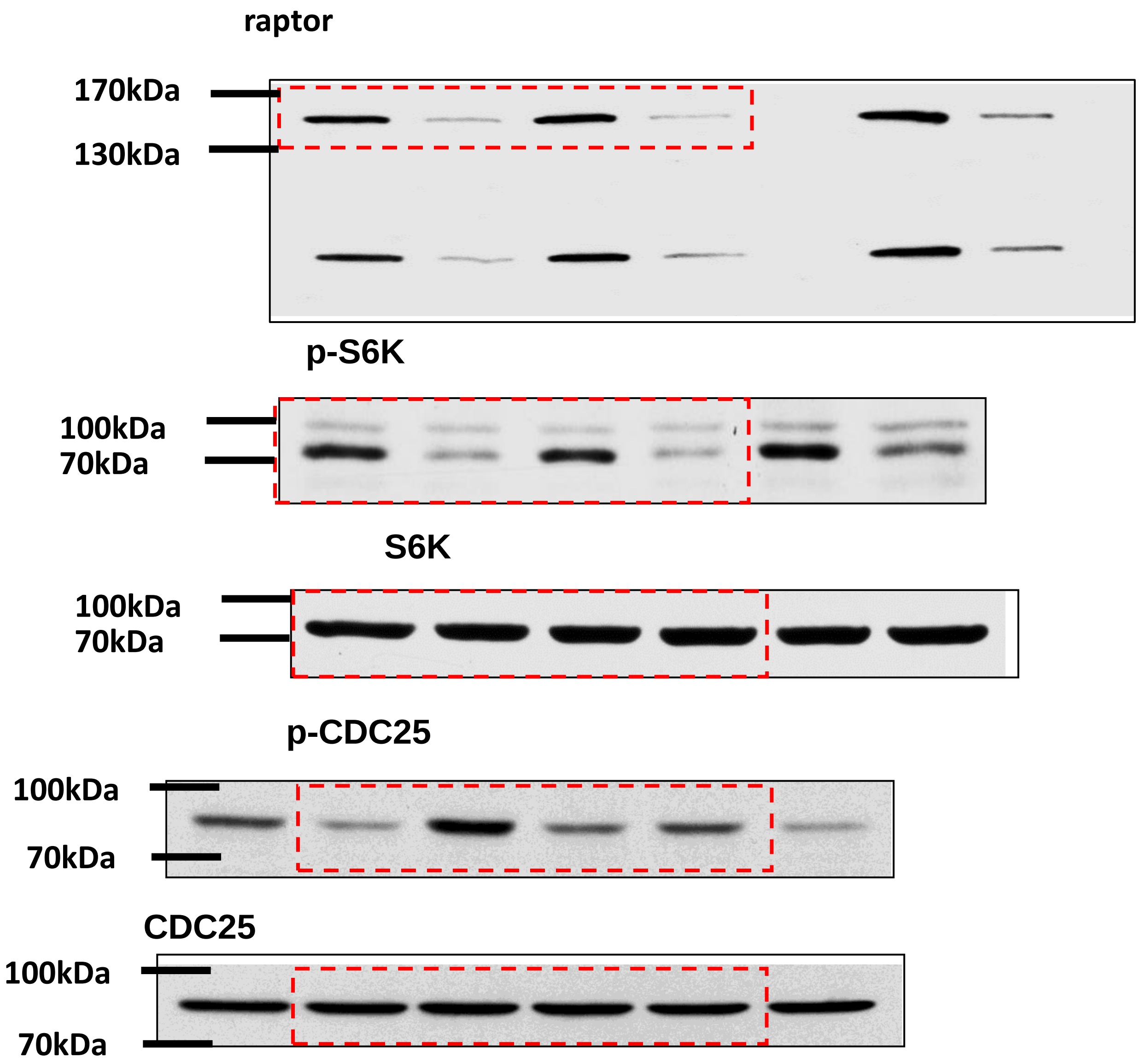
raptor
170kDa
130kDa
p-S6K
100kDa
70kDa
S6K
100kDa
70kDa
p-CDC25
100kDa
70kDa
CDC25
100kDa
70kDa

Figure 3C

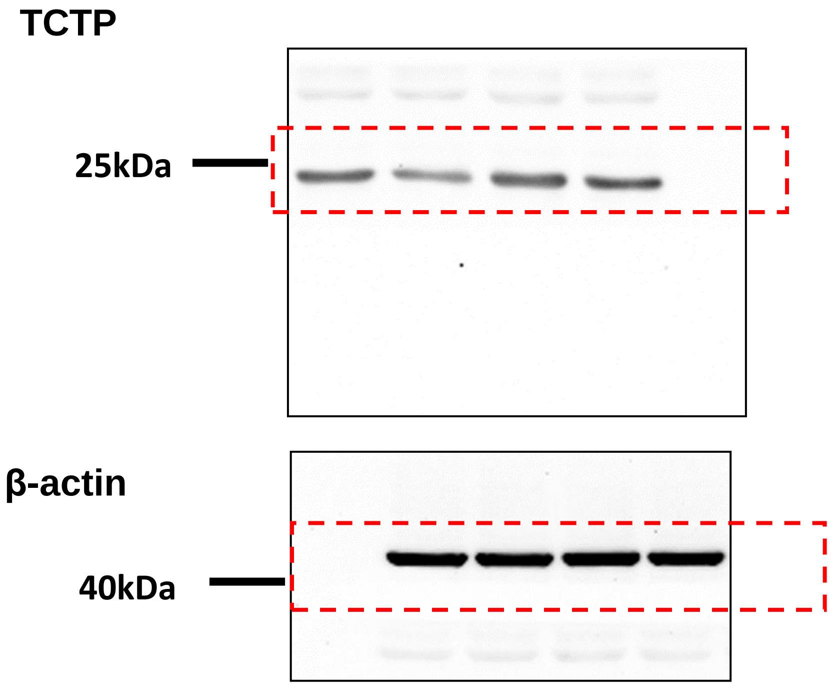

Figure 3D

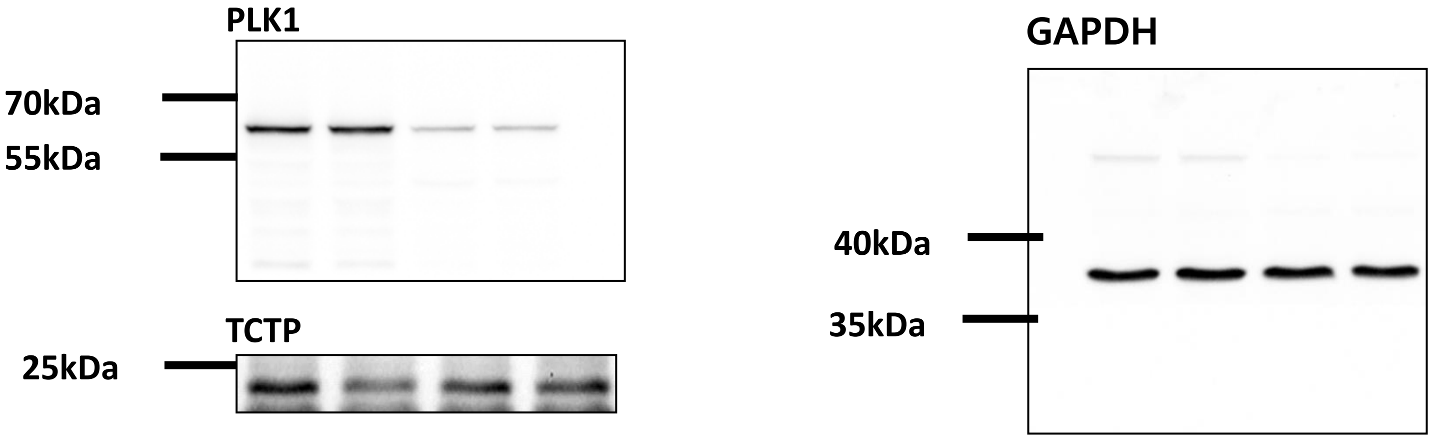

Figure 3E

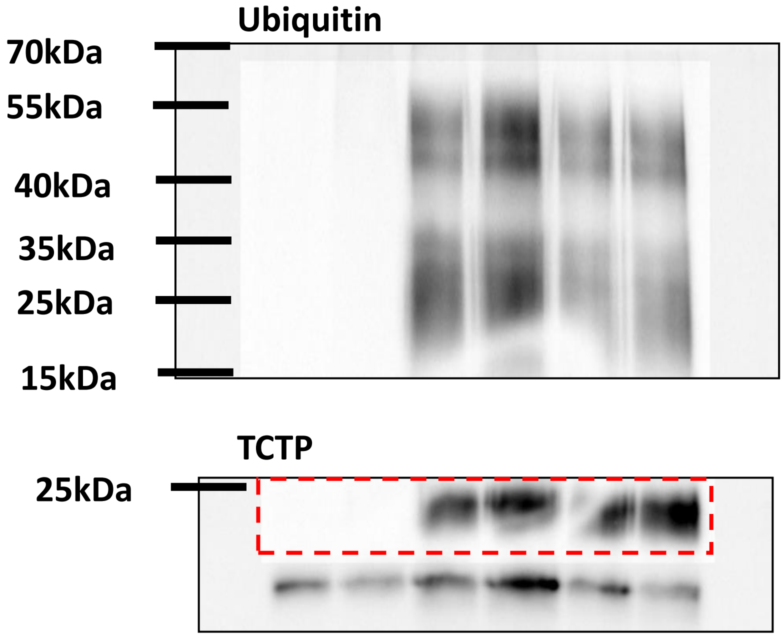

Figure 4A

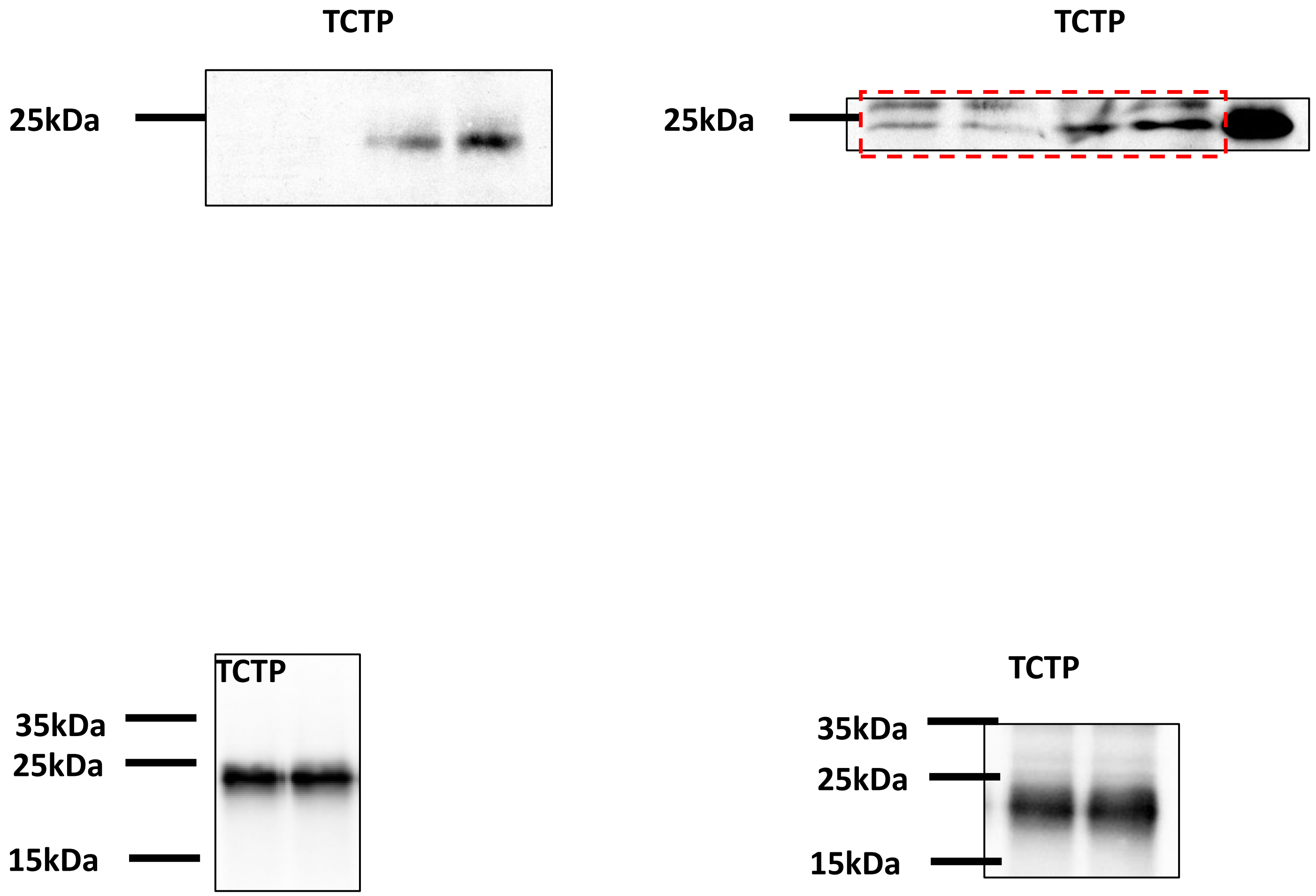
TCTP
25kDa
TCTP
25kDa
TCTP
35kDa
25kDa
15kDa
TCTP
35kDa
25kDa
15kDa

Figure 4B

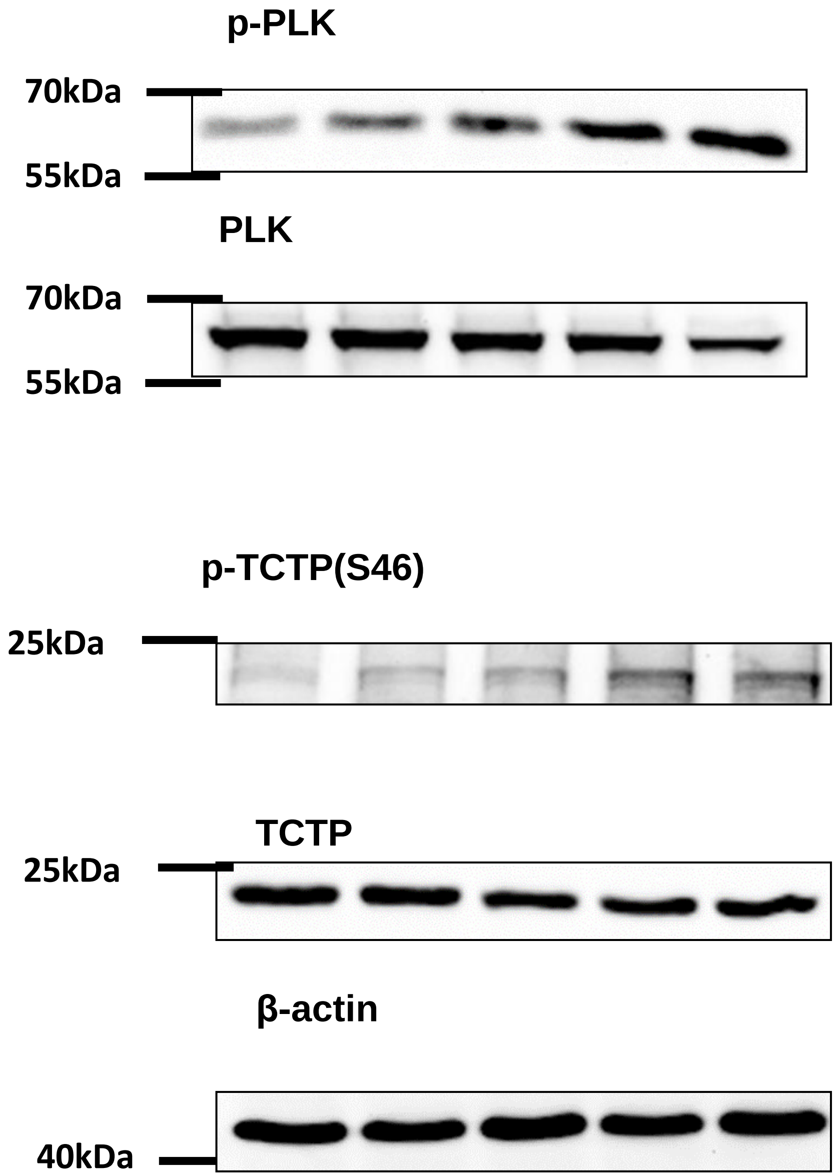

p-PLK
70kDa
55kDa
PLK
70kDa
55kDa
p-TCTP(S46)
25kDa
TCTP
25kDa
β-actin
40kDa

Figure 4C

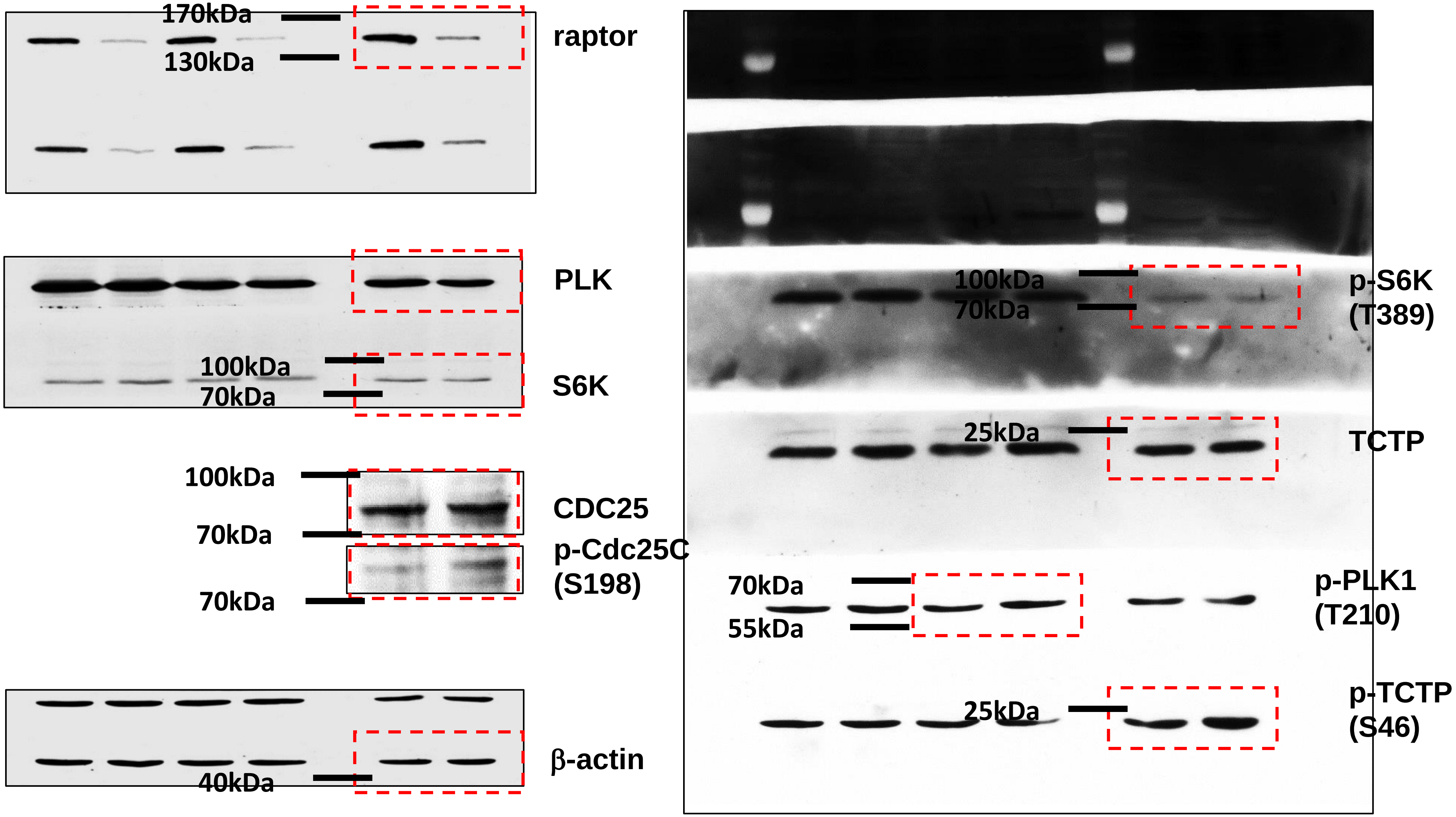

170kDa
130kDa
raptor
PLK
100kDa
70kDa
S6K
100kDa
70kDa
CDC25
p-Cdc25C
(S198)
70kDa
40kDa
β-actin
100kDa
70kDa
p-S6K
(T389)
25kDa
TCTP
70kDa
55kDa
p-PLK1
(T210)
25kDa
p-TCTP
(S46)

Figure 4D

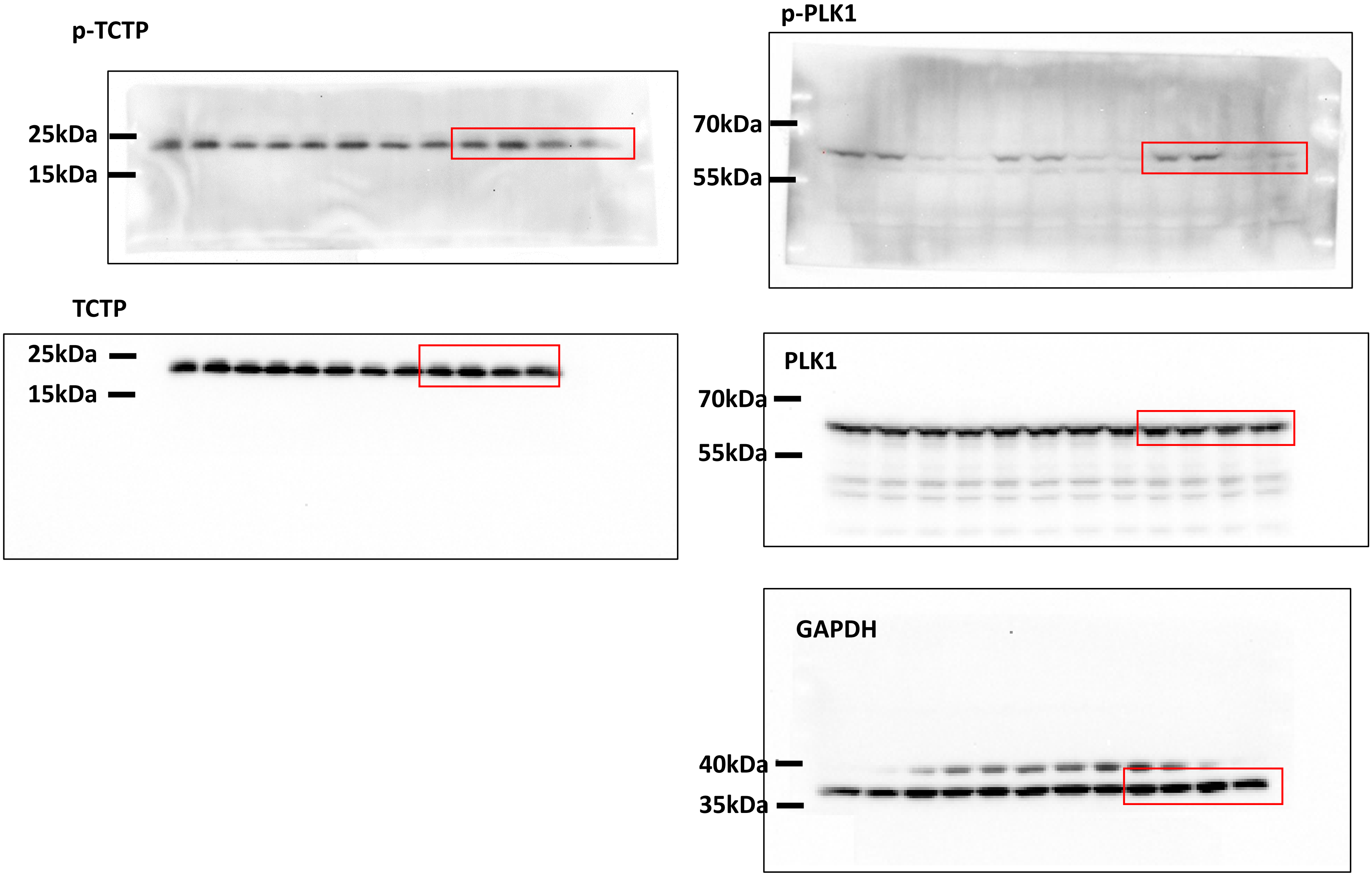
p-TCTP
25kDa
15kDa
TCTP
25kDa
15kDa
p-PLK1
70kDa
55kDa
PLK1
70kDa
55kDa
GAPDH
40kDa
35kDa

Figure 4E

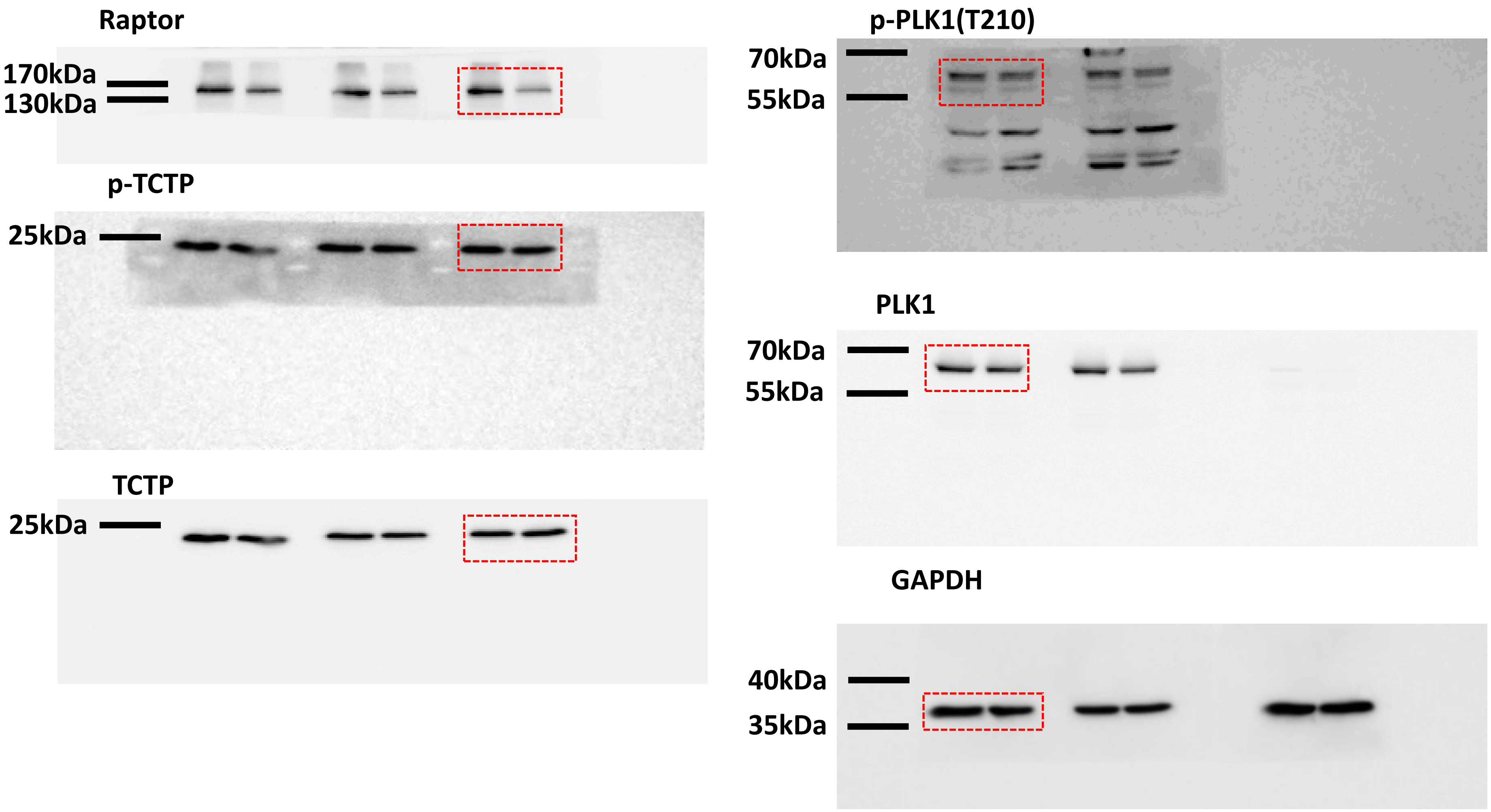
Raptor
170kDa
130kDa
p-TCTP
25kDa
TCTP
25kDa
p-PLK1(T210)
70kDa
55kDa
PLK1
70kDa
55kDa
GAPDH
40kDa
35kDa

Figure 4F

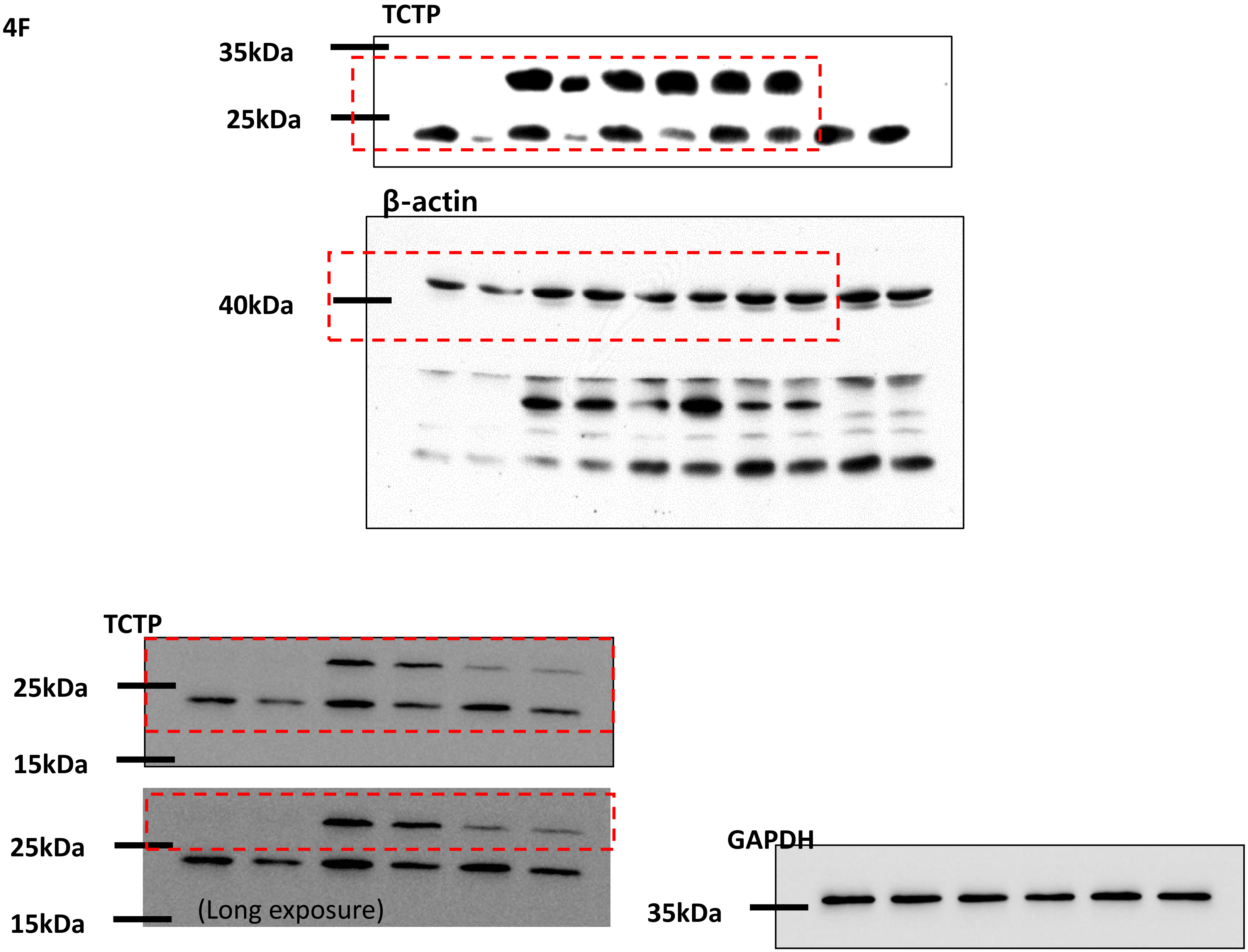

TCTP
35kDa
25kDa
β-actin
40kDa
TCTP
25kDa
15kDa
25kDa
15kDa
(Long exposure)
GAPDH
35kDa

Figure 5A

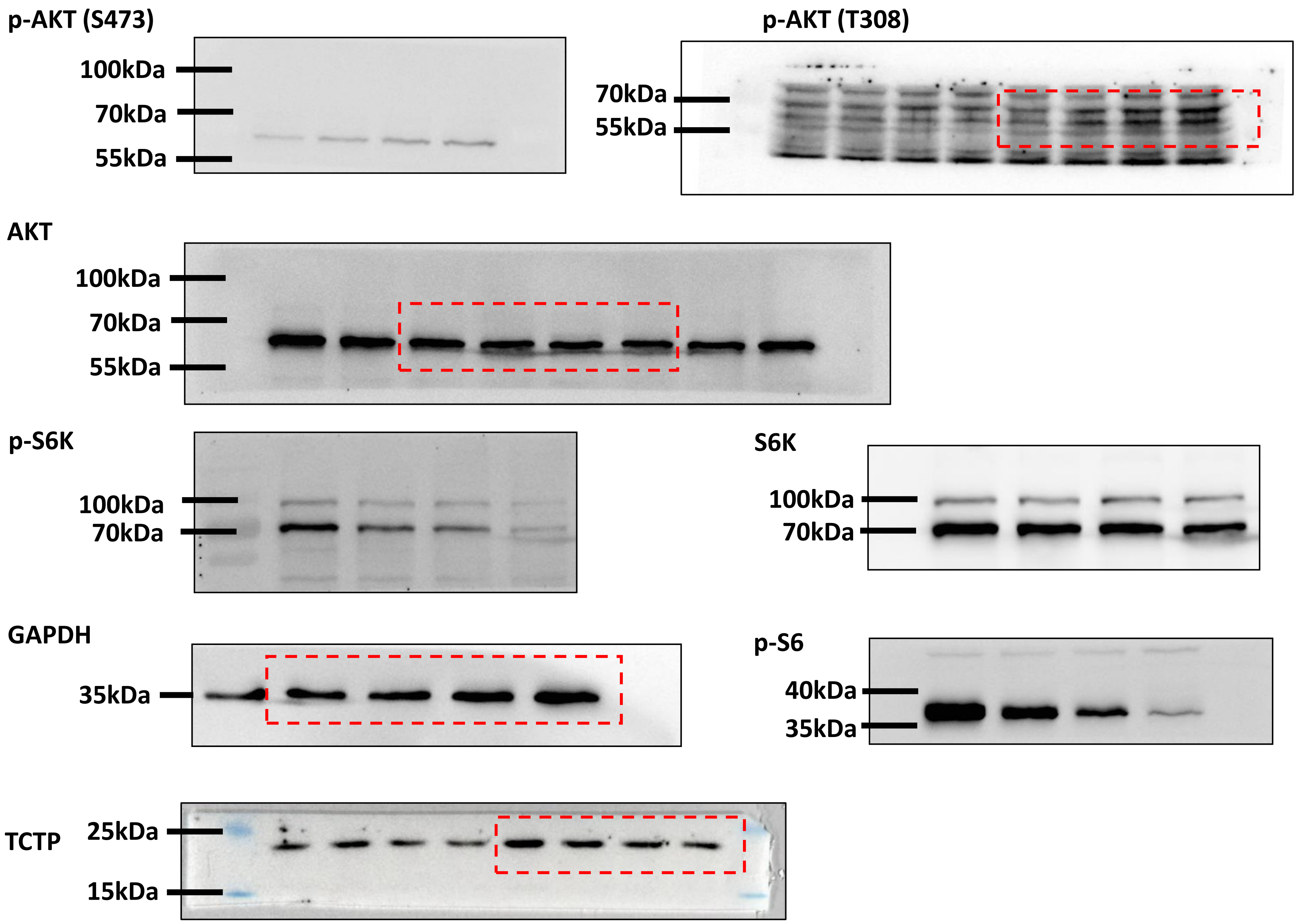
p-AKT (S473)
100kDa
70kDa
55kDa
p-AKT (T308)
70kDa
55kDa
AKT
100kDa
70kDa
55kDa
p-S6K
100kDa
70kDa
S6K
100kDa
70kDa
GAPDH
35kDa
p-S6
40kDa
35kDa
TCTP
25kDa
15kDa

Figure 5B

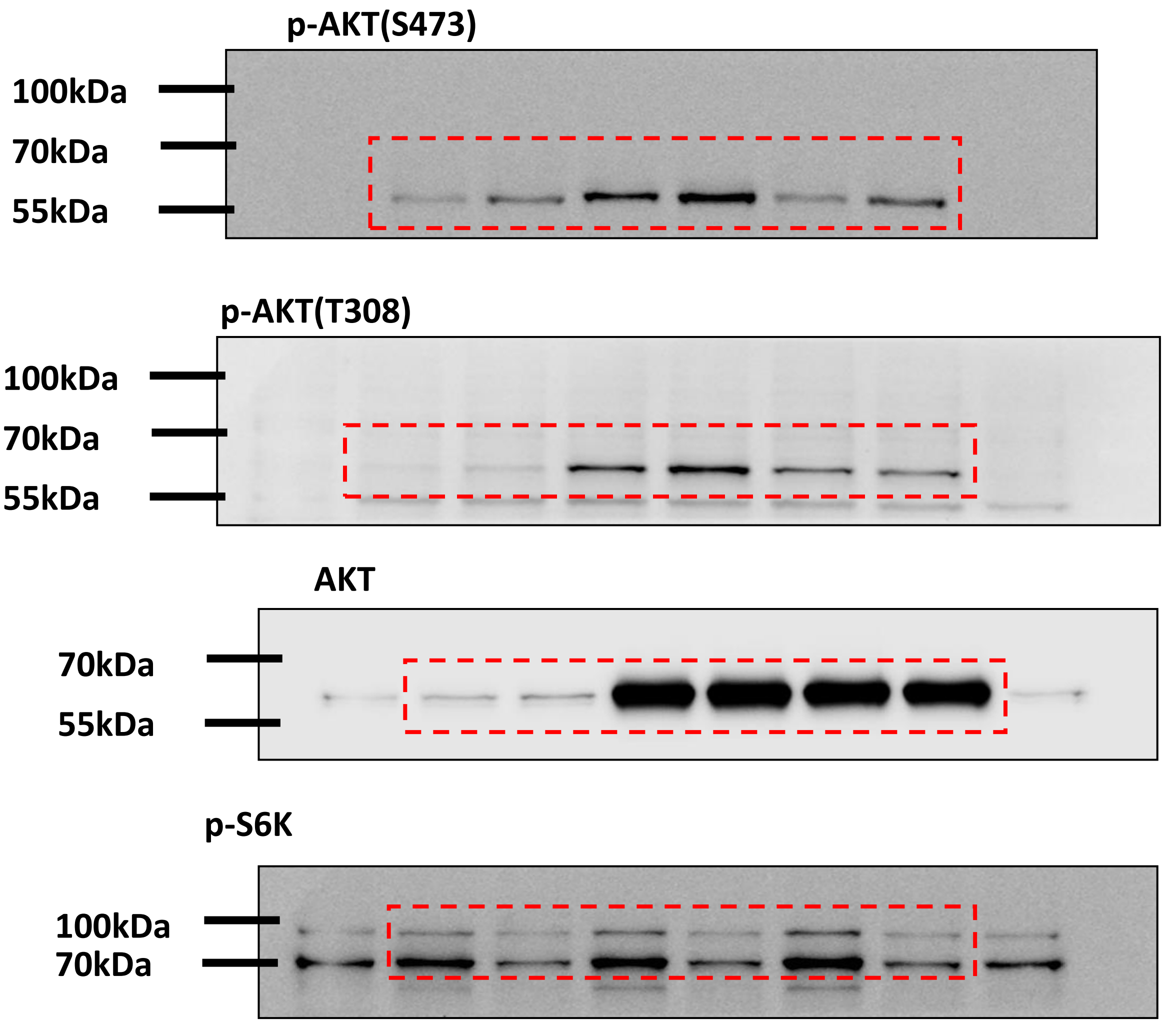
p-AKT(S473)
100kDa
70kDa
55kDa
p-AKT(T308)
100kDa
70kDa
55kDa
AKT
70kDa
55kDa
p-S6K
100kDa
70kDa

Figure 5B

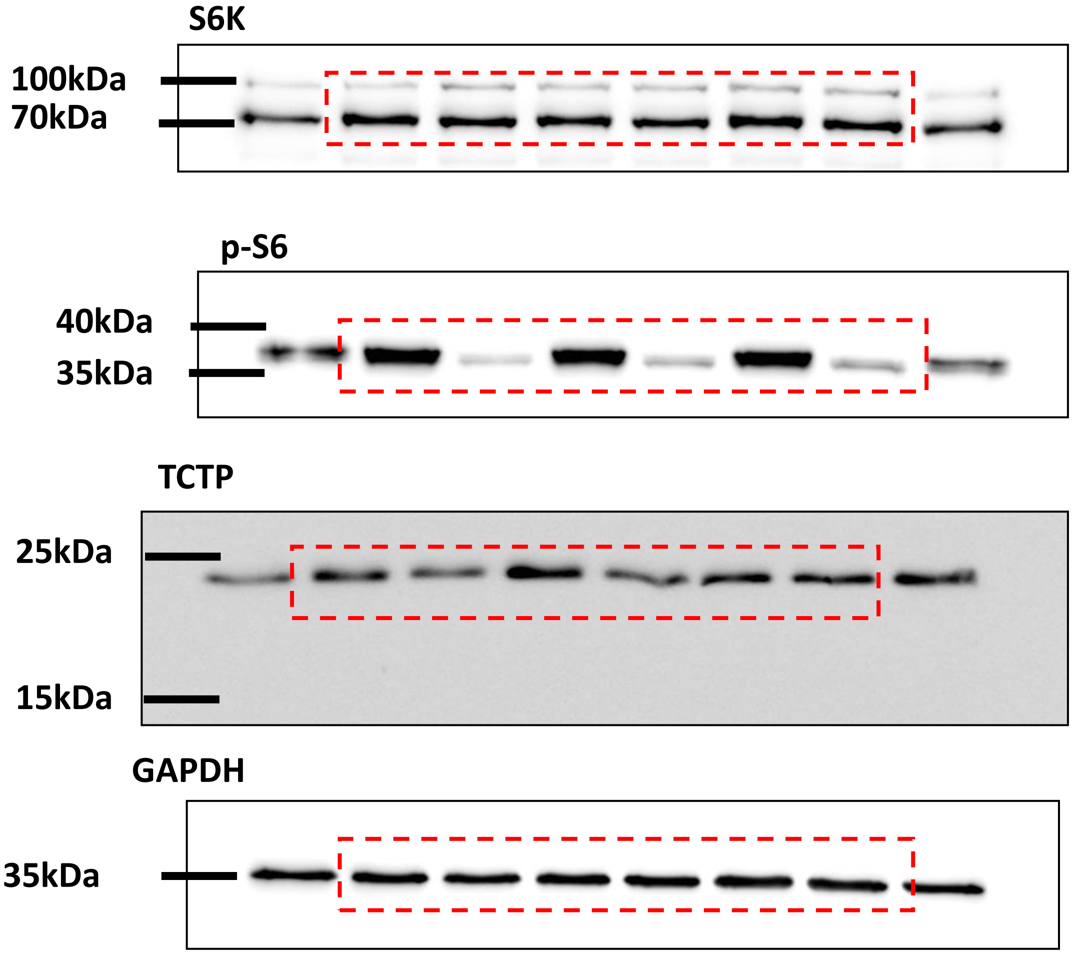
S6K
100kDa
70kDa
p-S6
40kDa
35kDa
TCTP
25kDa
15kDa
GAPDH
35kDa

Figure 5C

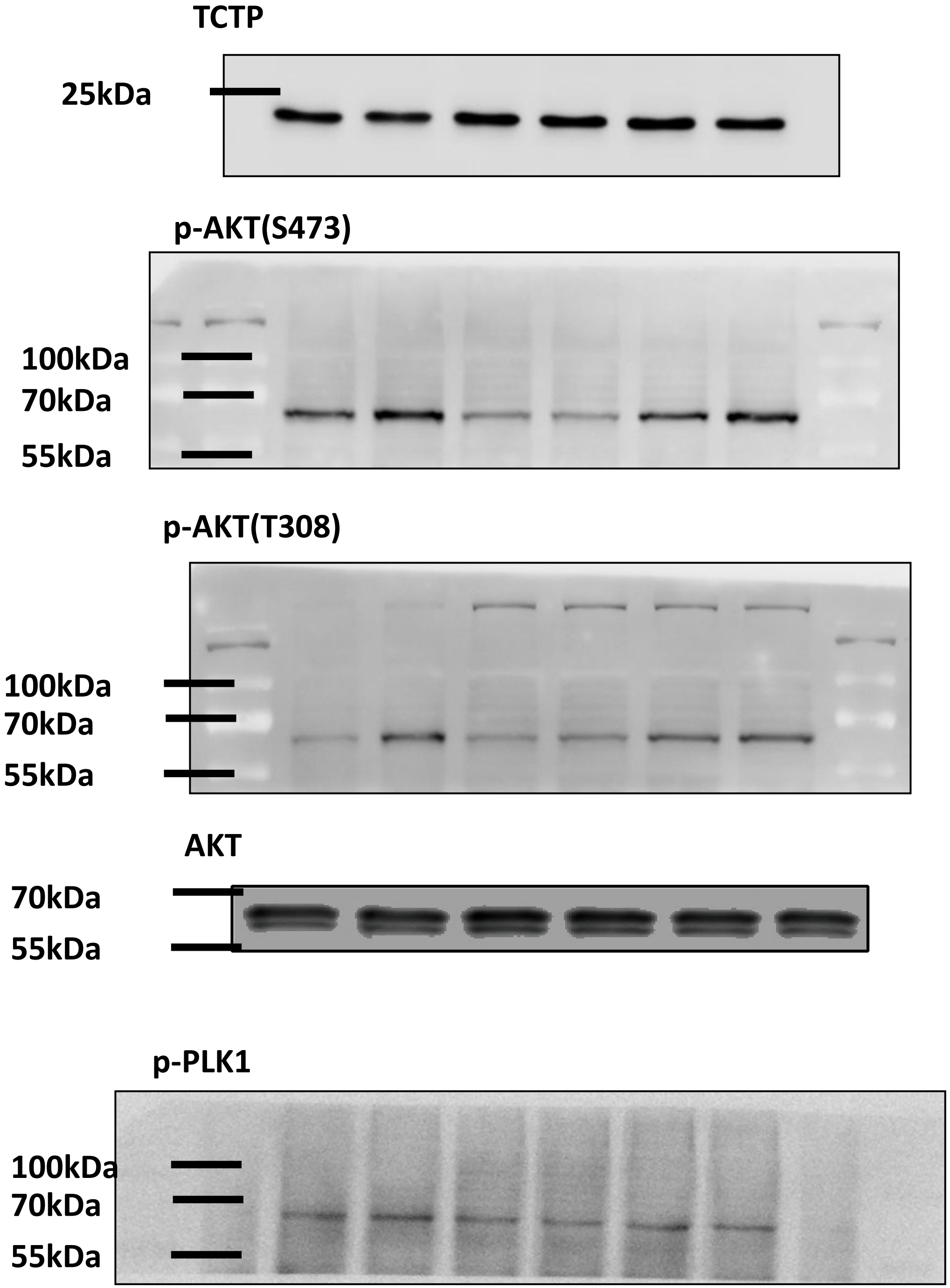

TCTP
25kDa
p-AKT(S473)
100kDa
70kDa
55kDa
p-AKT(T308)
100kDa
70kDa
55kDa
AKT
70kDa
55kDa
p-PLK1
100kDa
70kDa
55kDa

Figure 5C

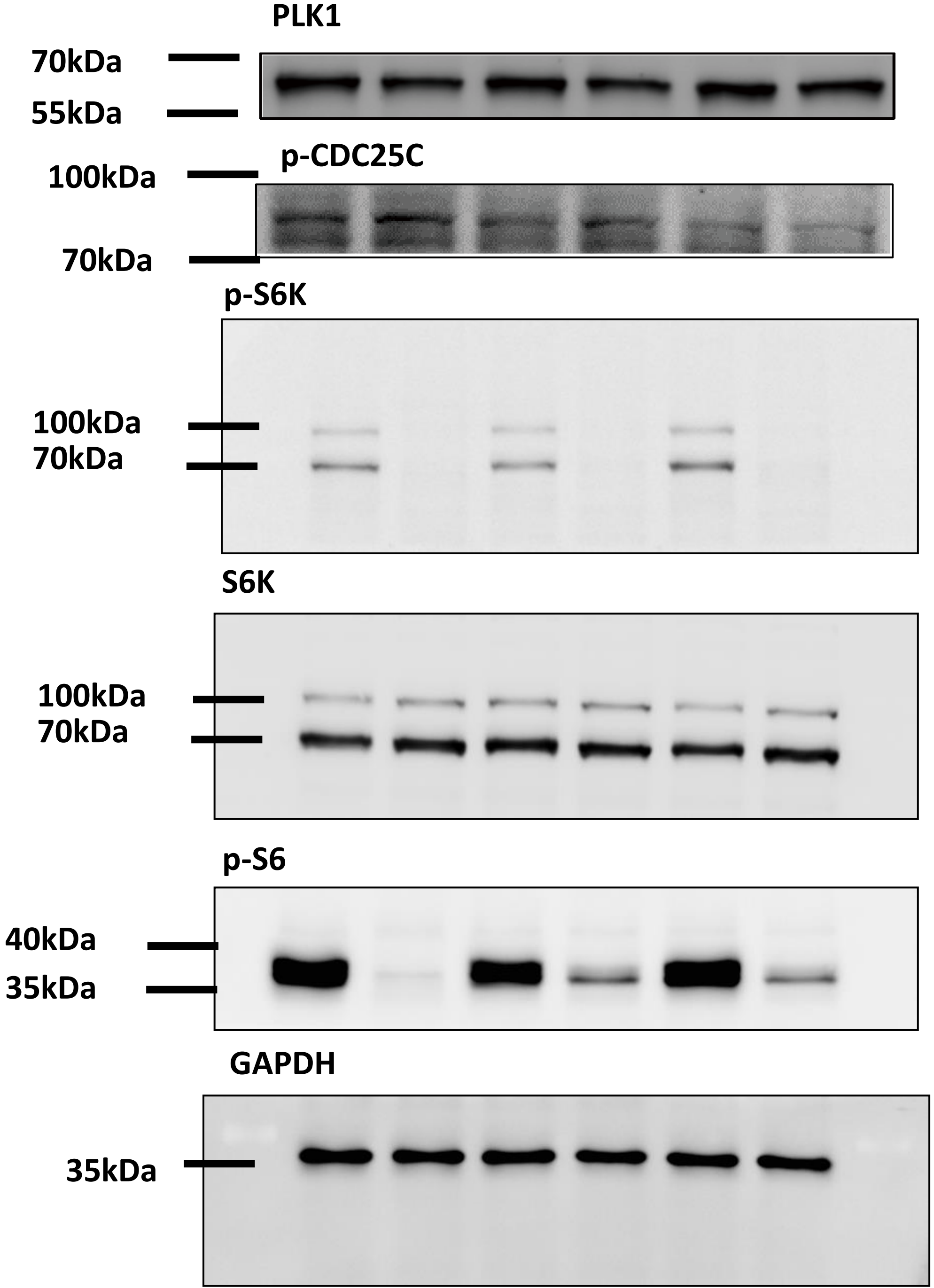

PLK1
70kDa
55kDa
p-CDC25C
100kDa
70kDa
p-S6K
100kDa
70kDa
S6K
100kDa
70kDa
p-S6
40kDa
35kDa
GAPDH
35kDa

Figure 5D

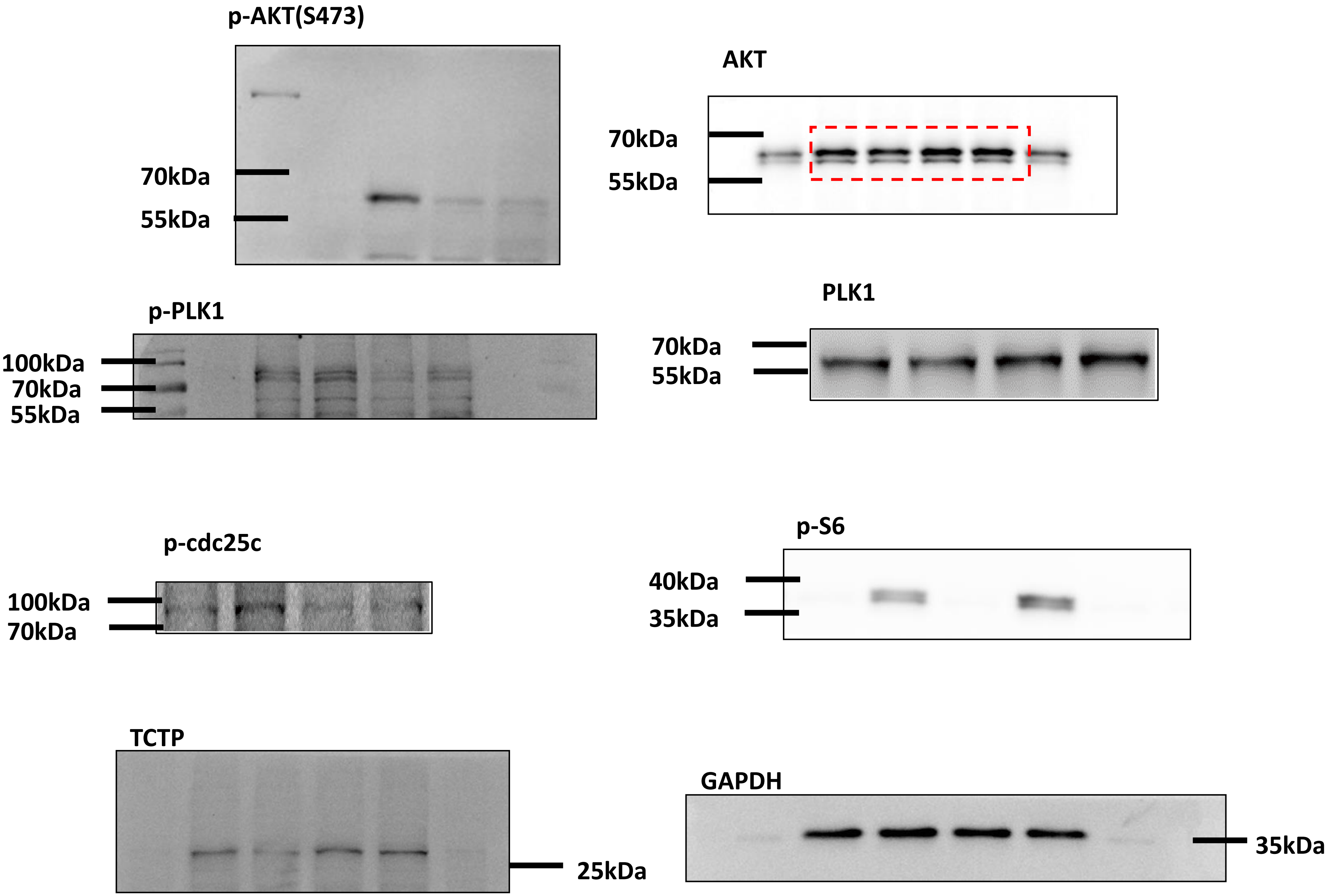
p-AKT(S473)
70kDa
55kDa
AKT
70kDa
55kDa
p-PLK1
100kDa
70kDa
55kDa
PLK1
70kDa
55kDa
p-cdc25c
100kDa
70kDa
p-S6
40kDa
35kDa
TCTP
25kDa
GAPDH
35kDa

Figure 5E

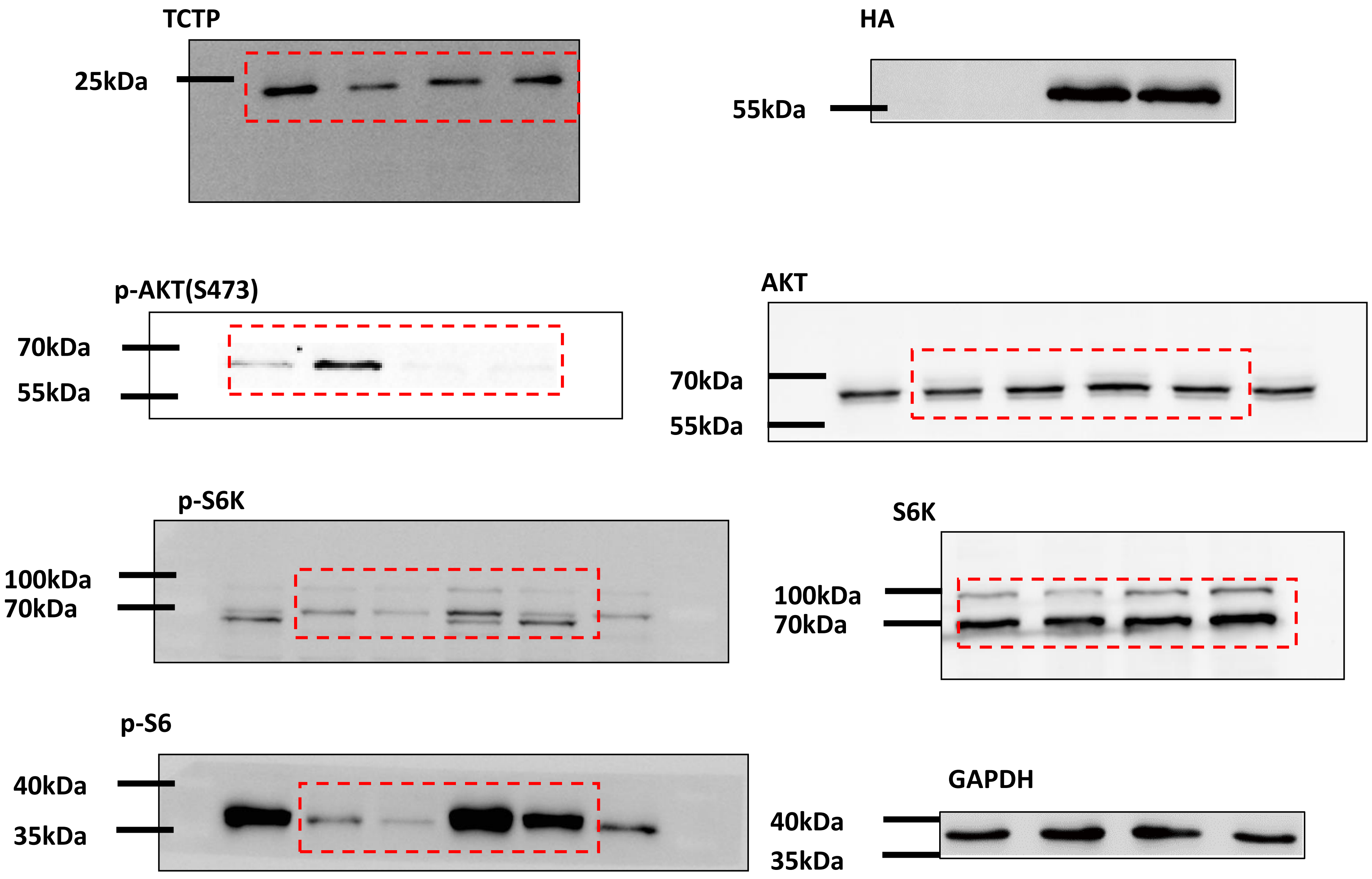
TCTP
25kDa
HA
55kDa
p-AKT(S473)
70kDa
55kDa
AKT
70kDa
55kDa
p-S6K
100kDa
70kDa
S6K
100kDa
70kDa
p-S6
40kDa
35kDa
GAPDH
40kDa
35kDa

Figure 6B

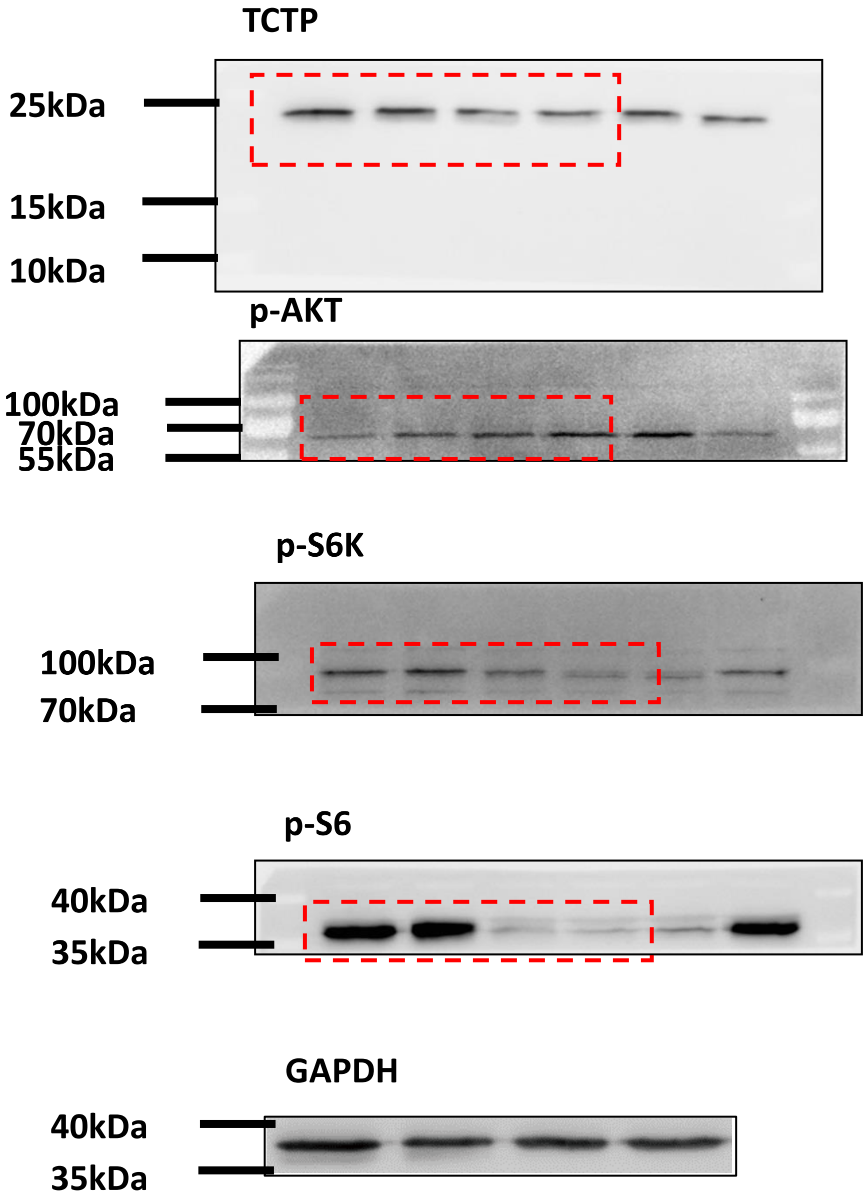
TCTP
25kDa
15kDa
10kDa
p-AKT
100kDa
70kDa
55kDa
p-S6K
100kDa
70kDa
p-S6
40kDa
35kDa
GAPDH
40kDa
35kDa

Figure 6D

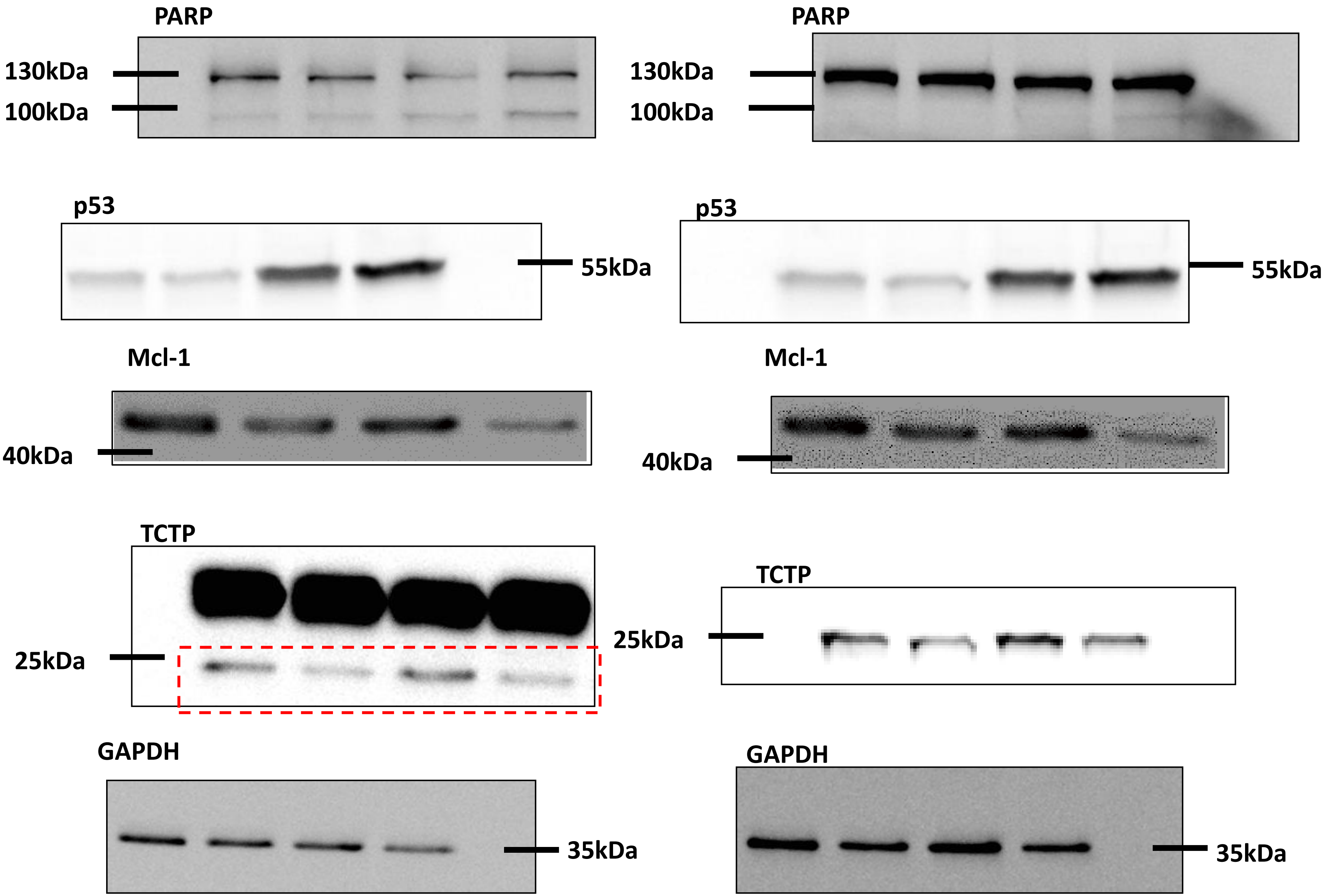
PARP
130kDa
100kDa
PARP
130kDa
100kDa
p53
55kDa
p53
55kDa
Mcl-1
40kDa
Mcl-1
40kDa
TCTP
25kDa
TCTP
25kDa
GAPDH
35kDa
GAPDH
35kDa

Figure 6G

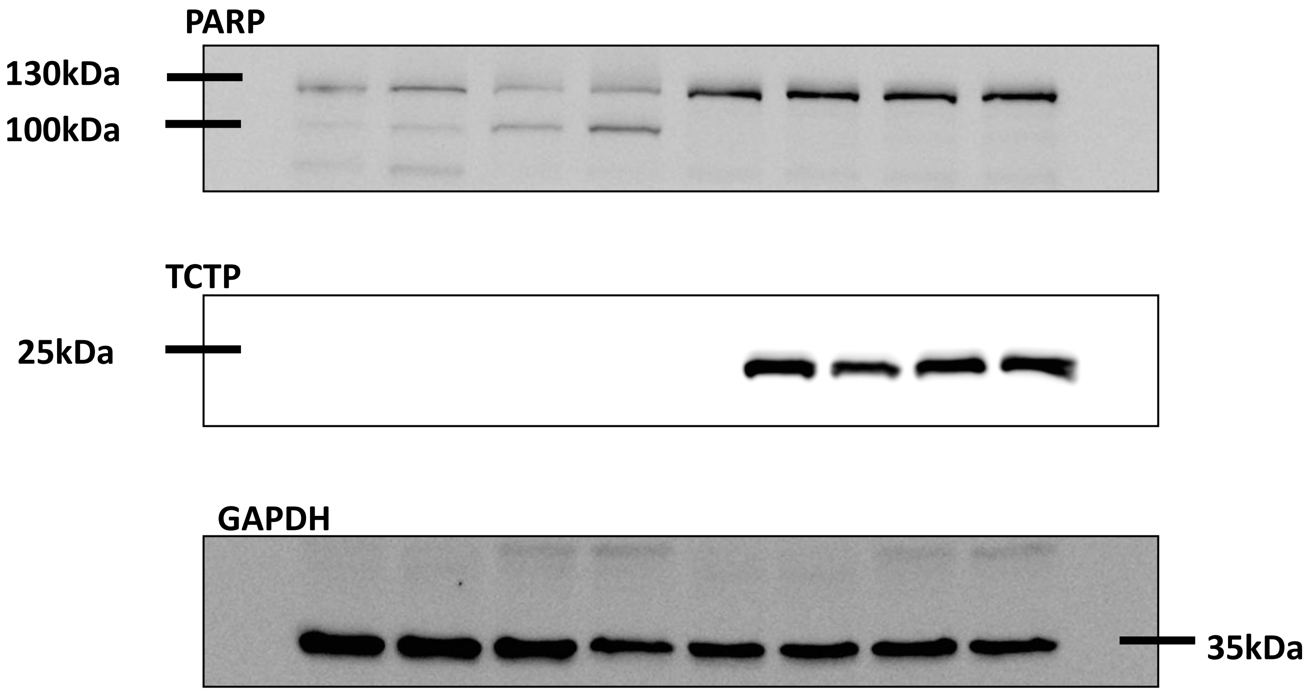

Supplementary Figure. S1

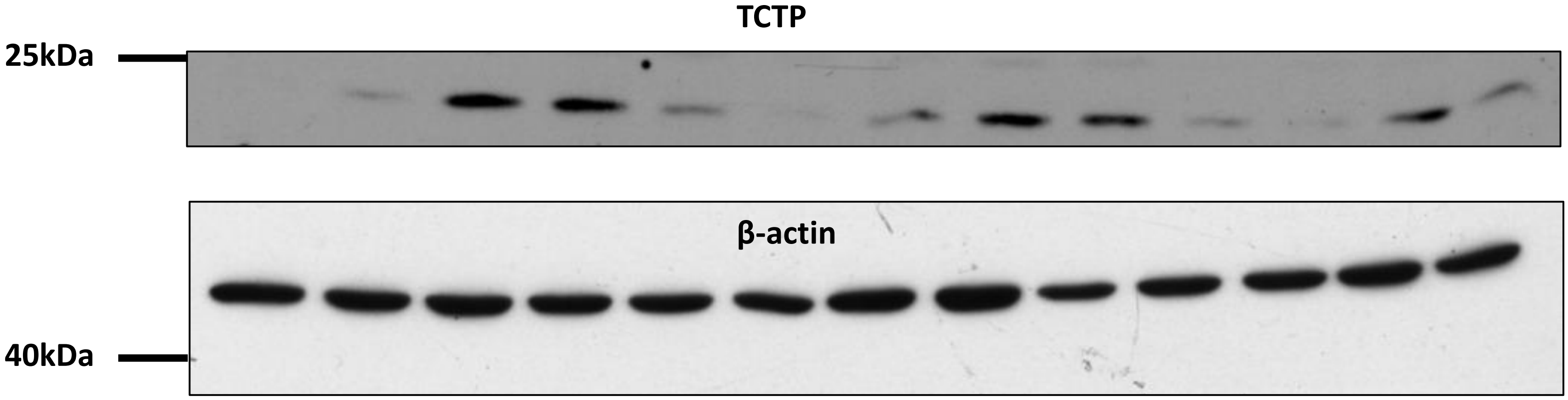

Supplementary Figure. S1 – replicate blot

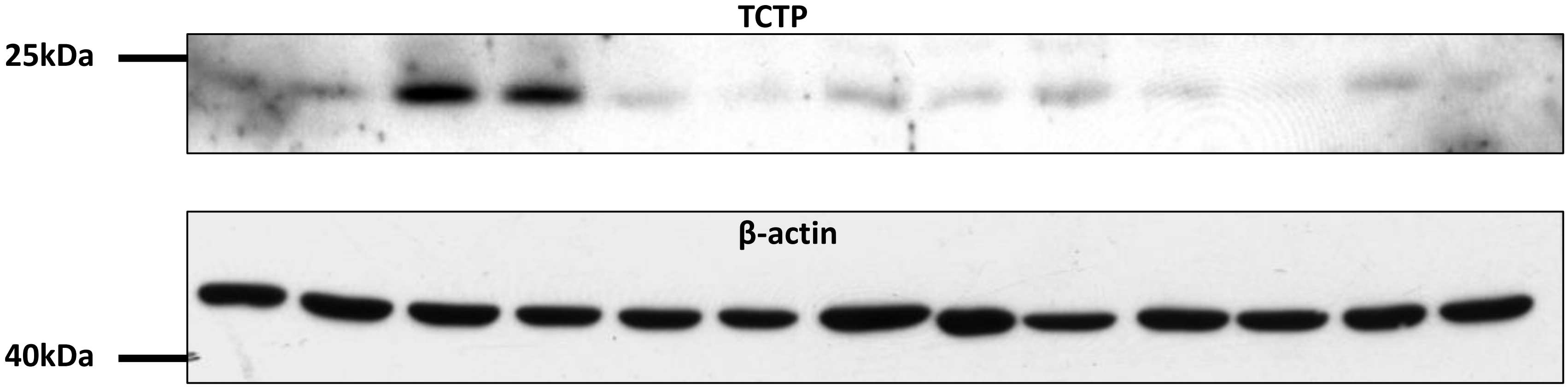

Supplementary Figure. S2A

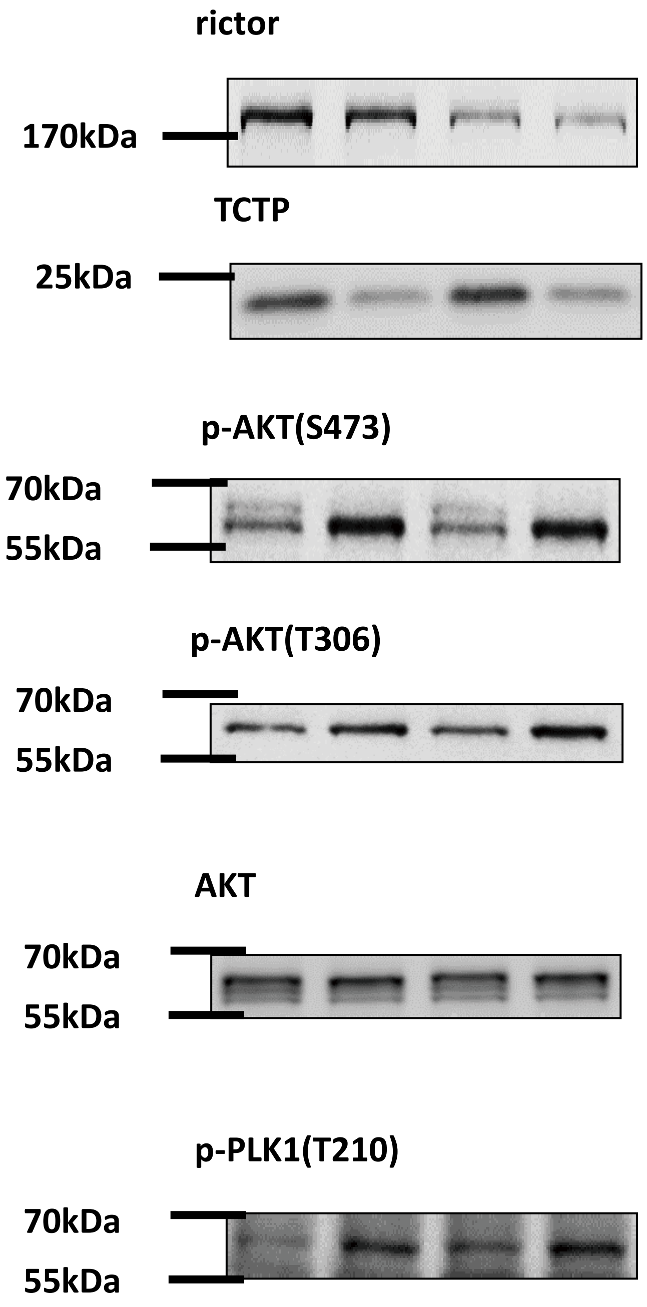

Supplementary Figure. S2A

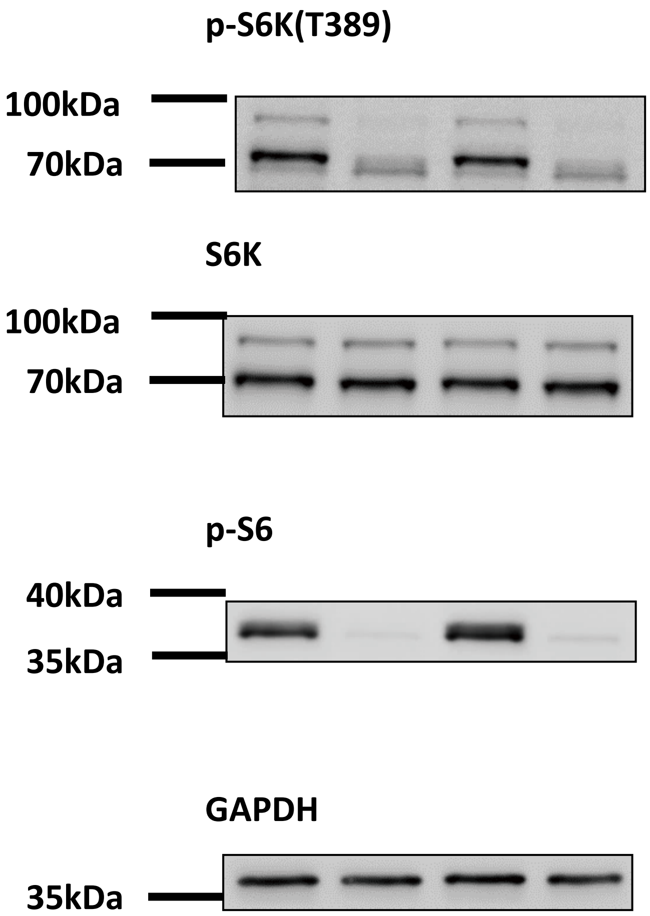

Supplementary Figure. S2A – replicate blot

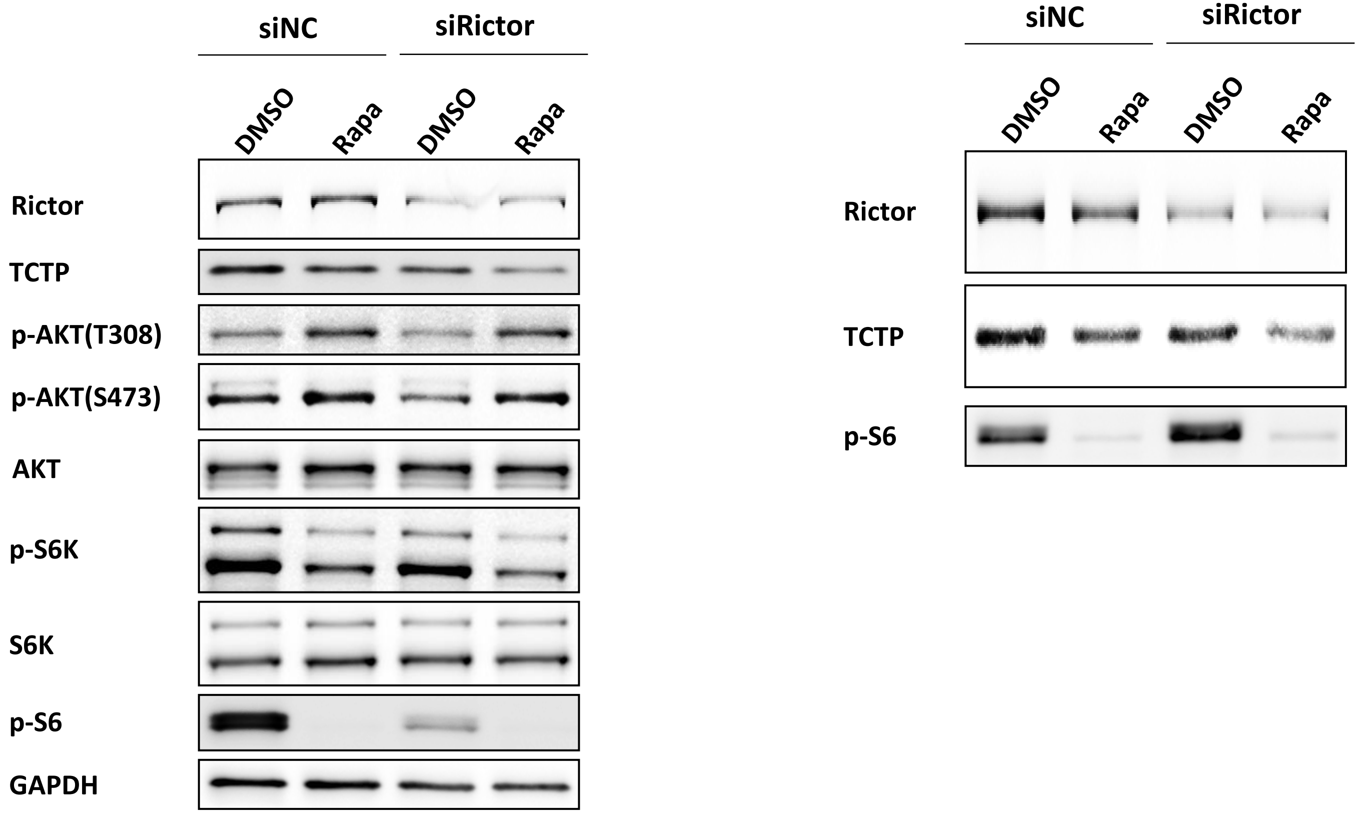

Supplementary Figure. S2B

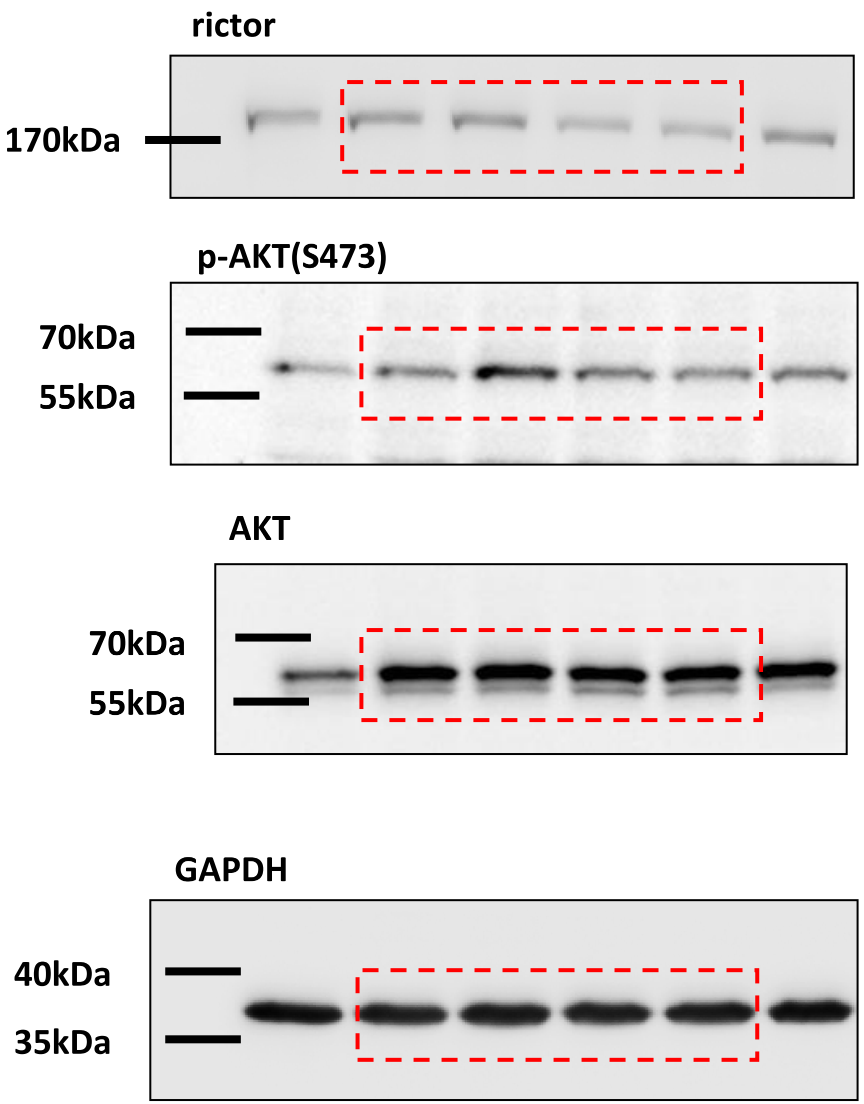

Supplementary Figure. S2B – replicate blot

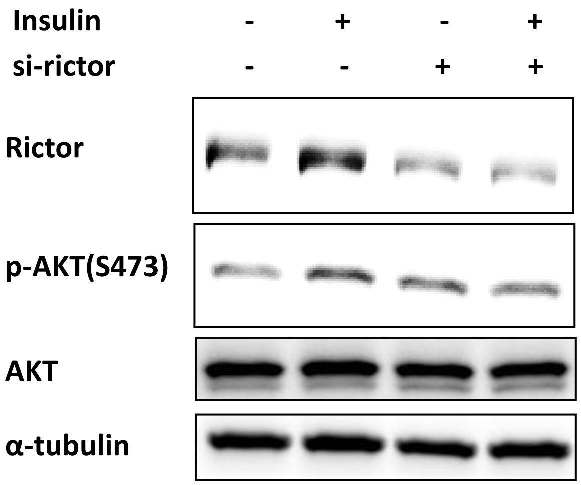

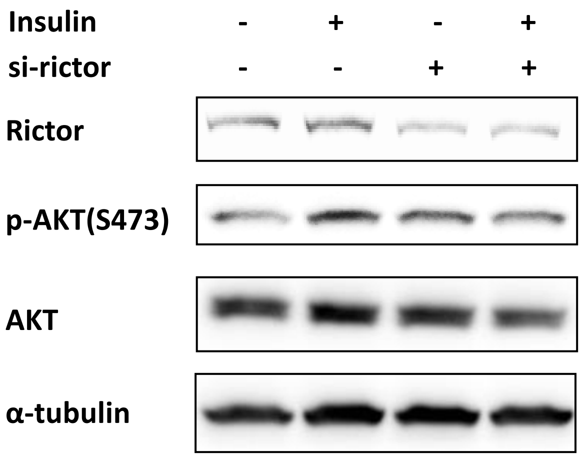

Supplementary Figure. S3A

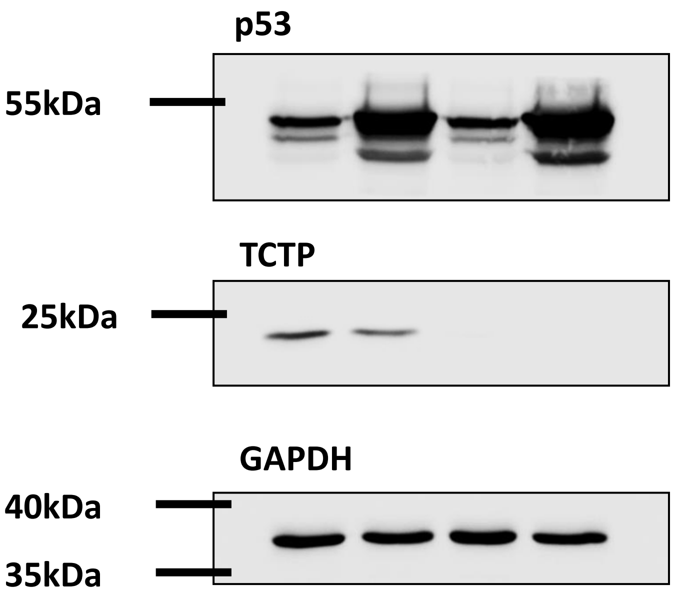

Supplementary Figure. S3B

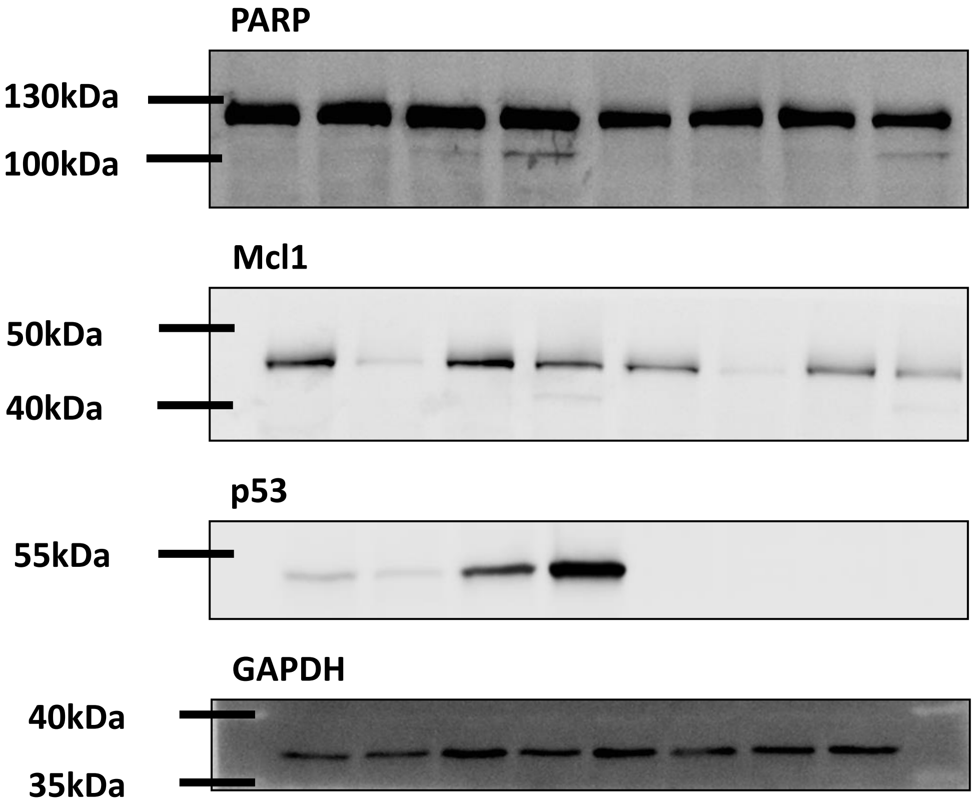

Supplementary Figure. S3B – replicate blot

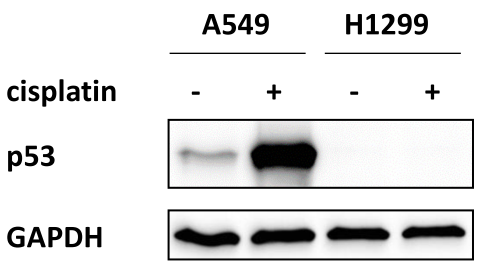

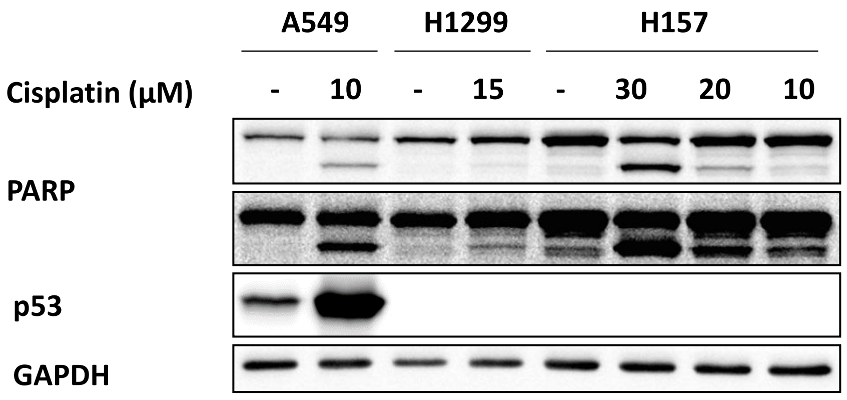

Supplementary Figure. S3C

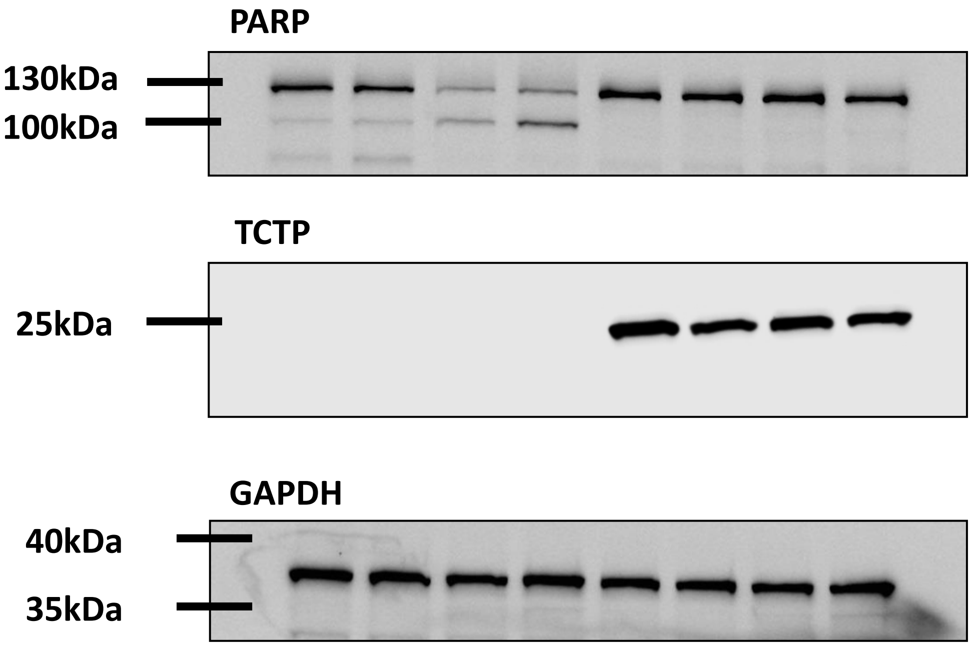

Supplementary Figure. S3C – replicate blot

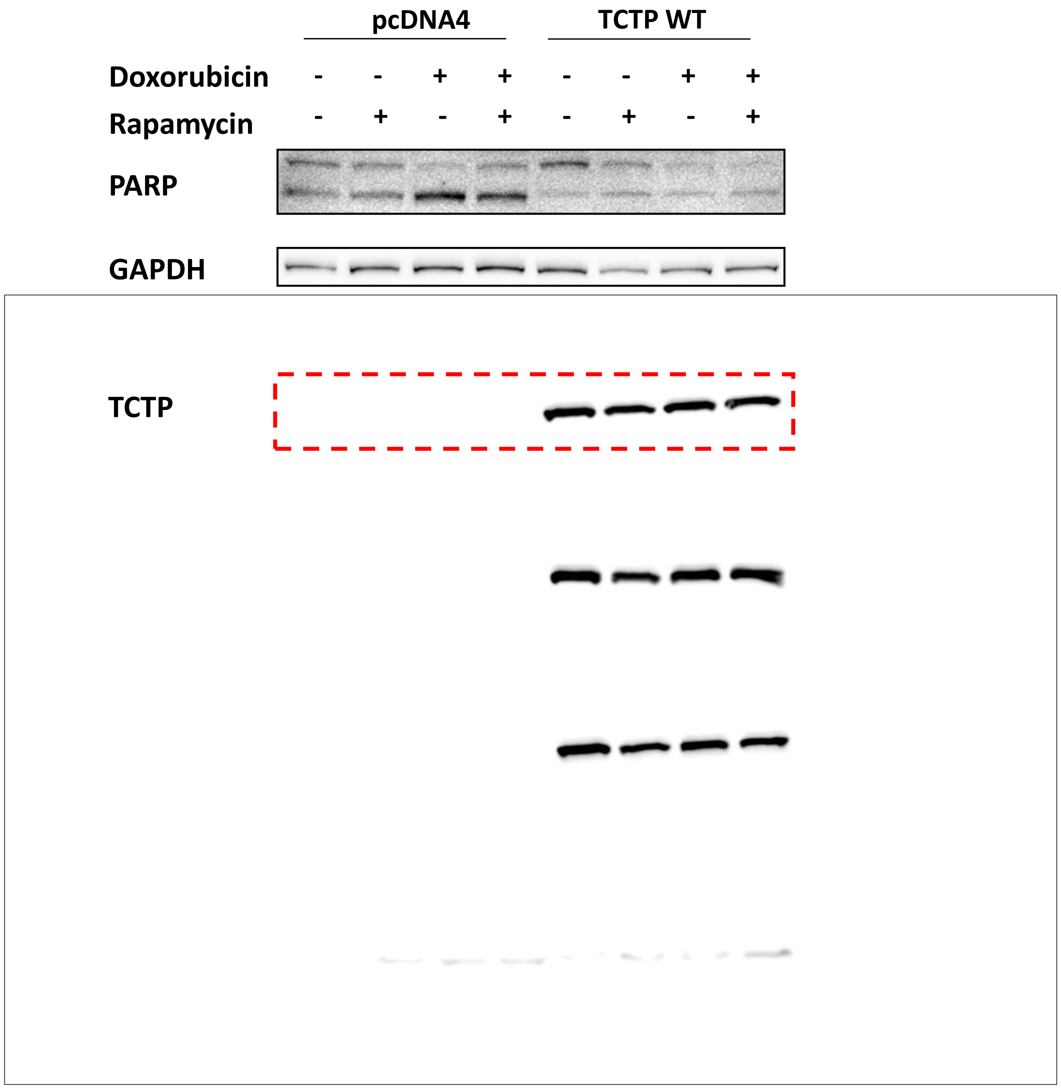

Supplimentary Figure. S4C

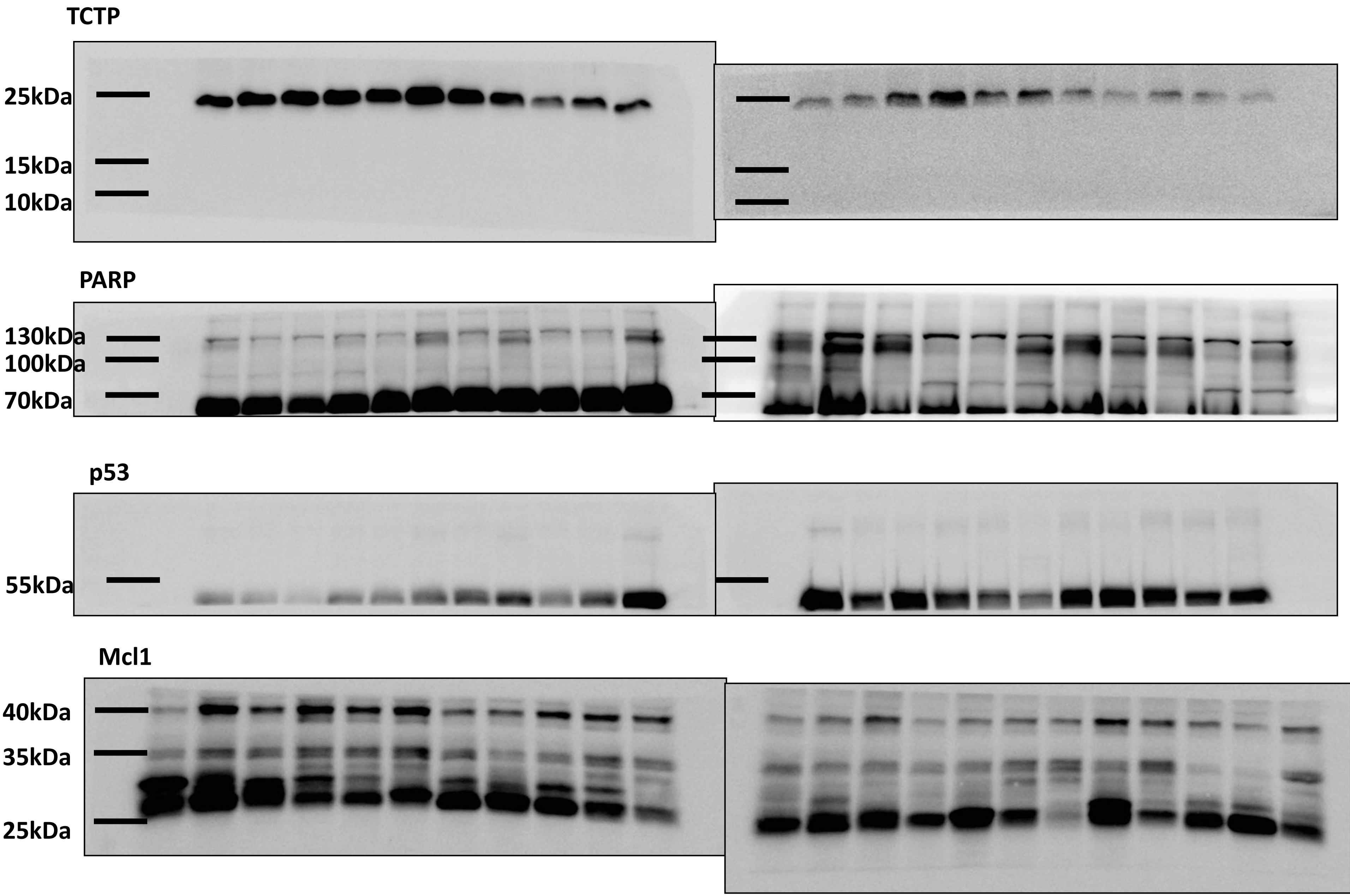

Supplimentary Figure. S4C

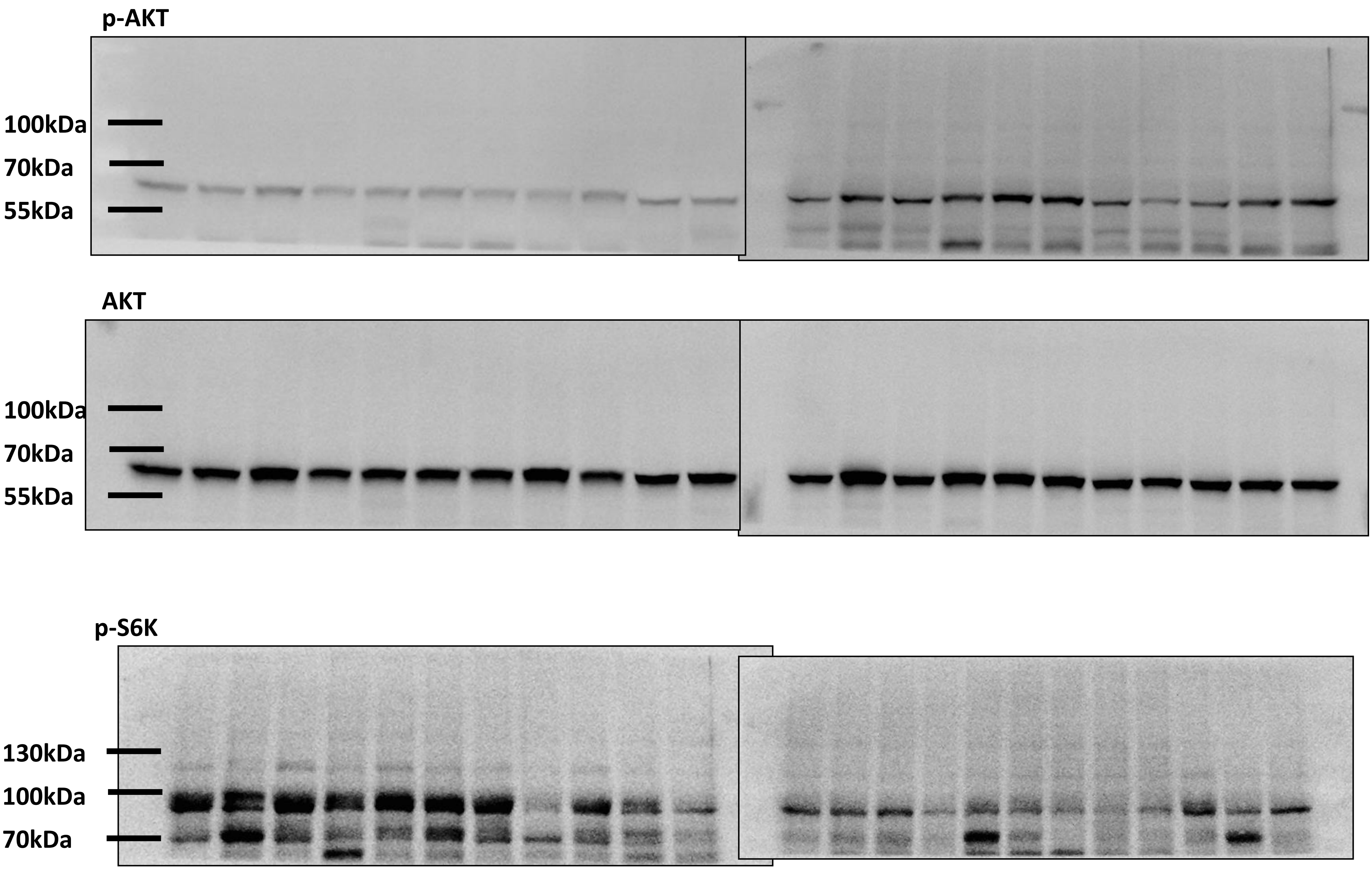

Supplimentary Figure. S4C

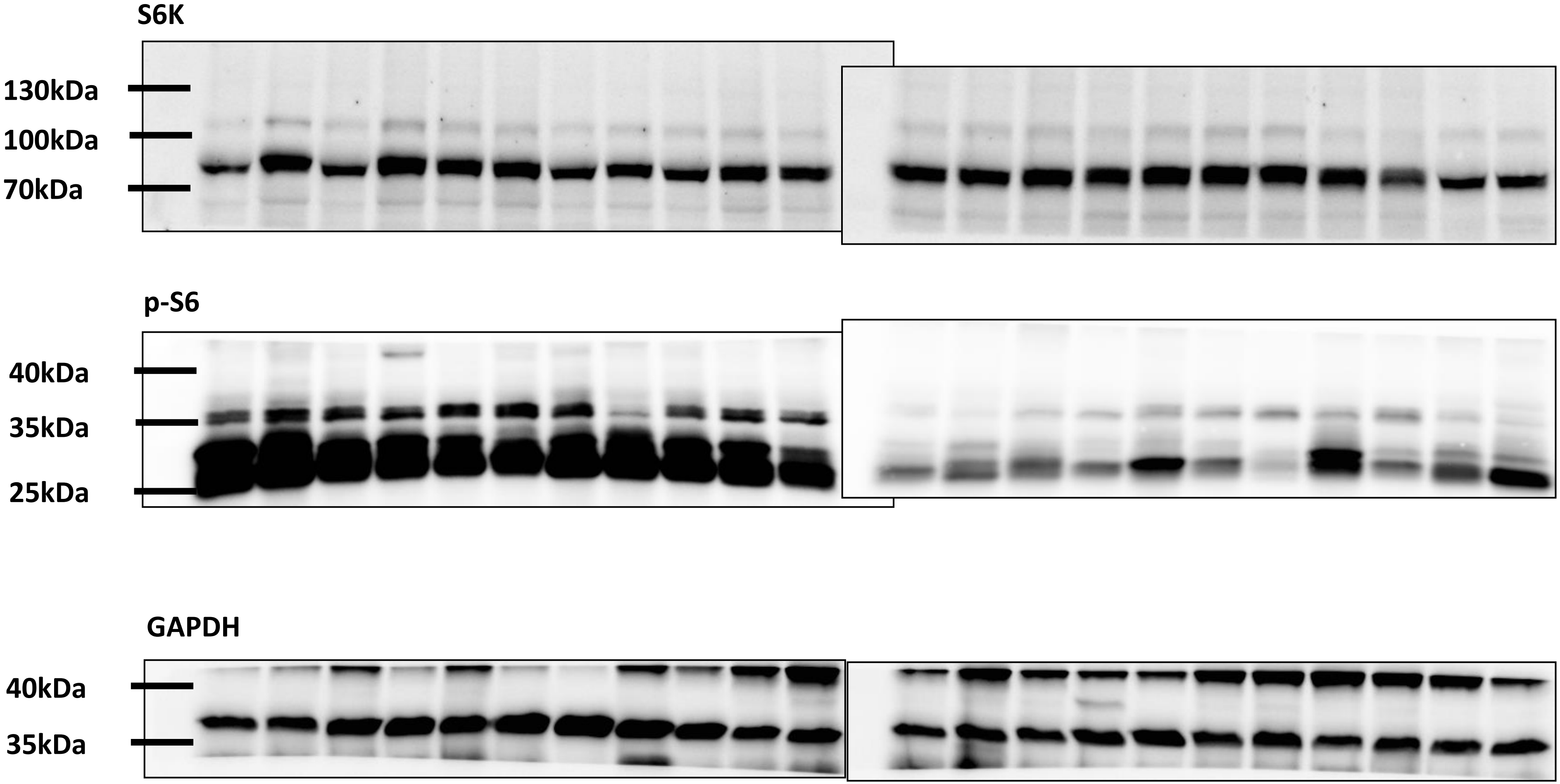

Supplimentary Figure. S4G

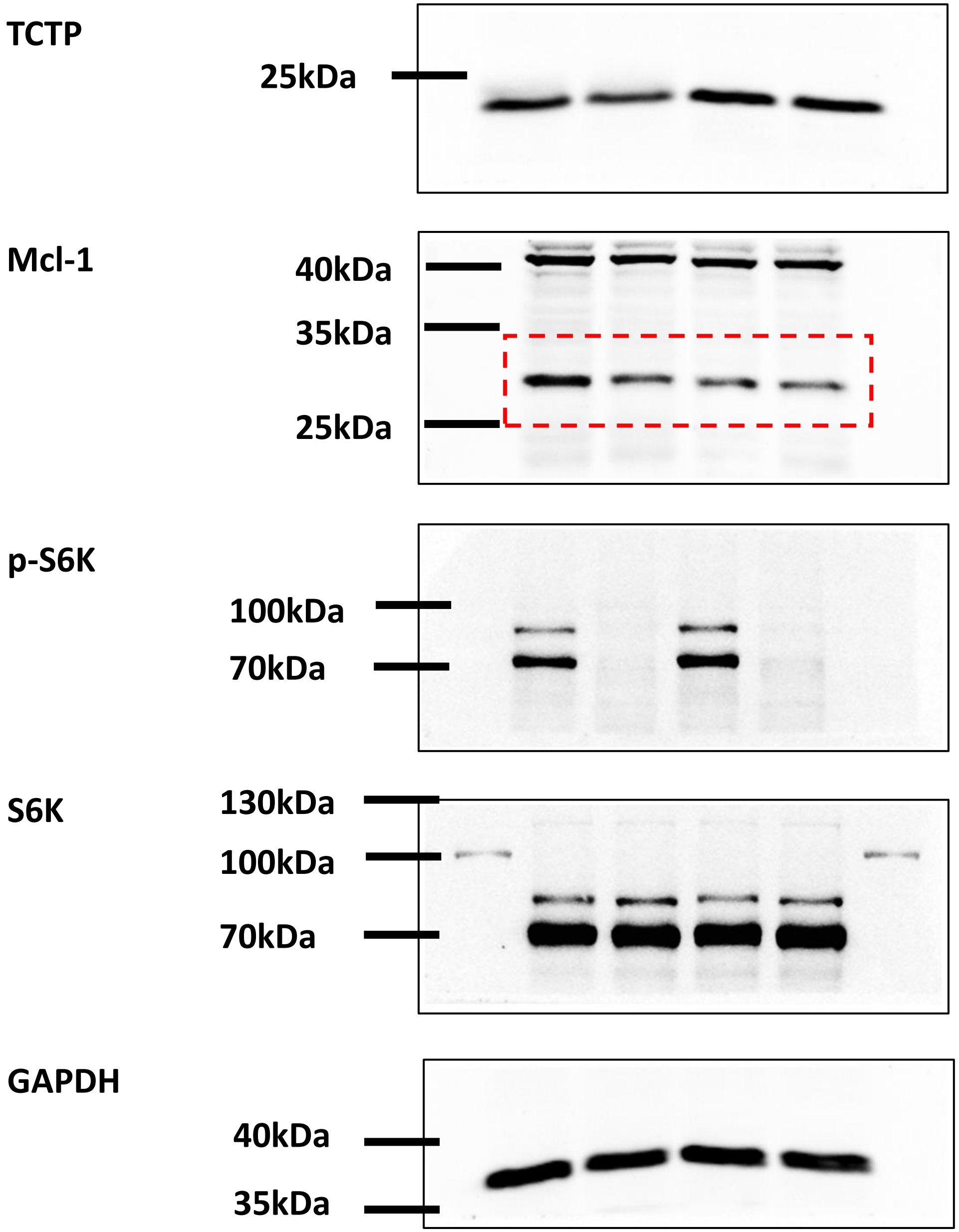

Supplement: Supplementary file 1 — Supplementary Information. [file 41598_2021_247_MOESM1_ESM.pdf]
